# Evolutionary origin of neohesperidoside, a bitter metabolite, and its potential role in biotic defense and citrus dissemination

**DOI:** 10.1016/j.xplc.2026.101697

**Published:** 2026-01-05

**Authors:** Gu Li, Huan Wen, Hanxin Zhou, Yuan Liu, Ziyu Yuan, Huixian Zhang, Zhehui Hu, Zeyang Liu, Huili Ma, Qi Chen, Guixiang Chen, Jia-Long Yao, Juan Xu, Jiajing Chen

**Affiliations:** 1 National Key Laboratory for Germplasm Innovation & Utilization of Horticultural Crops, College of Horticulture and Forestry Sciences, Huazhong Agricultural University, Wuhan 430070, P.R. China; 2 Hainan Research Institute of Huazhong Agricultural University, Sanya 572025, P.R. China; 3 New Zealand Institute for Bioeconomy Science Limited, Private Bag 92169, Auckland 1142, New Zealand; 4 Hubei Hongshan Laboratory, Wuhan 430070, P.R. China; 5 Sensory Evaluation and Quality Analysis Centre of Horticultural Products, Huazhong Agricultural University, Wuhan 430070, P.R. China

**Keywords:** citrus, bitterness, neohesperidoside, disaccharide glycosyltransferases, neofunctionalization, biotic defense

## Abstract

Fruit secondary metabolites play pivotal roles in plant evolution by deterring herbivores and attracting seed dispersers. However, the mechanisms by which these compounds evolve and drive diversification in citrus remain poorly understood. In this study, we demonstrate that the emergence of the bitter compound neohesperidoside (Neo) has contributed to citrus dissemination by enhancing defense against biotic stresses. Targeted metabolomic analyses revealed that Neo accumulation emerged in early-diverging citrus lineages, whereas its non-bitter counterpart rutinoside (Rut) can be traced back to *Citrus-*related species. Comparative genomic analyses and enzyme functional assays further revealed that Neo biosynthesis originated from the duplication of two di-glucosyltransferase genes, *CmdGlcT-1* and *UGT79B203*, in early-diverging citrus, followed by neofunctionalization into Cm1,2RhaT and UGT79B202, enzymes capable of synthesizing Neo. A structurally conserved amino acid residue—corresponding to Phe195 in Cm1,2RhaT and Leu201 in UGT79B203—was identified as critical for this functional transition. Compared with Rut, Neo exhibits stronger antifungal and anti-feeding activities, suggesting a role in enhanced biotic defense that may have contributed to the broader geographical distribution of early-diverging citrus species. Together, these findings provide new insights into the evolutionary origin of citrus bitterness and highlight the adaptive role of specialized metabolites in shaping plant–environment interactions.

## Teaser

This study demonstrates that the emergence of the bitter compound neohesperidoside in citrus resulted from the neofunctionalization of glucosyltransferases, driven by functional divergence at a key distal amino acid residue. The authors further show that this specialized metabolite enhances biotic defense, potentially contributing to the adaptive evolution and geographical expansion of citrus.

## Introduction

Flavonoids are phenolic secondary metabolites that have evolved in plants as adaptive responses to environmental challenges such as ultraviolet (UV) radiation, herbivory, pathogen attacks, and the need to attract pollinators (Tanaka et al., 2008; Cho and Lee, 2015; Peng et al., 2017; Trunschke et al., 2021; Forster et al., 2022; Sugimoto et al., 2022). In plants, flavonoids often accumulate as glycosides, which can be classified into monoglycosides and diglycosides (Shen et al., 2022). The latter are further categorized based on sugar composition and glycosidic linkage, with common types including neohesperidoside (Neo), rutinoside (Rut), gentiobioside (Gen), robinobioside (Rob), sophoroside (Sop), sambubioside (Sam), and apiosylrhamnoside (Api). Citrus species are particularly known for the accumulation of flavonoid disaccharide glycosides, represented by the bitter Neo, such as naringin, or the bitterless Rut, such as hesperidin (Li et al., 2022). Genomic analyses have categorized the orange subfamily (Rutaceae: Aurantioideae) into three major groups: *Citrus*-related genera, early-diverging citrus, and domesticated citrus (Huang et al., 2023). The *Citrus*-related genera group, including *Atalantia buxifolia*, was previously considered to represent primitive citrus species (Huang et al., 2018). The early-diverging citrus group contains wild species such as *Citrus* *trifoliata*, *Citrus* *mangshanensis,* and *Citrus ichangensis*. The domesticated citrus group experienced a complex history of admixture, and most modern citrus cultivars are derived from three basic species, *Citrus grandis* (pummelo), *Citrus reticulata* (mandarin), and *Citrus medica* (citron) (Wu et al., 2018). Neo compounds, which contribute to the primary bitterness of citrus fruits, accumulate at high levels in pummelo but are largely absent in mandarin and citron (Frydman et al., 2004; Chen et al., 2019; Li et al., 2022). This gain and loss of Neo biosynthetic capacity during citrus evolution makes citrus an excellent model for investigating the evolution and domestication of flavonoid-derived bitterness.

Neo is synthesized through rhamnosylation of flavonoid 7-*O*-glucosides, a reaction that elongates the sugar chain and often imparts distinct biological functions and flavor properties to flavonoids. For example, Neo formation results in a pronounced bitter taste, whereas 2″-*O*-rhamnosylation of sterol 3-*O*-glucosides (e.g., Polyphyllin I and Polyphyllin H) has been linked to enhanced antifungal activity in *Paris polyphylla* (Chen et al., 2023). These rhamnosylation reactions are predominantly catalyzed by UDP-dependent glycosyltransferases (UGTs), particularly a subgroup known as disaccharide-forming UGTs (dGlyTs), which are phylogenetically distinct from monosaccharide UGTs (Wilson and Tian, 2019). In pummelo, 1,2-rhamnosyltransferase (Cm1,2RhaT) has been reported to catalyze the formation of Neo from flavanone and flavone substrates (Frydman et al., 2004). Additionally, the related di-glucosyltransferase CmdGlcT-1, which shares a common evolutionary origin with Cm1,2RhaT, exhibits distinct sugar-donor specificity and catalyzes the formation of glucosyl–glucoside disaccharides (Chen et al., 2019). Previous studies suggested that artificial selection against bitterness contributed to the loss of the *1,2RhaT* gene in sweet oranges (Chen et al., 2019). However, the genetic basis underlying *1,2RhaT* evolution and bitterness formation in citrus remains unclear.

Amino acid substitutions in enzymes can give rise to novel enzymatic activities, leading to the emergence of new specialized metabolites. In glycosyltransferases, single-amino-acid changes have been shown to alter sugar-donor or sugar-acceptor specificity, thereby expanding the diversity of glycosylated metabolites (Chen and Li, 2016; Zhang et al., 2021). When such metabolic innovations confer ecological advantages, such as enhanced defense or increased stress tolerance, they may be favored by natural selection, leading to the fixation of relevant genetic variants in plant populations (Noda-Garcia et al., 2018). Flavonoids are generally associated with antioxidant activity and protection against temperature stress, whereas bitter compounds frequently deter herbivores and pests (Thodberg et al., 2018; Gouot et al., 2019; Wang et al., 2022). Despite extensive research on Neo biosynthesis and accumulation across citrus species, the ecological functions of Neo in shaping interactions between citrus and their environment remain largely unexplored. Investigating how Neo influences plant–environment interactions could therefore provide valuable insights into the selective pressures driving the evolution of specific plant metabolites.

Here, we performed targeted metabolomic analyses on 38 accessions representing *Citrus*-related genera, early-diverging citrus, and domesticated citrus. We found that the accumulation of bitter Neo originated in early-diverging citrus species. Enzyme functional validation combined with comparative genomic analyses revealed that duplication and neofunctionalization of two di-glucosyltransferase genes in early-diverging citrus enabled the transition from 2″-*O*-glucosylation to 2″-*O*-rhamnosylation, thereby facilitating Neo biosynthesis. A key amino acid residue was identified as critical for this neofunctionalization. Furthermore, bitter Neo exhibited stronger antifungal and anti-herbivory effects than its Rut counterpart. Given the broader geographical distribution of early-diverging citrus compared to *Citrus*-related genera, we propose that Neo may have contributed to adaptive evolution by enhancing defense against biotic stresses. Overall, this study provides new insights into the evolution of citrus bitterness and highlights the role of specialized metabolites in mediating plant–environment interactions.

## Results

### Accumulation of bitter neohesperidoside began in early-diverging citrus species

**Figure 1:**
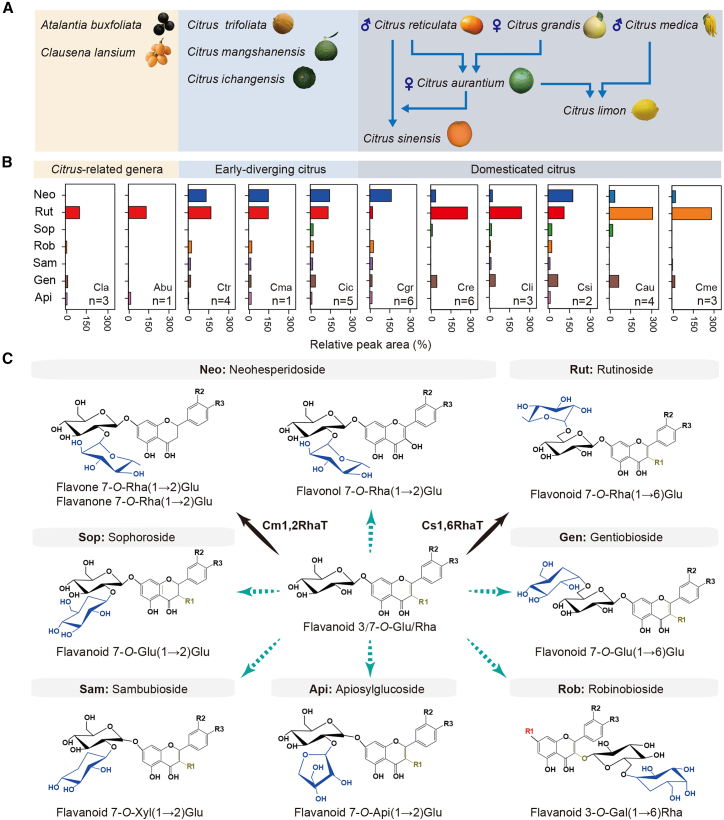
Flavonoid disaccharide glycoside metabolome among different citrus and *Citrus*-related species. **(A)** Phylogenetic relationships of the species analyzed in this study. *Clausena lansium* (Cla) and *Atalantia buxifolia* (Abu) belong to *Citrus*-related genera; *Citrus trifoliata* (Ctr), *C. ichangensis* (Cic), and *C. mangshanensis* (Cma) represent early-diverging citrus; *C. grandis* (Cgr), *C. reticulata* (Cre), *C. medica* (Cme), *C. aurantium* (Cau), *C. sinensis* (Csi), and *C. limon* (Cli) represent domesticated citrus. **(B)** Levels of seven flavonoid disaccharide glycosides in fruit flavedo from different species. Relative levels are presented as means of three biological replicates based on log2-transformed ion pair peak areas. “n” represents the number of accessions analyzed for each species. Neo, neohesperidosides; Rut, rutinosides; Sop, sophorosides; Rob, robinobiosides; Sam, sambubiosides; Gen, gentiobiosides; Api, apiosides. **(C)** Molecular structures of seven representative disaccharide glycosides detected across species. Solid lines indicate that the corresponding biosynthetic enzyme has been characterized in citrus; dashed lines indicate that the relevant enzyme has not yet been characterized.

To characterize flavonoid variation during citrus evolution, a widely targeted metabolomic analysis was performed using four accessions of *Citrus*-related species (*A. buxifolia* and *Clausena lansium*), 10 accessions of early-diverging citrus species (*C. trifoliata*, *C. ichangensis*, and *C. mangshanensis*), and 24 accessions representing six domesticated citrus species (*C. grandis*, *C. reticulata*, *C. medica*, *Citrus aurantium*, *Citrus sinensis*, and *Citrus limon*) (Figure 1A; Supplemental Table 1). This analysis identified 67 flavonoid compounds, including seven aglycones, 21 monoglycosides, and 39 disaccharide glycosides (Supplemental Data 1). The results showed that *A. buxifolia* accumulated predominantly monoglycoside flavonoids, whereas citrus species accumulated mainly disaccharide glycosides and showed higher total flavonoid levels (Supplemental Figure 1). Heatmap analysis further revealed that the accumulation of these glycoside types displayed germplasm-specific patterns (Supplemental Figure 2).

We then examined the distribution and abundance of seven disaccharide glycoside types across the surveyed species. Neo and/or Rut were the predominant disaccharide glycosides. Rut was already present in *Citrus*-related species, whereas the accumulation of the bitter Neo emerged in early-diverging citrus species and was associated with a marked increase in total disaccharide glycoside content (Figure 1B; Supplemental Data 2). In domesticated citrus, the accumulation of Neo and Rut was species dependent. Neo was more abundant in pummelo (*C. grandis*), whereas Rut predominated in mandarin-derived species (*C. reticulata*, *C. sinensis*, and *C. limon*), as well as in citron (*C. medica*). Notably, sour orange (*C. aurantium*), a mandarin–pummelo hybrid, accumulated high levels of both Neo and Rut. In contrast, the remaining disaccharide glycoside types were consistently detected at low abundance across citrus species. Together, these findings indicate that Neo accumulation originated and became fixed in early-diverging citrus lineages, representing a key metabolic innovation during citrus evolution.

Structural analysis of the identified disaccharide glycosides revealed clear patterns in sugar linkage types and composition. Gen and Rob, similar to Rut, are characterized by β1–6 glycosidic linkages; however, Gen contains a glucosyl–glucose moiety, whereas Rob harbors a galactosyl–rhamnose moiety. The remaining three disaccharide glycosides are connected via β1–2 linkages, similar to Neo, but differ in their sugar composition. Specifically, Sop, Sam, and Api incorporate glucosyl, xylosyl, and apiosyl moieties, respectively, as the second sugar attached to the flavonoid glucoside acceptor (Figure 1C).

Among these glycosides, Neo is synthesized by 1,2RhaT and Rut by 1,6RhaT, whereas the enzymes responsible for synthesizing the remaining disaccharides have not yet been identified. The previously characterized 1,2RhaT (Cm1,2RhaT; accession no. AY048882) was reported to lack activity toward flavonol substrates (Frydman et al., 2004). However, the detection of flavonol neohesperidosides—such as isorhamnetin 3-*O*-neohesperidoside—in pummelo and sour orange suggests the existence of a distinct 1,2RhaT capable of catalyzing neohesperidoside formation in citrus (Supplemental Data 1).

### Two pairs of paralogous *dGlyT* genes are associated with neohesperidoside biosynthesis in citrus

**Figure 2:**
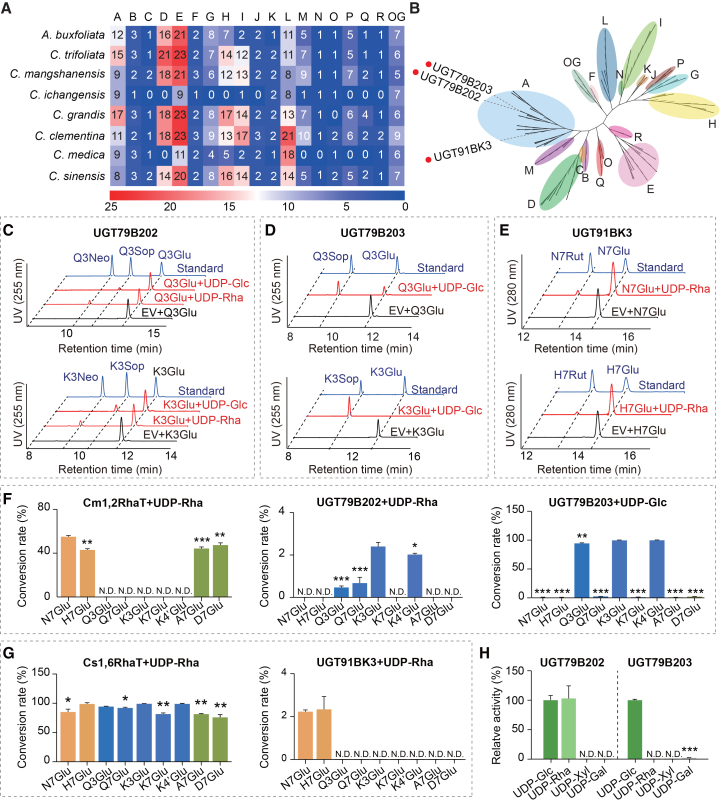
Functional characterization and substrate specificity of citrus dGlyTs. **(A)** Heatmap showing the number of UGT genes identified in each UGT phylogenetic group (A–N and the outgroup [OG]) across eight citrus reference genomes. **(B)** Phylogenetic tree constructed using protein sequences of putative UGTs identified from the *C. grandis* cv. Wanbaiyou reference genome, showing different UGT groups. Group A contains functionally characterized dGlyTs. The tree was adapted from Wilson and Tian (2019). The three dGlyTs functionally characterized in this study are highlighted with red dots. **(C–E)** HPLC elution profiles of compound standards (blue), reaction mixtures catalyzed by recombinant UGT79B202 **(C)**, UGT79B203 **(D)**, and UGT91BK3 **(E)** (red lines), and reaction mixtures catalyzed by recombinant protein from the empty vector (black lines). **(Fand G)** Substrate specificity of enzymes catalyzing β1 → 2 linkages **(F)** and β1 → 6 linkages **(G)** in the disaccharide moiety. **(H)** Sugar-donor specificity of UGT79B202 and UGT79B203. N7Glu, naringenin 7-*O*-glucoside; H7Glu, hesperetin 7-*O*-glucoside; Q3Glu, quercetin 3-*O*-glucoside; Q7Glu, quercetin 7-*O*-glucoside; K3Glu, kaempferol 3-*O*-glucoside; K7Glu, kaempferol 7-*O*-glucoside; K4′Glu, kaempferol 4′-*O*-glucoside; A7Glu, apigenin 7-*O*-glucoside; D7Glu, diosmetin 7-*O*-glucoside; UDP-Glc, UDP-glucose; UDP-Rha, UDP-rhamnose. Error bars represent standard deviations from three biological replicates. Statistical significance was assessed using Student’s *t*-test by comparison with the substrate showing the highest conversion rate. ∗*p* < 0.05, ∗∗*p* < 0.01, ∗∗∗*p* < 0.001.

To identify dGlyTs potentially involved in citrus Neo metabolism, we performed a phylogenetic analysis of UGT proteins identified from the reference genomes of eight *Citrus* and *Citrus*-related species. Among these species, *C. grandis* contained the largest number of UGTs, with 17 members belonging to phylogenetic group A, which represents the main branch of dGlyTs (Figures 2A and 2B). Of these, 13 genes possessed intact open reading frames in *C. grandis* Wanbaiyou, whereas the remaining four genes—including a homolog of *Cs1,6RhaT* (accession no. DQ119035)—were prematurely terminated due to frameshift mutations (Supplemental Data 3). Recombinant proteins corresponding to the 13 intact *UGTs* were heterologously expressed in *Escherichia coli* and purified for subsequent functional characterization.

Enzymatic activities of the recombinant proteins were assessed using nine flavonoid glucosides (including flavanones, flavonols, and flavones) as acceptor substrates, together with UDP-Glc, UDP-Rha, UDP-Xyl, and UDP-Gal as sugar donors (Supplemental Figure 3). This screening identified three novel UGTs with dGlyT activity. UGT79B202 (Cg2g042800) exhibited dual 1,2RhaT and 1,2GlcT activities, catalyzing 2″-glycosylation and 2″-rhamnosylation of Q3Glu and K3Glu to produce the corresponding Sop and Neo, respectively (Figure 2C). UGT79B203 (Cg2g042810) was identified as a 1,2GlcT, catalyzing 2″-glycosylation of Q3Glu and K3Glu to generate Sop (Figure 2D). In addition, UGT91BK3 (Cg5g041670) catalyzed 1,6-rhamnosylation of N7Glu and H7Glu to generate the corresponding Rut compounds (Figure 2E).

Substrate specificities of UGT79B202, UGT79B203, and UGT91BK3 were further evaluated alongside Cm1,2RhaT and Cs1,6RhaT. Among enzymes catalyzing β1–2 glycosidic linkages, Cm1,2RhaT demonstrated strong activity toward flavanone and flavone substrates, with conversion rates ranging from 42.98% to 54.98%, but showed no detectable activity toward flavonol substrates (Figure 2F; Supplemental Table 4). In contrast, UGT79B202 exhibited strict substrate specificity for flavonols, albeit with modest conversion rates (0.34%–2.47%) (Figure 2F; Supplemental Figures 4 and 5; Supplemental Table 4). Both Cm1,2RhaT and UGT79B202 catalyzed Neo formation but exhibited complementary substrate preferences. UGT79B203 showed strong activity in converting flavonol glucosides (Q3Glu, K3Glu, and K4′Glu) into their corresponding sophorosides, with conversion rates of 94.34%–99.57%, but exhibited only weak activity toward flavanones and flavones (0.55%–2.51%) (Figure 2F; Supplemental Figures 6 and 7; Supplemental Table 4).

Despite sharing 78.26% amino acid identity and clustering closely in the phylogenetic tree (Figure 2B), UGT79B202 and UGT79B203 differed markedly in sugar-donor specificity. A similar functional divergence was observed between Cm1,2RhaT and CmdGlcT-1, which share 85.84% amino acid identity. These observations suggest that two phylogenetically related yet functionally distinct dGlyT pairs may have co-evolved and contributed to the emergence of bitter Neo biosynthesis.

Among enzymes catalyzing β1 → 6 glycosidic linkages, Cs1,6RhaT demonstrated broad substrate promiscuity, catalyzing all tested substrates with high conversion rates (75.64%–99.04%). In contrast, UGT91BK3 showed weak activity that was restricted to flavanone substrates, with conversion rates of only 2.23%–2.34% (Figure 2G; Supplemental Figure 8; Supplemental Table 4). Although both enzymes catalyze Rut biosynthesis, they share only 30.66% amino acid sequence identity, indicating that they function as distinct isoenzymes acting in concert.

Finally, sugar-donor preferences of UGT79B202 and UGT79B203 were examined using their optimal acceptor substrates. Although UGT79B202 showed comparable conversion rates with UDP-Rha and UDP-Glc in endpoint assays (Figure 2H), kinetic analyses revealed a 4.9-fold higher catalytic efficiency, measured as *k*_cat_/*K*_m_, for UDP-Rha compared with UDP-Glc (Supplemental Figure 9). In addition, UGT79B203 exhibited trace activity toward UDP-Gal, corresponding to 2.17% of its activity with UDP-Glc (Figure 2H), whereas no detectable activity toward alternative sugar donors was observed for the remaining dGlyTs.

### Duplication and functional diversification of *dGlyT* genes in early-diverging citrus led to the production of bitter neohesperidosides

**Figure 3:**
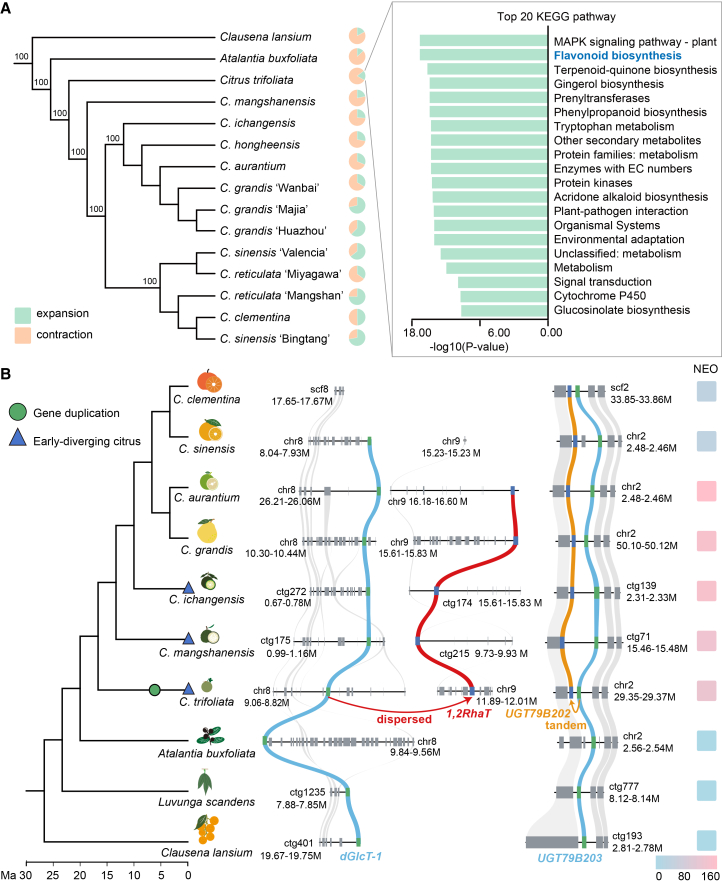
Gene family expansion and evolutionary analysis of two *dGlyT* pairs related to neohesperidoside biosynthesis. **(A)** Phylogenetic tree constructed using protein sequences of single-copy orthologous genes identified from reference genomes of 15 *Citrus* and *Citrus*-related species (left panel). Kyoto Encyclopedia of Genes and Genomes (KEGG) enrichment analysis was carried out using gene families expanded in *C. trifoliata* relative to its ancestor (right panel). **(B)** Collinearity analysis of two *dGlyT* gene pairs involved in neohesperidoside (Neo) biosynthesis within the Rutaceae family. In *Citrus*-related species, *dGlcT-1* and *UGT79B203* (both utilizing UDP-Glc as the sugar donor) are present, whereas *1,2RhaT* and *UGT79B202* (both utilizing UDP-Rha and catalyzing Neo biosynthesis) are absent. In early-diverging citrus (*C. trifoliata*), dispersed duplication of *dGlcT-1* gave rise to *1,2RhaT*, which exclusively utilizes UDP-Rha. Similarly, tandem duplication of *UGT79B203* generated *UGT79B202*, which exhibits dual activity toward UDP-Glc and UDP-Rha. During this evolutionary phase, duplication and neofunctionalization of *dGlcT-1* and *UGT79B203* produced two rhamnosyltransferases, *1,2RhaT* and *UGT79B202*, thereby enabling Neo formation. The heatmap (right panel) shows Neo accumulation levels across different citrus and citrus-related species.

Total flavonoid content was higher in early-diverging and domesticated citrus species than in *Citrus*-related species (Supplemental Figure 1). Therefore, we conducted a comparative genomic analysis across the Rutaceae family. This analysis revealed that *C. trifoliata*, an early-diverging citrus species, has experienced gene family expansions relative to its ancestors, including expansions in families associated with flavonoid biosynthetic pathways. These genomic changes are consistent with the observed diversification of flavonoid metabolism (Figure 3A).

To investigate the evolutionary trajectories of the two pairs of paralogous *dGlyT* genes associated with Neo biosynthesis, we conducted a collinearity analysis across multiple taxa, including *Citrus*-related genera (*C*. *lansium*, *Luvunga scandens*, *A*. *buxifolia*), early-diverging citrus (*C. trifoliata*, *C*. *mangshanensis*, *C*. *ichangensis*), and domesticated citrus (*C. grandis*, *C*. *aurantium*, *C*. *sinensis*, *Citrus clementina*) (Figure 3B; Supplemental Data 4). This analysis showed that *dGlcT-1* and *UGT79B203*, which encode UDP-Glc-utilizing enzymes responsible for Sop biosynthesis, are present in the genomes of *Citrus*-related genera. In contrast, *1,2RhaT* and *UGT79B202*, which encode UDP-Rha-utilizing enzymes that catalyze Neo formation, first appeared in the genomes of early-diverging citrus species (Figure 3B). Duplication analysis further indicated that *1,2RhaT* arose through a dispersed duplication of *dGlcT-1*, whereas *UGT79B202* originated from a tandem duplication of *UGT79B203* (Figure 3B). These results suggest that gene duplication followed by neofunctionalization enabled a functional transition from 2″-*O*-glucosylation to 2″-*O*-rhamnosylation. This molecular innovation coincided with the absence of Neo in *Citrus*-related species and its emergence in early-diverging citrus lineages.

Collinearity analysis further revealed the absence of syntenic genes corresponding to *1,2RhaT* in mandarin and its derived species, *C*. *clementina* and *C. sinensis*. To confirm this absence, we analyzed whole-genome re-sequencing data from 135 accessions representing the three basic citrus species. The results showed that *1,2RhaT* is present in all pummelo accessions but absent in citron and most wild mandarin accessions (Supplemental Data 5). The loss of *1,2RhaT* in domesticated citrus may reflect adaptive responses to novel ecological or agronomic environments during domestication. Notably, *1,2RhaT* was detected in several cultivated mandarin accessions, likely due to introgression from pummelo. Consistently, heatmap analysis revealed a strong correlation between Neo accumulation and the presence of *1,2RhaT* across species. Although *C. sinensis* and *C. clementina* both retain *UGT79B202*, they exhibit markedly reduced Neo accumulation, underscoring the indispensable role of *1,2RhaT* in Neo biosynthesis.

In addition, collinearity analysis of other citrus *dGlyTs* revealed that *1,6RhaT* and *UGT91BK3* are present in *Citrus*-related genera and exhibit conserved synteny across multiple species (Supplemental Data 4), consistent with the detection of Rut in *C. lansium* and *A*. *buxifolia*.

### Catalytic mechanism, sugar-donor recognition, and specificity of Cm1,2RhaT

Given the crucial role of 1,2RhaT in Neo biosynthesis, we performed homology modeling and molecular docking analyses to identify key amino acid residues responsible for its catalytic activity and sugar-donor specificity (Supplemental Figure 10).

**Figure 4:**
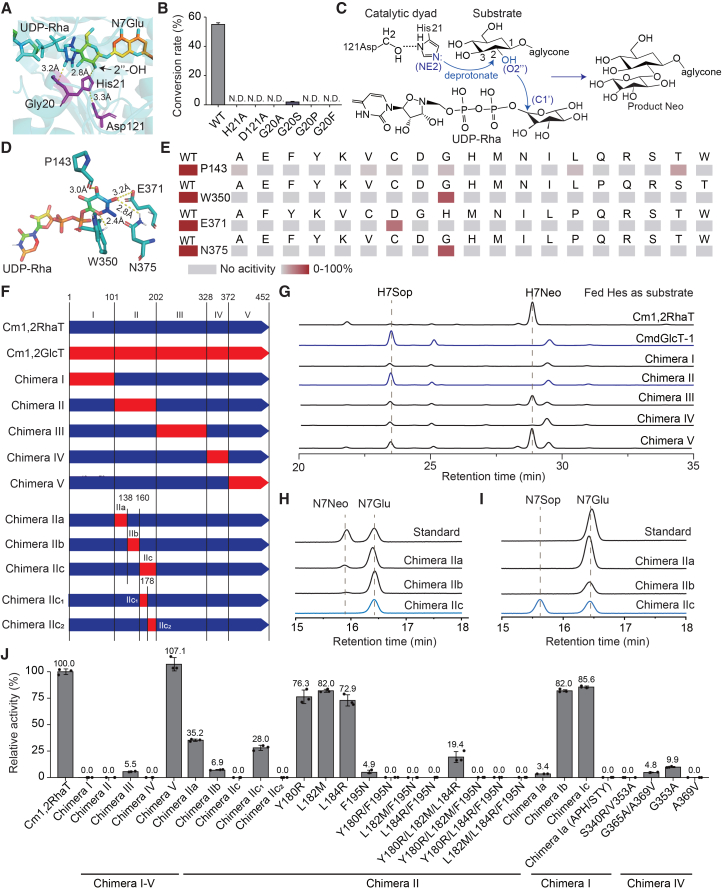
Catalytic mechanism, sugar-donor recognition, and specificity of Cm1,2RhaT. **(A)** Docking analysis of Cm1,2RhaT showing key catalytic residues involved in the catalytic mechanism. Yellow dashed lines indicate hydrogen bonds. **(B)** Enzyme activity assay of Cm1,2RhaT demonstrating that single-point mutations at key catalytic residues abolish the chemical conversion rate when N7Glu is used as the acceptor and UDP-Rha as the sugar donor. **(C)** Proposed S_N_2 catalytic mechanism of Cm1,2RhaT. His21 (assisted by Asp121) deprotonates the 2″-hydroxyl group of the glucose moiety attached to the aglycone, creating a nucleophile that initiates an S_N_2 attack on the C1′ carbon of UDP-Rha, resulting in neohesperidoside (Neo) formation. Key atoms involved in the reaction are shown in blue. **(D)** Positions of four residues that interact with the rhamnose moiety of UDP-Rha. **(E)** Heatmap showing functional screening of wild-type (WT) and mutant Cm1,2RhaT using N7Glu as the acceptor and UDP-Rha as the sugar donor. Mutations included replacement of each of the four interacting residues with the remaining 19 amino acids. **(F)** Schematic comparison of protein sequences of Cm1,2RhaT, CmdGlcT-1, and the chimeric variants generated in this study. Lines represent region boundaries, and numbers denote corresponding residue positions in the Cm1,2RhaT sequence. **(G)** Functional analysis of chimeric proteins (chimeras I–V) using hesperetin (Hes) as the substrate in Tobacco Bright Yellow 2 (BY2) suspension cells. **(Hand I)** Functional analysis of chimeric proteins (chimeras IIa, IIb, and IIc) using UDP-Rha **(H)** or UDP-Glc **(I)** as the sugar donor and N7Glu as the acceptor in the *E. coli* expression system. Blue lines represent HPLC elution profiles corresponding to dGlcT activity. **(J)** Relative rhamnosylation activity of chimeric and single or multiple mutant proteins expressed in *E. coli*. H7Neo, hesperetin 7-*O*-neohesperidoside; H7Sop, hesperetin 7-*O*-sophoroside; N7Glu was used as the acceptor. N7Glu, naringenin 7-*O*-glucoside; N7Neo, naringenin 7-*O*-neohesperidoside; N7Sop, naringenin 7-*O*-sophoroside. Error bars represent standard deviation from three biological replicates. The dashed line marks a reference peak used to distinguish compounds with similar retention times.

Structural modeling revealed a catalytic dyad composed of highly conserved His and Asp residues located near the active site. Docking analysis showed that His21 forms hydrogen bonds with both the 2-hydroxyl group of N7Glu and the side chain of Asp121 in Cm1,2RhaT (Figure 4A). Alanine substitution of either His21 or Asp121 completely abolished rhamnosylation activity, confirming the indispensable role of this catalytic dyad in Cm1,2RhaT function (Figure 4B). Additionally, Gly20 forms a hydrogen-bond with the substrate, and substitution at this position resulted in a complete loss of catalytic activity (Figure 4B), suggesting a critical role in stabilizing substrate binding and facilitating proper positioning of His21 within the active site. The catalytic reaction is mediated by the His–Asp dyad via a direct-displacement, S_N_2-like pathway (Figure 4C). Briefly, the 2-hydroxyl group (O2″) of the glucose moiety attached to the aglycone forms a hydrogen bond with the nitrogen (NE2) of His21. His21 is, in turn, hydrogen bonded to Asp121, creating the catalytic dyad. His21 acts as a general base to deprotonate O2″, generating a nucleophile that subsequently attacks the C1′ carbon of UDP-Rha, resulting in Neo formation.

In addition, residues Phe143, Glu371, Trp350, and Asn375 were found to interact with the rhamnose moiety of UDP-Rha (Figure 4D), suggesting potential roles in sugar-donor recognition or specificity. To evaluate their functional importance, saturation mutagenesis was performed at each position. Crude protein extracts from the resulting variants were assayed using N7Glu as the acceptor substrate in the presence of four sugar donors: UDP-Rha, UDP-Glc, UDP-Xyl, and UDP-Gal. Nearly all substitutions led to a substantial reduction or complete loss of rhamnosylation activity (Figure 4E). However, no activity toward alternative sugar donors was detected except for UDP-Rha, indicating that although Phe143, Glu371, Trp350, and Asn375 are essential for sugar-donor recognition, they do not independently determine sugar-donor specificity.

To further identify residues governing sugar-donor specificity, we conducted domain-swapping analyses between Cm1,2RhaT and CmdGlcT-1. A series of chimeric proteins was generated by replacing defined regions of Cm1,2RhaT with the corresponding segments from CmdGlcT-1. These regions were designated as regions I (residues 1–101), II (102–202), III (203–328), IV (329–372; PSPG box), and V (363–452) (Figure 4F; Supplemental Figure 11). Functional assays were conducted in Tobacco Bright Yellow 2 (BY2) suspension cells, which endogenously express enzymes capable of producing 7-*O*-glucosides. When hesperidin was used as the substrate, chimeras I, II, and IV completely lost rhamnosylation activity, whereas chimera III showed a marked reduction in activity. In contrast, chimera V retained activity comparable to that of wild-type Cm1,2RhaT. Notably, chimera II gained glucosylation activity while losing rhamnosylation activity, indicating that region II contains key residues governing sugar-donor specificity (Figure 4G). A similar pattern was observed when naringenin was used as the substrate (Supplemental Figure 12). To further refine this region, region II was subdivided into regions IIa, IIb, and IIc (Figure 4F). Functional assays showed that chimeras IIa and IIb retained weak rhamnosylation activity, whereas chimera IIc completely lost rhamnosylation activity while gaining glucosylation activity (Figures 4H and 4I). Structural modeling indicated that the sugar-donor binding pocket is primarily formed by α-helices within regions I, II, and IV, whereas region V is positioned outside the pocket (Supplemental Figure 13). Compared with Cm1,2RhaT, the binding pocket of chimera IIc was enlarged to a size similar to that of CmdGlcT-1 (Supplemental Figures 14A–14D). This expanded pocket more readily accommodates UDP-Glc, which is bulkier than UDP-Rha due to the presence of an additional hydroxyl group (Supplemental Figure 14E). These results suggest that region IIc plays a pivotal role in determining sugar-donor specificity, likely by modulating the size and geometry of the donor-binding pocket.

Further subdivision and site-directed mutagenesis of regions I, II, and IV were subsequently performed in *E. coli* (Figure 4J). Although none of the resulting mutants acquired glucosylation activity, the loss of rhamnosylation activity provided important insights into the functional relevance of these regions. Within region IIc, all four simultaneous substitutions in mutant chimera IIc_2_ completely abolished rhamnosylation function. Among these sites, multipoint mutations involving Phe195 abolished activity, whereas mutations at the other three positions retained rhamnosylation activity, indicating that Phe195 is the most critical residue within region IIc. Additionally, replacement of the Ala–Pro–His tripeptide (residues 23–25) in region I, as well as single or multiple mutations in region IV, resulted in substantial or complete loss of rhamnosylation function (Figure 4J). Structural analyses revealed that these key residues are located at positions distal to the ligand-binding site, beyond van der Waals contact distance (Supplemental Figure 15), suggesting that their effects are mediated through indirect structural modulation rather than direct substrate interactions.

### A distal residue corresponding to Phe195 modulates catalytic activity and specificity across functional citrus dGlyTs

**Figure 5:**
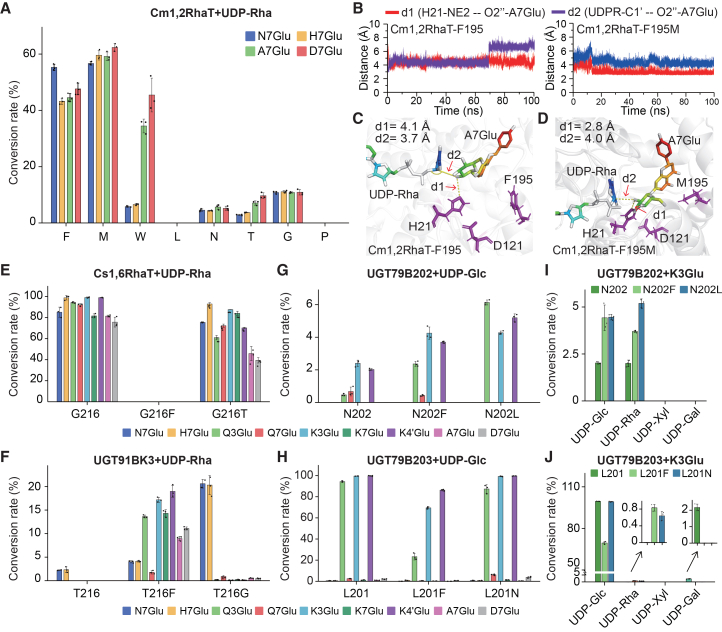
Functional analysis of mutations at the residue corresponding to F195 in different citrus dGlyTs. **(A)** Conversion rates of Cm1,2RhaT variants carrying mutations at residue F195 using UDP-Rha as the sugar donor and nine different flavonoids as acceptors. Residue F195 was substituted with eight different amino acids (F, W, M, L, N, T, G, and P), generating eight mutant proteins. **(B)** Distance variations of Cm1,2RhaT-F195 and Cm1,2RhaT-F195M during 100-ns molecular dynamics (MD) simulations. d1, distance between the nitrogen atom (NE2) of His21 and the 2-hydroxy (O2″) of A7Glu; d2, distance between the C1′ carbon (C1′) of UDP-Rha and the 2-hydroxy (O2″) of A7Glu. **(Cand D)** Representative catalytic conformations of Cm1,2RhaT-F195 **(C)** and Cm1,2RhaT-F195M **(D)** in MD simulations. Dashed lines indicate measured distances. **(E–H)** Conversion rates of mutants at residues corresponding to site 195 in Cs1,6RhaT **(E)**, UGT91BK3 **(F)**, UGT79B202 **(G)**, and UGT79B203 **(H)** using nine different flavonoids as substrates and the corresponding UDP-sugar as donor. **(Iand J)** Conversion rates of mutants at residues corresponding to site 195 in UGT79B202 **(I)** and UGT79B203 **(J)** using K3Glu as the acceptor and four different UDP-sugars as donors. Error bars represent standard deviations from three biological replicates. N7Glu, naringenin 7-*O*-glucoside; H7Glu, hesperetin 7-*O*-glucoside; Q3Glu, quercetin 3-*O*-glucoside; Q7Glu, quercetin 7-*O*-glucoside; K3Glu, kaempferol 3-*O*-glucoside; K7Glu, kaempferol 7-*O*-glucoside; K4′Glu, kaempferol 4′-*O*-glucoside; A7Glu, apigenin 7-*O*-glucoside; D7Glu, diosmetin 7-*O*-glucoside.

To further assess the functional importance of Phe195 in Cm1,2RhaT, this residue was substituted with several representative amino acids. Substitutions with non-hydrophobic residues resulted in a pronounced reduction or complete loss of catalytic activity, indicating that hydrophobicity at position 195 is essential for enzyme function (Figure 5A). Among hydrophobic substitutions, F195L completely abolished catalytic activity, F195W reduced activity toward flavanone substrates while retaining high activity toward flavone substrates, and F195M significantly enhanced catalytic activity across most tested substrates (Figure 5A; Supplemental Table 5). These results indicate that, beyond hydrophobicity, the size and chemical properties of the side chain at position 195 play a critical role in regulating the rhamnosylation activity and substrate preference of Cm1,2RhaT.

Because Phe195 and its variants are located at a distal site and do not directly interact with ligands (Supplemental Figure 16), we performed 100-ns unconstrained molecular dynamics (MD) simulations of Cm1,2RhaT-F195 and Cm1,2RhaT-F195M in complex with UDP-Rha and A7Glu to explore their mechanistic roles. Based on the proposed catalytic mechanism of Cm1,2RhaT (Figure 3C), two distances were analyzed to evaluate catalytic efficiency: d1, between the NE2 atom of catalytic His21 and the O2″ atom of A7Glu, and d2, between the C1′ carbon of UDP-Rha and the O2″ atom of A7Glu. The results showed that Cm1,2RhaT-F195M had a shorter d1 and a more stable d2 than Cm1,2RhaT-F195 (Figure 5B). Representative conformations from the MD simulations showed a reduction in d1 from 4.1 Å to 2.8 Å, together with a lower Molecular Mechanics/Poisson-Boltzmann Surface Area calculated binding free energy, which may reduce the energetic barrier for deprotonation and thereby enhance catalytic activity (Figures 5C and 5D; Supplemental Figure 17F). Additionally, root-mean-square deviations of both the protein and UDP-Rha were more stable in Cm1,2RhaT-F195M, and this variant formed more hydrogen bonds between the enzyme and its ligands (Supplemental Figures 17A–17E). Collectively, these findings suggest that residue 195 modulates enzymatic activity by reshaping the binding pocket and strengthening enzyme–ligand interactions.

To assess whether this site also plays a functional role in other citrus dGlyTs, we structurally aligned multiple citrus dGlyTs with Cm1,2RhaT to identify residues corresponding to Phe195 and mutated them to phenylalanine (Phe) (Supplemental Figure 18). We then introduced substitutions at the corresponding positions in Cs1,6RhaT and UGT91BK3, which catalyze β1–6 linkages but differ in substrate promiscuity, as well as in UGT79B202 and UGT79B203, which catalyze β1–2 linkages but differ in sugar-donor specificity. Nine substrates, including flavanones, flavonols, and flavones, were used to evaluate substrate promiscuity, and four sugar donors were used to examine sugar-donor specificity.

In Cs1,6RhaT, the G216F mutation completely abolished 1,6-rhamnosylation activity, whereas the G216T mutation caused a reduction in enzyme activity (Figure 5E; Supplemental Table 5). In UGT91BK3, the T216G mutation increased activity toward the flavanone substrates N7Glu and H7Glu by 9.24- and 8.68-fold, respectively, while maintaining weak activity toward other substrates. The T216F mutation significantly enhanced substrate promiscuity and shifted the preferred substrate from flavanone to flavonol (Figure 5F; Supplemental Table 5). In UGT79B202, the N202F and N202L mutations enhanced activity toward the optimal substrate K3Glu by 1.72- and 1.73-fold, respectively (Figure 5G; Supplemental Table 5). In contrast, in UGT79B203, the L201F mutation reduced activity, whereas the L201N mutation had little effect (Figure 5H; Supplemental Table 5). Sugar-donor assays showed that mutations in UGT79B202 did not alter sugar-donor specificity or preference (Figure 5I; Supplemental Table 5). However, mutations in UGT79B203 conferred weak rhamnosylation activity in addition to glucosylation function, indicating that this site contributes to the evolution of sugar-donor specificity in UGT79B203 (Figure 5J; Supplemental Table 5).

Docking and interaction analyses showed that residues corresponding to site 195 in these citrus dGlyTs do not directly interact with substrates (Supplemental Figures 19A–19D). Structural analyses of the mutant proteins revealed that the Cs1,6RhaT-G216F substitution introduced steric hindrance between F216 and the acceptor substrate, resulting in loss of activity. No obvious steric hindrance was observed in the other mutants, suggesting that their functional effects are mediated through indirect structural mechanisms similar to those observed for Cm1,2RhaT-F195M (Supplemental Figures 19E−19H).

### Potential roles of Neo accumulation in the adaptive evolution of citrus

Given its distinct evolutionary trajectory, Neo may possess biological functions that differ from those of Rut. To explore and compare the potential functional differences between these two disaccharide glycosides, we first analyzed their antioxidant activities, together with those of their corresponding aglycones and monoglucosides. The results showed that glycosylation generally reduced antioxidant capacity relative to aglycones, with no consistent trend in antioxidant reduction across different glycosylation types (Supplemental Figure 20A).

**Figure 6:**
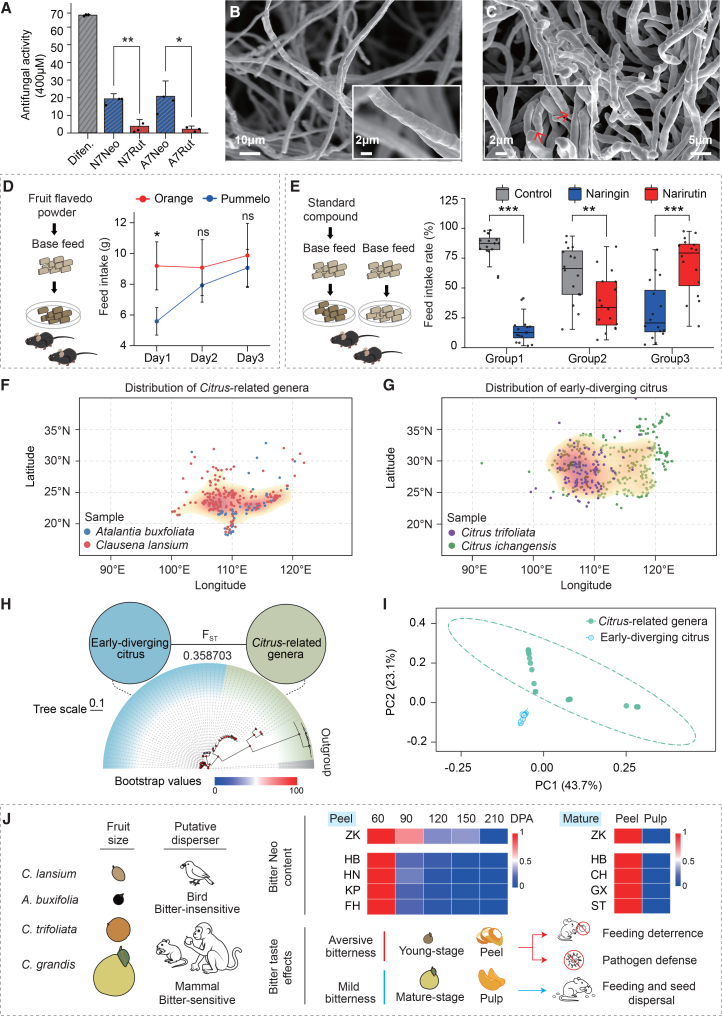
Biological functions of neohesperidosides and their potential roles in biotic defense and geographic distribution of *Citrus*. **(A)** Antifungal activities of representative neohesperidoside and rutinoside compounds against the fungal pathogen *Colletotrichum gloeosporioides*. Difenoconazole (Difen.) was used as a positive control. **(Band C)** SEM images of *Colletotrichum* hyphae exposed to the negative control (DMSO) **(B)** and N7Neo **(C)**. **(D)** Left, schematic illustration of the feed intake assay. Right, feed intake of adult C57 mice over 3 days (*n* = 3 cages per group, 2 mice per cage). **(E)** Left, schematic illustration of the feed intake rate assay. Right, feed intake of adult C57 mice recorded every 24 h for different treatment groups (*n* = 8 cages per group, 2 mice per cage). Group 1 received base feed and feed supplemented with naringin. Group 2 received base feed and feed supplemented with narirutin. Group 3 received feed supplemented with naringin and feed supplemented with narirutin. The final supplement concentration was 30 mg/g. N7Neo, naringenin 7-*O*-neohesperidoside; N7Rut, naringenin 7-*O*-rutinoside; A7Neo, apigenin 7-*O*-neohesperidoside; A7Rut, apigenin 7-*O*-rutinoside. Data are presented as mean ± SD. Statistical significance was determined using Student’s *t*-test. ns, not significant; ∗*p* < 0.05, ∗∗*p* < 0.01, ∗∗∗*p* < 0.001. **(Fand G)** Geographic distribution of *Citrus*-related genera (*Clausena lansium* and *A. buxifolia*) **(F)** and early-diverging citrus (*C. trifoliata* and *C. ichangensis*) **(G)**. **(H)** Phylogenetic and population divergence analysis. A maximum likelihood (ML) phylogeny was constructed with RAxML using 1000 bootstrap replicates. Two outgroup accessions were included to root the tree. Population divergence (F_ST_) among geographic groups was calculated in sliding windows of 50 kb with a 10 kb step size. **(I)** PCA analysis based on genome-wide SNPs. **(J)** Schematic model illustrating the effect of citrus bitter compounds on feeding behavior. Small-fruited species such as *C. lansium* and *A. buxifolia* are likely adapted for bird-mediated seed dispersal, which is insensitive to bitterness, whereas larger-fruited species such as *C. trifoliata* and *C. grandis* are adapted for mammalian dispersers that are sensitive to bitterness. Bitter neohesperidosides accumulate at high levels in young fruits and peels, potentially functioning in both feeding deterrence and pathogen defense, while reduced accumulation at maturity may facilitate fruit consumption and seed dispersal.

We next investigated their antifungal properties. Naringenin 7-*O*-neohesperidoside (N7Neo), the most abundant Neo compound in citrus, was first tested for antifungal activity against three common citrus fungal pathogens: *Colletotrichum gloeosporioides*, *Alternaria alternata*, and *Diaporthe citri*. These pathogens preferentially infect young and tender tissues, such as newly emerging shoots and young fruits. Preliminary screening revealed that N7Neo strongly inhibited the growth of *C. gloeosporioides*. We therefore compared the antifungal activities of two representative Neo compounds from pummelo with those of their corresponding Rut compounds against *Colletotrichum*. DMSO was used as a negative control, and difenoconazole, a broad-spectrum fungicide, served as a positive control. At a concentration of 400 μM, both Neo compounds exhibited significantly stronger antifungal activity than their Rut counterparts (Figure 6A; Supplemental Figure 20B). To further investigate the antifungal mechanism, we examined the ultrastructure of *Colletotrichum* hyphae treated with N7Neo or the negative control using scanning electron microscopy (SEM). Hyphae in the negative control displayed smooth and regular surfaces (Figure 6B), whereas hyphae exposed to 400 μM N7Neo appeared sunken and twisted (Figure 6C), indicating that N7Neo treatment disrupted cell wall and/or membrane integrity, thereby inhibiting fungal growth.

Because bitter compounds are often linked to feeding deterrence, we further examined the anti-feeding effects of Neo and Rut compounds in mice. A feed-intake assay was conducted by mixing fruit flavedo powder from pummelo (Neo-rich) or sweet orange (Rut-rich) into a base diet and monitoring consumption over 3 days. On the first day, mice fed pummelo powder exhibited significantly lower feed intake than those fed orange powder (Figure 6D), indicating an immediate aversive response. However, intake in the pummelo-fed group increased on the second and third days and no longer differed significantly from that of the orange-fed group (Figure 6D), likely reflecting hunger overriding the initial aversion.

To directly compare the effects of Neo and Rut, a two-bowl feeding assay was performed using base feed as a control and base feed supplemented with either N7Neo or naringenin 7-*O*-rutinoside (N7Rut) standards (Figure 6E). In both group 1 (N7Neo vs. base feed control) and group 2 (N7Rut vs. base feed control), intake of the supplemented feed was significantly lower than that of the base feed, with N7Neo showing a lower intake rate than N7Rut. Consistently, in group 3 (N7Neo vs. N7Rut), intake of the N7Neo-supplemented feed was significantly lower than that of the N7Rut-supplemented feed. Together, these results demonstrate that Neo exhibits a stronger anti-feeding effect than Rut in mice.

To investigate whether these biological activities contribute to broader environmental adaptation, we analyzed geographic records from 341 samples of *Citrus*-related genera (*A*. *buxifolia* and *C*. *lansium*) and 439 samples of early-diverging citrus species (*C. trifoliata* and *C*. *ichangensis*) (Supplemental Data 6). The results showed that the distributions of *C. trifoliata* and *C*. *ichangensis* are significantly broader than those of *A*. *buxifolia* and *C*. *lansium* (Figures 6F and 6G). To genetically contextualize this geographic pattern, we conducted a population genomic analysis of 40 accessions from early-diverging citrus (*C. trifoliata* and *C*. *ichangensis*) and 30 accessions from *Citrus*-related genera (*A. buxifolia*, *Murraya paniculata*, and *C. lansium*) (Supplemental Data 7). Based on 7 158 420 high-quality SNPs, maximum likelihood (ML) phylogenetic analysis resolved two well-separated lineages corresponding to *Citrus*-related genera and early-diverging citrus. This separation is further supported by principal component analysis (PCA) clustering and high pairwise F_ST_ values (Figures 6H and 6I). Importantly, this pronounced genomic divergence mirrors contrasting metabolic phenotypes: all early-diverging citrus accessions uniformly accumulate Neo, whereas *Citrus*-related genera do not (Figure 1B). Together with the demonstrated antifungal and anti-feeding activities of Neo (Figures 6A–6E), these genomic and chemotypic differences support the conclusion that the emergence of Neo biosynthesis likely contributes to the enhanced ecological resilience of early citrus lineages and facilitates their broader geographic expansion.

## Discussion

The vast chemical diversity of plant metabolites is largely driven by complex modifications of core scaffolds. Recent advances, such as widely targeted metabolite modificomics, have substantially enhanced the detection and identification of modified metabolites (Yang et al., 2024). In this study, we conducted a systematic profiling of glycosylated metabolites in citrus using widely targeted metabolomic analysis. This approach refined our understanding of glycosylation diversity and revealed that the emergence of bitter Neo is closely associated with a functional shift from 2″-*O*-glucosylation to 2″-*O*-rhamnosylation.

Many studies have demonstrated that changes in one or a few amino acids, particularly at distal sites, can lead to pronounced alterations in enzyme function (Moghe and Last, 2015; Lopez-Nieves et al., 2022). For instance, only three amino acid substitutions in KAI2 conferred responsiveness to strigolactones, and a single coding SNP in *GmPM30* enhanced soybean salinity tolerance and yield (Arellano-Saab et al., 2021; Huang et al., 2025). Here, we identified a structurally conserved distal residue, Phe195 in Cm1,2RhaT and the corresponding Leu201 in UGT79B203, that modulates sugar-donor specificity and enables Neo biosynthesis. Structural comparisons across diverse plant dGlyTs revealed high variability at this position, in contrast to the conserved active-site residues essential for catalysis (Supplemental Figure 21). Unlike active-site residues, distal residues do not directly contact ligands and are therefore more prone to adaptive mutations that drive functional divergence (Soskine and Tawfik, 2010; Zong et al., 2019; Gu et al., 2023). MD simulations further showed that Phe195 reshapes the substrate-binding pocket of Cm1,2RhaT, thereby modulating enzyme activity. This mechanism is consistent with findings in UGT76G1, in which multiple distal mutations (T284S/M88L/L200A) remodel the hydrophobic pocket to accommodate the larger substrate Reb D (Guo et al., 2022). Additional studies have shown that distal residues can influence enzyme function indirectly through residue–residue interaction networks or by altering the geometry and accessibility of the substrate channel (Tan et al., 2022). Together, these findings provide a molecular and structural basis for understanding how amino acid variation drives enzyme functional evolution.

Gene duplication is a major driver of metabolic innovation. Although most duplicated genes are eventually lost, some are selectively retained following acquisition of beneficial new functions through neofunctionalization (Huang et al., 2018; Peng et al., 2024). Our findings indicate that the Neo biosynthetic genes (*Cm1,2RhaT* and *UGT79B202*) originated from duplication of ancestral *dGlcT* genes and were retained because of the strong antifungal and anti-feeding activities conferred by Neo (Figures 6A–6E). Based on these findings, we propose a speculative model of a bitterness-mediated defense strategy in citrus (Figure 6J). *Citrus*-related species such as *C*. *lansium* and *A*. *buxifolia*, produce relatively small fruits, for which seed dispersal may rely on bitter-insensitive birds. In contrast, species such as *C. trifoliata* and *C. grandis* produce larger fruits that are more likely dispersed by bitter-sensitive mammals. Neo accumulation was observed in *C. trifoliata* (ZK) and in four *C. grandis* accessions (HB, HN, KP, and FH) across multiple developmental stages and tissues. Neo levels were highest in young fruits and gradually declined during fruit maturation, with mature fruits showing significantly higher Neo accumulation in the peel than in pulp tissues. This spatiotemporal pattern suggests a dual function for Neo: providing feeding deterrence and pathogen defense during early fruit development, while decreasing in edible tissues (pulp) at ripening to facilitate fruit consumption and seed dispersal. Such dynamic regulation aligns with general models of the functions of secondary metabolites in fruit, which balance defense and reproduction (Herrera, 1982; Tewksbury et al., 2008). For instance, capsaicinoid levels decline during fruit ripening in chili (*Capsicum chacoense*) (Tewksbury et al., 2008), and glucosinolates show tissue- and stage-specific accumulation patterns in *Arabidopsis thaliana* (Brown et al., 2003). Importantly, we also observed that two major limonoids in citrus, limonin and nomilin, which are responsible for delayed bitterness, accumulate predominantly in seeds, highlighting a potential division of labor between limonoids and Neo in citrus defense (De La Pena et al., 2023). In other tissues, limonoids show spatial distribution patterns similar to those of Neo, with high abundance in the peel and segment membranes (Supplemental Data 8), further supporting the proposed defense model. Together, these findings illustrate how the spatial and temporal regulation of bitterness contributes to adaptive trade-offs between defense and seed dispersal during citrus evolution.

The emergence of bitter Neo likely plays a significant role in the dissemination and ecological expansion of *Citrus*. Our findings show that the non-bitter Rut is present in both citrus and *Citrus*-related species, whereas Neo emerges specifically in early-diverging citrus lineages. *Citrus*-related genera such as *C. lansium* and *A. buxifolia* are distributed in tropical and subtropical regions (Figure 6F), where herbivory pressure is primarily insect-driven (Becerra, 2015). In contrast, early-diverging citrus species, including *C. trifoliata* and *C. grandis*, inhabit subtropical to temperate regions (Figure 6G), where bitter-sensitive mammals impose stronger selective pressure (Becerra, 2015). Notably, diversification of early-diverging citrus occurred during the late Miocene epoch, a period characterized by extensive mammalian radiation (Bininda-Emonds et al., 2007). Together, these observations suggest that the emergence of Neo represents an adaptive metabolic innovation in response to shifting herbivore pressure during this ecological transition. While Wu et al. (2018) emphasized climatic factors as primary drivers of citrus expansion, our results highlight the importance of biotic interactions, including pathogens and herbivores, as additional selective forces shaping citrus evolution.

Beyond natural selection, domestication has profoundly influenced the distribution and abundance of Neo in citrus. Previous studies demonstrated that artificial selection against bitterness led to the loss of the *1,2RhaT* gene in modern sweet orange cultivars (Chen et al., 2019). In this study, we further confirmed that multiple single-amino-acid substitutions in Cm1,2RhaT result in loss of enzyme function (Figure 5). Such mutations are present in modern commercial pummelo cultivars, including Guanximi and Shatian, which exhibit significantly lower Neo content (Supplemental Figure 22). In contrast, Zhengmao and Jiaxiyang pummelos—traditional cultivars from Huazhou in Guangdong Province used in medicinal preparations—retain a functional *1,2RhaT* gene and accumulate high levels of Neo, suggesting that Neo may have been selectively preserved for its medicinal value. A comparable pattern has been reported in cucumber, where selection on the Bt transcription factor during domestication led to the loss of bitterness (Shang et al., 2014). As cultivated plants experience reduced environmental pressures, defense strategies and metabolite profiles are often reshaped accordingly (Zust and Agrawal, 2017). The evolutionary gain and subsequent loss of Neo biosynthesis in citrus exemplify the complex interplay between natural and artificial selection in shaping plant specialized metabolism and phenotypic diversity.

In conclusion, our study demonstrates that two pairs of paralogous *dGlyTs* contribute to Neo biosynthesis in citrus. In early-diverging citrus species, gene duplication followed by neofunctionalization drove the evolution of rhamnosyltransferase activity required for Neo production. We identify a critical amino acid residue, corresponding to Phe195 in Cm1,2RhaT and Leu201 in UGT79B203, as a key determinant of this neofunctionalization and demonstrate that Neo possesses stronger antifungal and anti-herbivory activities than Rut. These traits likely facilitated the ecological expansion of early-diverging citrus beyond their *Citrus*-related ancestors. Overall, this study provides new insights into the evolutionary trajectory of bitter Neo in *Citrus* and underscores the adaptive roles of specialized metabolites in plant–environment interactions, with potential implications for citrus flavor improvement and functional food development.

## Methods

### Plant materials

Fruits from 38 citrus accessions were used in this study, covering *Citrus*-related genera (*C. lansium* and *A. buxifolia*), early-diverging citrus (*C. trifoliata*, *C. ichangensis*, and *C. mangshanensis*), and domesticated citrus (*C. grandis*, *C. reticulata*, *C. medica*, *C. aurantium*, *C. sinensis*, and *C. limon*) (Supplemental Table 1). For each accession, 15–18 fruits were randomly harvested from at least three healthy trees at the commercial harvest stage, approximately 230 days post anthesis. Fruits were randomly divided into three biological replicates. Flavedo tissues were isolated, immediately frozen in liquid nitrogen, and stored at −80°C until analysis. Collection locations and detailed accession information are provided in Supplemental Table 1.

### Commercially available chemical standards

Chemical standards were purchased from Shanghai Yuanye Bio-Technology (Shanghai, China). These included flavanones (naringenin 7-*O*-glucoside, hesperetin 7-*O*-glucoside), flavonols (quercetin 3-*O*-glucoside, quercetin 7-*O*-glucoside, kaempferol 3-*O*-glucoside, kaempferol 7-*O*-glucoside, kaempferol 4′-*O*-glucoside), flavones (apigenin 7-*O*-glucoside, diosmetin 7-*O*-glucoside), three sugar donors (UDP-glucose, UDP-xylose, and UDP-galactose), and flavonoid disaccharide glycosides (naringenin 7-*O*-neohesperidoside, naringenin 7-*O*-rutinoside, hesperetin 7-*O*-neohesperidoside, hesperetin 7-*O*-rutinoside, quercetin 3-*O*-neohesperidoside, quercetin 3-*O*-sophoroside, kaempferol 3-*O*-neohesperidoside, and kaempferol 3-*O*-sophoroside). UDP-rhamnose was purchased from Angfei Biological Technology (Guangdong, China). Acyclovir and roxithromycin were obtained from Sigma-Aldrich (USA). Detailed information on all chemicalstandards used in this study is provided in Supplemental Table 6.

### Metabolic profiling

Sample preparation for metabolic analysis was modified from a previously described method (Peng et al., 2024). All samples were freeze-dried using a lyophilizer (Heto Lylab 3000) and ground into a fine powder. Powdered samples (50 ± 5 mg) were extracted with 0.5 ml of 70% methanol containing 0.4% internal standards (acyclovir and roxithromycin, 0.1 ppm each). After ultrasonic treatment at 4°C for 20 min, samples were centrifuged at 12 000 rpm for 30 min at 4°C. The supernatant was filtered through a 0.22-μm membrane and analyzed by high-performance liquid chromatography (HPLC) and liquid chromatography–mass spectrometry (Ultimate 3000 Nano and Thermo Q Exactive Plus Orbitrap, Thermo Scientific, Waltham, MA, USA) as described by Chen et al. (2013, 2020b). Metabolites were identified by comparison with commercial standards, in-house metabolite databases (Chen et al., 2013, 2020b), and the public mzCloud database (https://www.mzcloud.org/). Relative quantification was performed using scheduled multiple reaction monitoring on a triple quadrupole–linear ion trap mass spectrometer (QTRAP 6500^+^, AB SCIEX, Framingham, MA, USA). Data acquisition and processing were conducted using Analyst 1.5 software. Each sample was analyzed using three biological replicates.

### Gene identification and phylogenetic analysis

Citrus UGTs were identified using the Simple HMM Search program in TBtools based on the UGT hidden Markov model profile PF00201 obtained from the Pfam database (http://pfam.xfam.org/) (Chen et al., 2020a). Predicted *UGT*s containing ORFs encoding 340–680 amino acids were selected for phylogenetic analysis following the criteria of Wilson and Tian (2019). Protein sequences of citrus UGTs and 37 previously characterized UGTs representing different phylogenetic groups were aligned using multiple sequence comparison by log-expectation in MEGA X. Phylogenies were inferred by neighbor joining with 3000 bootstrap replicates. Information on the known UGTs used for phylogenetic analysis is provided in Supplemental Table 2. Similarly, for phylogenetic analysis of plant dGlyTs, functional citrus dGlyTs and 17 characterized dGlyTs from other plant species were aligned using multiple sequence comparison by log-expectation, and phylogenies were inferred by neighbor joining with 3000 bootstrap replicates. Information on the dGlyTs included in this analysis is provided in Supplemental Table 3.

### Protein expression and purification

Recombinant proteins corresponding to candidate *UGTs* and their mutant variants were produced in *E. coli*. Protein expression and purification followed previously described procedures (Li et al., 2022). Briefly, the relevant coding sequences were fused to a maltose-binding protein tag in the pMAL-c2x expression vector and subsequently transformed into *E. coli* BL21(DE3) cells. Protein expression was induced with isopropyl β-D-1-thiogalactopyranoside (IPTG) to a final concentration of 0.5 mM at 16°C for 16 h. Cells were harvested and lysed by sonication at 4°C in phosphate-buffered saline (pH 7.4) containing 400 mM NaCl. Target proteins were purified using a dextrin beads 6FF affinity column with a wash buffer (20 mM Tris–HCl, 20 mM NaCl, 1 mM EDTA, and 1 mM DTT, pH 7.4), followed by elution buffer (20 mM Tris–HCl, 1 mM EDTA, 10 mM maltose, and 1 mM DTT, pH 7.4).

### Enzyme activity assay and kinetics

Enzyme activity assays were performed in a total reaction volume of 100 μl containing 15 μg of purified protein, 0.1 mM flavonoid substrate, 0.5 mM UDP-sugar, and 0.25 mM MgCl_2_ in phosphate-buffered saline (50 mM, pH 7.4). Reactions were incubated for 0.5 h at 35 °C and terminated by adding 100 μl of ice-cold methanol prior to further analysis.

For crude enzyme extraction used in saturation mutagenesis experiments, bacterial cultures were established by inoculating 1 ml of Luria–Bertani liquid medium with 10 μl of overnight-activated cells. Cultures were incubated in a 24-well plate for 2.5 h at 37°C and then induced with IPTG to a final concentration of 0.5 mM at 16°C for 16 h. Cells were harvested and lysed in phosphate-buffered saline (pH 7.4) containing 750 mg/l lysozyme and 10 mg/l benzonase for 1 h at 37°C. Crude protein extracts were obtained from the supernatant following centrifugation. Crude enzyme assays were performed in a total volume of 100 μl containing 50 μl of supernatant, 0.1 mM flavonoid substrates, 0.5 mM UDP-sugar, and 0.25 mM MgCl_2_ in phosphate-buffered saline (50 mM, pH 7.4) for 10 h at 35°C.

To determine the kinetic parameters of UGT79B202 toward UDP-Glc and UDP-Rha, enzyme assays were carried out using kaempferol 3-*O*-glucoside (0.1 mM) and varying concentrations of UDP-Glc or UDP-Rha (0.01–0.9 mM). Reactions contained 50 ng of purified recombinant UGT79B202 in a total volume of 100 μl and were incubated at 40°C for 120 min. Kinetic parameters were calculated using the Michaelis–Menten model (OriginPro 2021).

### Genome evolution analysis

MCscan (Python version) (Wang et al., 2012) was used to identify synteny among the genomes of *Vitis vinifera*, *C. lansium*, *L. scandens*, *A. buxifolia*, *C. trifoliata*, *C. mangshanensis*, *C. ichangensis*, *C. medica*, *C. limon*, *C. grandis*, *C. aurantium*, *C. sinensis*, and *C. clementina*. Reference genome sequences of citrus and citrus-related genera were downloaded from the Citrus Pan-genome to Breeding Database (http://citrus.hzau.edu.cn/index.php).

### Generation of mutant gene sequences

DNA sequences corresponding to chimera I–V, Ia–c, IIa–c, IIc_1_, and IIc_2_ were synthesized by Beijing Tsingke Biotech. Single-point and multipoint mutations were generated using the Mut Express II Fast Mutagenesis Kit V2 (https://www.vazyme.com/product/79.html). All generated sequences were verified by DNA sequencing at Beijing Tsingke Biotech. Primers used for mutagenesis experiments are listed in Supplemental Data 9.

### Identification and quantification of flavonoids

Mass spectrometry data were acquired using a 1200 Series Rapid Resolution Ultra Performance Liquid Chromatography system coupled to a 1260 Infinity diode array detector and a 6520 Accurate-Mass Quadrupole Time-of-Flight (Q-TOF) MS system (Agilent Technologies, CA, USA). Ultrapure water and acetonitrile containing 0.04% (v/v) formic acid were used as mobile phases A and B, respectively. Flavonoid compounds were identified by comparing retention times, UV spectra, mass spectra, and ion fragmentation patterns with those of commercial chemical standards. Compounds lacking authentic standards were predicted based on molecular weight and characteristic ion fragments.

Flavonoid compounds were quantified using an HPLC system with the following conditions: chromatographic column (Accucore C18, 150 × 2.1 mm; Thermo Scientific, Waltham, MA, USA); mobile phase (A: ultrapure water containing 0.15% [v/v] formic acid; B: acetonitrile containing 0.15% [v/v] formic acid); gradient elution system (0 min, 10% B;10 min, 22% B; 20 min, 23% B; 25 min, 75% B; 27 min, 75% B; 30 min, 10% B; flow rate: 0.35 ml/min). Flavonoid substrates and their glycosides were quantified according to Yuan et al. (2024). Conversion rates were calculated as percentages based on peak areas of glycosylated products and corresponding sugar acceptors in HPLC chromatograms. Each reaction was performed with three independent experimental replicates.

### Biotransformation assay in tobacco bright yellow 2 (BY2) cells

Transgenic BY2 cells expressing Cm1,2RhaT, CmdGlcT-1, or chimeras I–V were generated as previously described (Chen et al. 2019). Target genes were cloned into the Ph7WG2D vector harboring GFP and introduced into *Agrobacterium tumefaciens* GV3101. Positive transformants were confirmed by fluorescence detection. For biotransformation assays, substrate-feeding experiments were performed according to the protocol described by Frydman et al. (2013). Briefly, BY2 cells were cultured in Erlenmeyer flasks for 1 week at 28°C with shaking at 140 rpm. Then, 1 g of calli was transferred to 20 ml of Murashige and Skoog liquid medium and incubated under the same conditions for 4 days. Substrates were then added, and cultures were incubated for an additional 2 days prior to cell harvest and flavonoid extraction.

### Molecular docking

Protein structure models were generated using AlphaFold3 (https://golgi.sandbox.google.com/). 3D structures of UDP-Rha and UDP-Glc, naringenin 7-*O*-glucoside, hesperetin 7-*O*-glucoside, apigenin 7-*O*-glucoside, and kaempferol 3-*O*-glucoside were extracted from the PubChem compound database (https://pubchem.ncbi.nlm.nih.gov/). Molecular structures of the substrates were optimized using the B97-3c composite method implemented in ORCA (v5.0.3) (Neese, 2022). Docking calculations were performed using AutoDock Vina to obtain enzyme–substrate complexes (Trott and Olson, 2010). Structural analyses and visualization were conducted using PyMOL for educational use (v2.4.1).

### Molecular dynamics (MD) simulations

MD simulations were conducted using GROMACS 2020.6. The Amber14SB force field was applied for protein simulations. Parameters for UDP-Rha and A7Glu (apigenin 7-*O*-glucoside) were generated using Sobtop_1.0 (dev3) (http://sobereva.com/soft/Sobtop) and Multiwfn (Lu and Chen, 2011; Lu, 2024) applying the GAFF force field. To derive RESP2 charges for parameterization, single-point energy calculations were performed at the B3LYP-D3/def2-TZVP level of theory based on wavefunctions obtained from structure optimization using ORCA. The system was placed in a cubic simulation box with a distance of 10 Å between the protein surface and the box boundary. Solvation was carried out using the TIP3P water model under periodic boundary conditions, and sodium ions were added to neutralize the system. Energy minimization consisted of 2500 steps of the steepest descent algorithm followed by 2500 steps of the conjugate gradient method, with a total force threshold of 100 kJ mol^−1^nm^−1^ to reduce atomic collisions and ensure that the system reached the minimum energy state. The system was pre-equilibrated under NPT conditions at 1.0 bar and a temperature of 298.15 K. A 100-ns MD simulation employing a 2-fs integration time step was subsequently conducted. Visual MD and QtGrace were used to analyze the results.

### Antioxidant assay

Total antioxidant capacity was determined using the 2,2′-azinobis(3-ethylbenzothiazoline-6-sulfonic acid) (ABTS) and 2,2-diphenyl-1-picrylhydrazyl (DPPH) radical scavenging assay, as well as the ferric ion reducing antioxidant power (FRAP) assay. Both ABTS and FRAP kits were purchased from Nanjing Jiancheng Bioengineering Institute, while the DPPH kit was purchased from Shanghailianzu Biotechnology.All assays were performed according to the manufacturer’s instructions.

### Antifungal assay and ultrastructure analysis

Strains of *C. gloeosporioides*, *A. alternata*, and *D. citri* were kindly provided by Professor Hongye Li from Zhejiang University. Fungal strains were activated on potato dextrose agar medium at 28°C. For antifungal assays, mycelial plugs excised from the margins of 3-day-old colonies were transferred to potato dextrose agar plates supplemented with 400 μM of the tested antifungal compounds (N7Neo, N7Rut, A7Neo, A7Rut). Potato dextrose agar plates containing an equal concentration of difenoconazole served as positive controls, while plates with an equal volume of DMSO were used as negative controls. After incubation for 72 h at 28°C, colony diameters were measured. Each experiment was performed with three biological replicates.

For SEM analyses, hyphal samples treated with N7Neo (400 μM) or DMSO (control) were collected and fixed overnight in 2.5% glutaraldehyde at 4°C. Samples were then examined using a scanning electron microscope (Hitachi SU-8010, Tokyo, Japan).

### Anti-feeding assay

A feed intake assay was conducted using 12 male C57 mice. Following a pre-experiment to record baseline daily food intake, mice were housed in six cages and divided into two groups based on intake. Base feed was ground and mixed with pummelo or orange albedo powder at a final concentration of 15 mg/g. The mixtures were reshaped into pellets and air-dried for 24 h. Food intake for each cage was recorded every 24 h for 3 days.

A two-bowl feeding preference assay was conducted using 16 male C57 mice. After a pre-experiment to record baseline intake, mice were housed in eight cages. Base feed was ground and mixed with standard compounds to a final concentration of 30 mg/g, reshaped into pellets, and air-dried for 24 h. Control feed was prepared in the same manner without additives. Three treatment groups were established: group 1 received control base feed and N7Neo-supplemented feed, group 2 received control base feed and N7Rut-supplemented feed, and group 3 received N7Neo- and N7Rut-supplemented feeds. Feed intake was recorded every 24 h for 2 days, and intake rates were calculated to assess feeding preference.

All animal procedures were approved by the Institutional Animal Care and Use Committee of Huazhong Agricultural University (HZAUMO-2025-0215).

### Geographical distribution

Distribution records for 341 samples of *Citrus*-related genera species (*A*. *buxifolia* and *C*. *lansium*) and 439 samples of early-diverging citrus (*C. trifoliata* and *C. ichangensis*) were collected from the Chinese Virtual Herbarium (https://www.cvh.ac.cn/) and the National Specimen Information Infrastructure (http://www.nsii.org.cn/node/79/cvh/12/f95/4850757). Coordinates and associated metadata are provided in Supplemental Data 2. Geographic distribution maps were generated using Python. Kernel density estimation was applied to records within the geographic range of 15°–40°N latitude and 85°−130°E longitude. To emphasize areas of high specimen density, only the upper 50th percentile of kernel density values was visualized.

### SNP calling and population genetic analysis

A total of 40 accessions of early-diverging citrus (*C. trifoliata* and *C. ichangensis*) and 30 accessions of *Citrus*-related genera (*A. buxifolia*, *M. paniculata*, and *C. lansium*), together with two outgroup accessions, were used for population genomic analyses (Supplemental Data 6). Following read mapping, variant calling, quality filtering, and linkage disequilibrium pruning, a high-confidence dataset of 7 158 420 SNPs was obtained for downstream analyses. ML phylogenetic inference was performed using RAxML (v7.7.8) software (Stamatakis, 2006) with 1000 bootstrap replicates. PCA was conducted using PLINK2 (Purcell et al., 2007), and pairwise genetic differentiation (F_ST_) was calculated using VCFtools v.0.1.16 (Danecek et al., 2011) with sliding windows of 50 kb and a 10-kb step size.

### Statistical analysis

All experiments were performed with at least three biological replicates. Graphs were generated using Origin 2021 Learning Edition (Microcal Software, Northampton, MA, USA). Statistical significance was assessed using one-way ANOVA followed by Tukey’s multiple range test or by Student’s *t*-test in SPSS (SPSS, Chicago, IL). Structural formulas were drawn using ChemBioDraw Ultra 12.0 (PerkinElmer Informatics, Waltham, MA, USA).

## Funding

This work was supported by the 10.13039/501100012166National Key Research and Development Program of China (grant no. 2023YFD2300600 to J.X. and J.C.), the 10.13039/501100001809National Natural Science Foundation of China (grant no. 32402481 to Z.Y.), and the 10.13039/501100001809National Natural Science Foundation of China (grant no. 32272685 to J.C.).

## Acknowledgments

The authors would like to thank Dongqin Li (Huazhong Agricultural University, China) for assistance with QTRAP mass spectrometry analyses. Patent applications related to the genes *UGT79B202*, *UGT79B203*, and *UGT91BK3* are currently in preparation.

## Author contributions

J.X. and J.C. conceived the project and overall strategy. G.L. designed the experiments and analyzed the data. G.L., H. Zhou, and Y.L. performed metabolite data analysis. H.W. assisted with bioinformatics analyses. G.L. conducted molecular docking and MD analyses. H.Zhang., Z.Y., Z.L., Z.H., Q.C., and G.C. helped with biochemical experiments. G. L. wrote the manuscript with contributions from J.-L.Y., J.X., and J.C. All authors discussed the results and approved the final version of the manuscript.

## Supplemental information

Supplemental Data. Supplemental Data 1–9

Document S2. Article plus supplemental information
