# Supplementary material for: Evolutionary origin of neohesperidoside, a bitter metabolite, and its potential role in biotic defense and citrus dissemination
Source: Plant Commun. 2026 Jan 5;7(4):101697. doi: 10.1016/j.xplc.2026.101697 (PMC13084081; doi:10.1016/j.xplc.2026.101697)
Supplement: Document S1. Supplemental Figures 1–22 and Supplemental Tables 1–6 [file mmc1.pdf]

Plant Communications, Volume *7*

Supplemental information

Evolutionary origin of neohesperidoside, a bitter metabolite, and its potential role in biotic defense and citrus dissemination

Gu Li, Huan Wen, Hanxin Zhou, Yuan Liu, Ziyu Yuan, Huixian Zhang, Zhehui Hu, Zeyang Liu, Huili Ma, Qi Chen, Guixiang Chen, Jia-Long Yao, Juan Xu, and Jiajing Chen

Supporting Information for

Emergence of the Bitter Metabolite Neohesperidoside and Its Potential Role in Biotic Defense and Citrus Dissemination

Gu Li [a,b,c], Huan Wen [a], Hanxin Zhou [a], Yuan Liu [a], Ziyuyuan [a], Huixian Zhang [a], Zhehui Hu [a], Zeyang Liu [a], Huili Ma [a], Qi Chen [a],Guixiang Chen [a], Jia-Long Yao [b], Juan Xu [a,c,d*], Jiajing Chen [a,c,d*]

[a] National Key Laboratory for Germplasm Innovation & Utilization of Horticultural Crops, College of Horticulture and Forestry Sciences, Huazhong Agricultural University, Wuhan, 430070, P.R. China. Hainan Research Institute of Huazhong Agricultural University, Sanya 572025, P.R. China.

[b] New Zealand Institute for Bioeconomy Science Limited, Private Bag 92169, Auckland 1142, New Zealand

[c] Hubei Hongshan Laboratory, Wuhan, 430070, P.R. China.

[d] Sensory Evaluation and Quality Analysis Centre of Horticultural Products, Huazhong Agricultural University, Wuhan 430070, China

*Correspondence: chenjiajing@mail.hzau.edu.cn (Jiajing Chen), xujuan@mail.hzau.edu.cn (Juan Xu)

This PDF file includes:

Supplemental Figures 1 to 22

Supplemental Tables 1 to 6

Other supporting materials for this manuscript include the following:

Supplemental Data 1. Summary of flavonoid compounds detected in mature fruit flavedo of thirty-five citrus accessions.

Supplemental Data 2. Concentrations (mg/g, DW) of the major Neo and Rut in citrus germplasms analyzed in this study.

Supplemental Data 3. Amplification analysis statistics of group A genes in *C. grands* (L.) Osbeck.cv. 'Wanbaiyou'.

Supplemental Data 4. Micro-synteny relationships among genes from species in *Citrus*-related genera, early-diverging citrus and other species.

Supplemental Data 5. Analysis of gene presence and absence for 135 sequenced genomes from citrus accessions and relatives.

Supplemental Data 6. Geographical occurrences of 341 *Citrus*-related genera and 439 early-diverging citrus samples.

Supplemental Data 7. Whole-genome resequencing metadata for 70 accessions used in population genomic analysis.

Supplemental Data 8. Limonoids content in different tissues of fifty-five citrus accessions.

Supplemental Data 9. Primers used in this study.

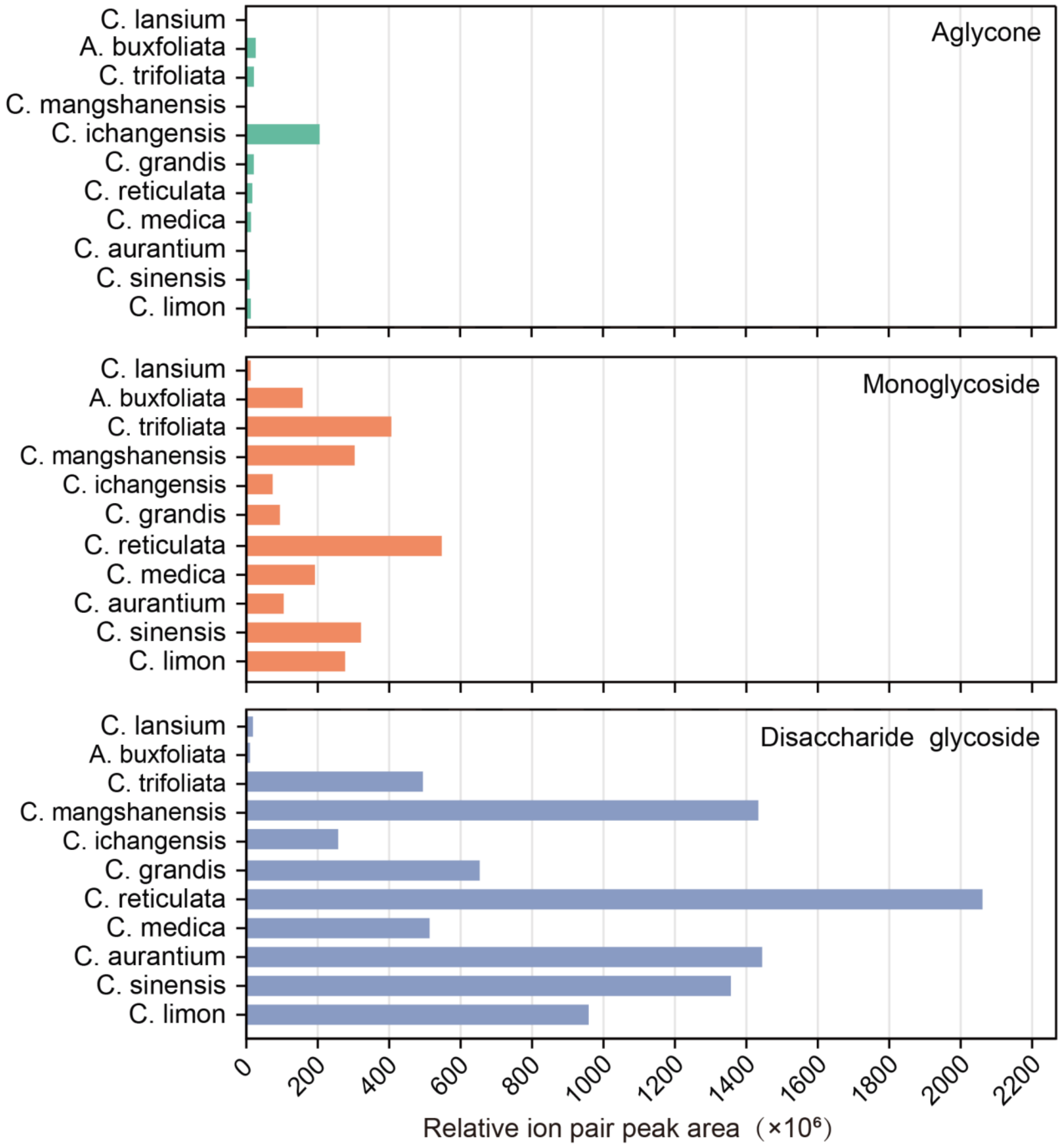

Supplemental Figure 1. Accumulation of flavonoid compounds in the fruit flavedo of various citrus species. Flavonoid metabolites were classified into aglycones, monoglycosides, and disaccharide glycosides. Each bar represents the summed peak areas of all detected compounds within each class in each species, as determined by LC-MS/MS. Colors represent different compound categories. Detailed compound information is listed in Dataset 1.

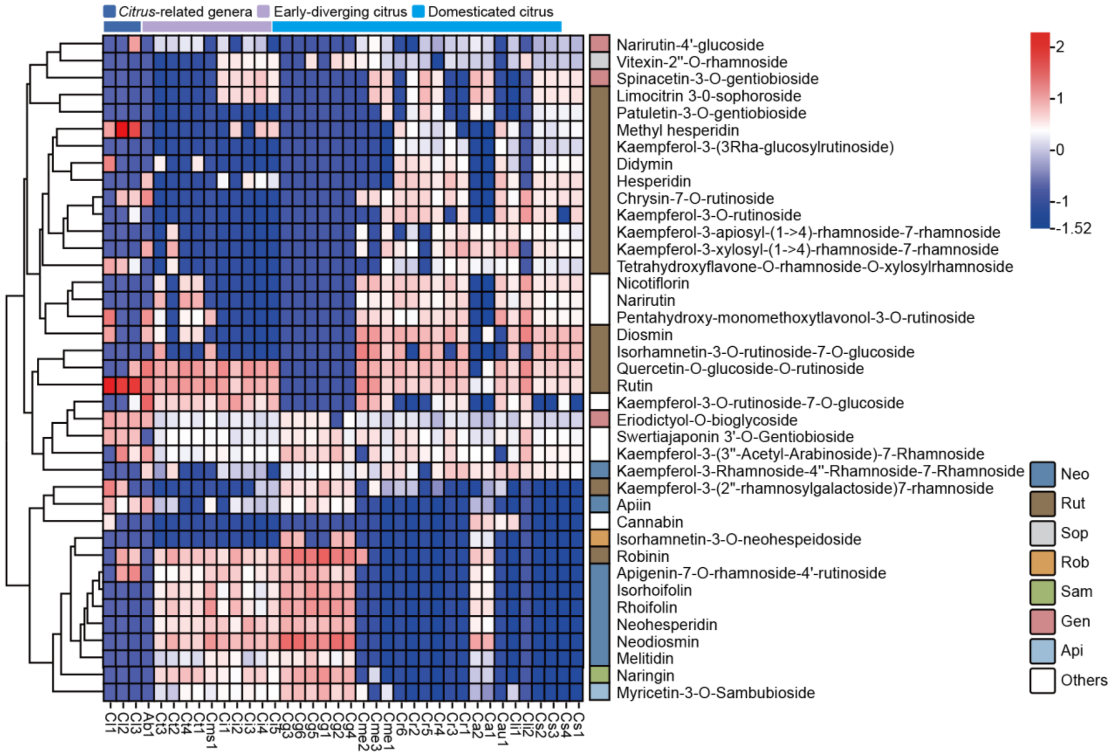

Supplemental Figure 2. Heatmap of flavonoid disaccharide glycosides among 38 citrus accessions. Each row represents a compound, and each column represents a citrus accession. Color intensity indicates relative metabolite abundance, with values standardized by Z-score transformation across accessions (column-wise normalization). The top annotation indicates the classification of citrus accessions. The right annotation denotes the type of disaccharide glycoside; compounds that could not be assigned to the seven defined types are grouped as 'Others'. Neo, neohesperidoside; Rut, rutinoside; Sop, sophoroside; Rob, robinobioside; Sam, sambubioside; Gen, gentiobioside; Api, apiosylrhamnoside.

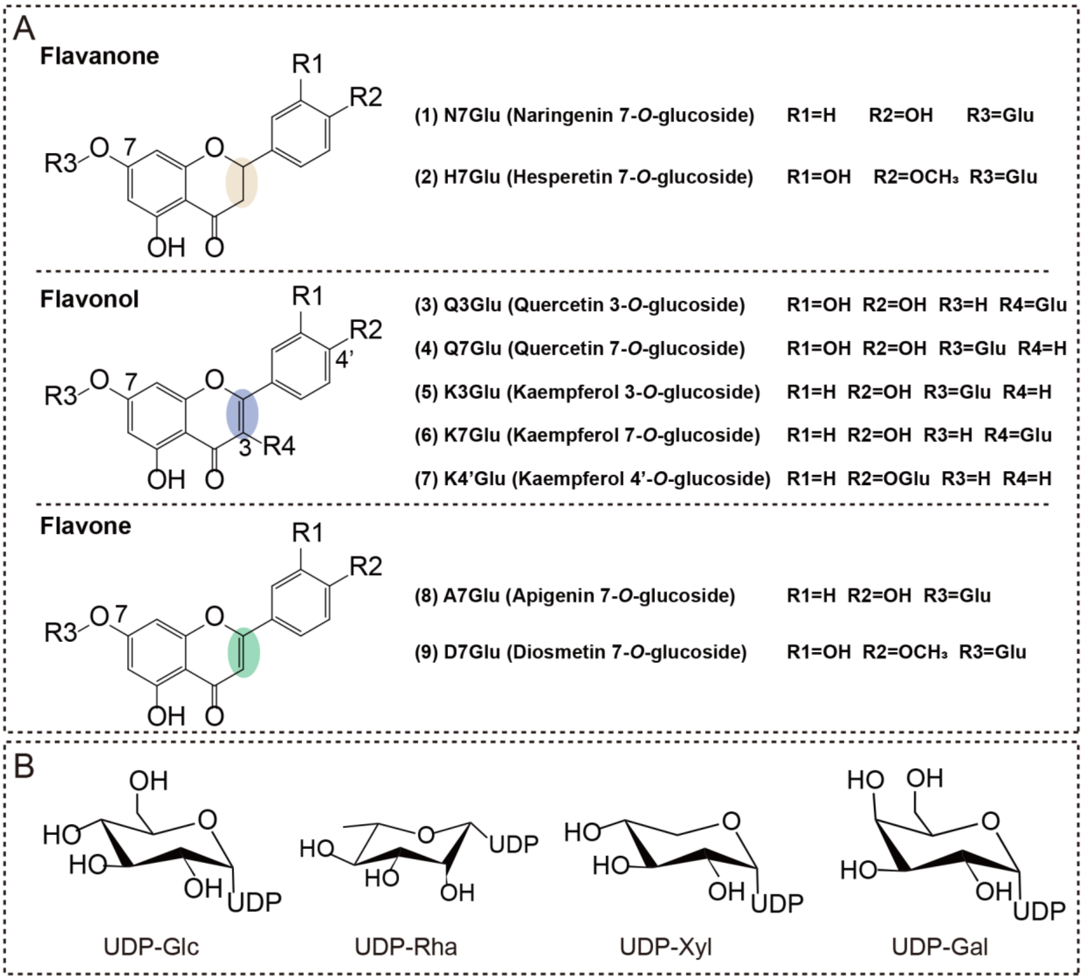

Supplemental Figure 3. Structures of flavonoid substrates and sugar donors used for functional assays. (A) Chemical structures and abbreviations of flavonoid substrates used for in vitro functional verification. Color blocks represent the structural differences on the core skeleton among the three flavonoid subgroups. (B) Chemical structures of sugar donors in the assays.

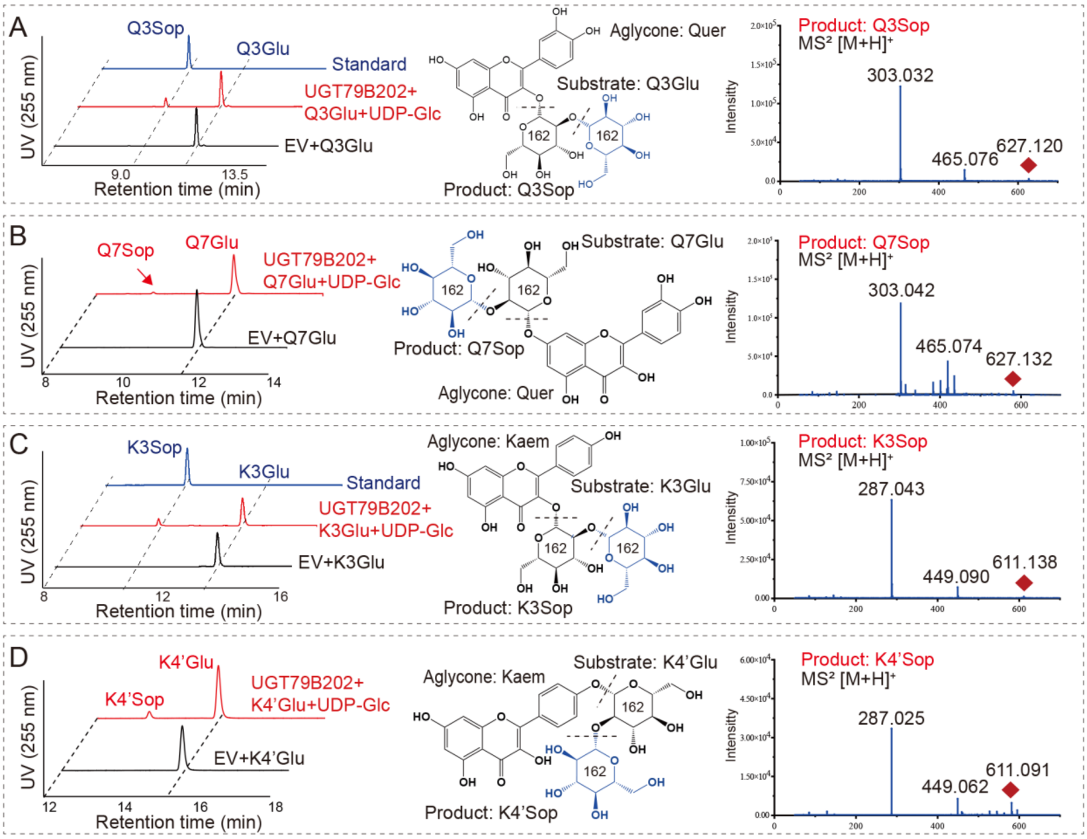

Supplemental Figure 4. Identification of enzymatic products catalyzed by UGT79B202 using flavonol mono-glucosides and UDP-Glc as substrates. (A-D) HPLC chromatograms, chemical structures of reaction products, and LC/MS fragmentation spectra (positive ion mode) for reaction using Q3Glu (A), Q7Glu (B), K3Glu (C) and K4'Glu (D) used as substrate. In chromatograms, blue traces represent authentic standards, red traces represent reactions with UGT79B202, and black traces correspond to empty vector (EV) controls. The glycosyl moiety transferred by the enzyme is highlighted in blue in the product structures. In MS/MS spectra, precursor ions are marked with red diamonds. Quer, quercetin; Q3Glu, quercetin 3-*O*-glucoside; Q3Sop, quercetin 3-*O*-sophoroside; Q7Glu, quercetin 7-*O*-glucoside; Q7Sop, quercetin 7-*O*-sophoroside; Kaem, kaempferol; K3Glu, kaempferol 3-*O*-glucoside; K3Sop, kaempferol 3-*O*-sophoroside; K4'Glu, kaempferol 4'-*O*-glucoside; K4'Sop, kaempferol 4'-*O*-sophoroside. EV, empty vector pMAL-c2x.

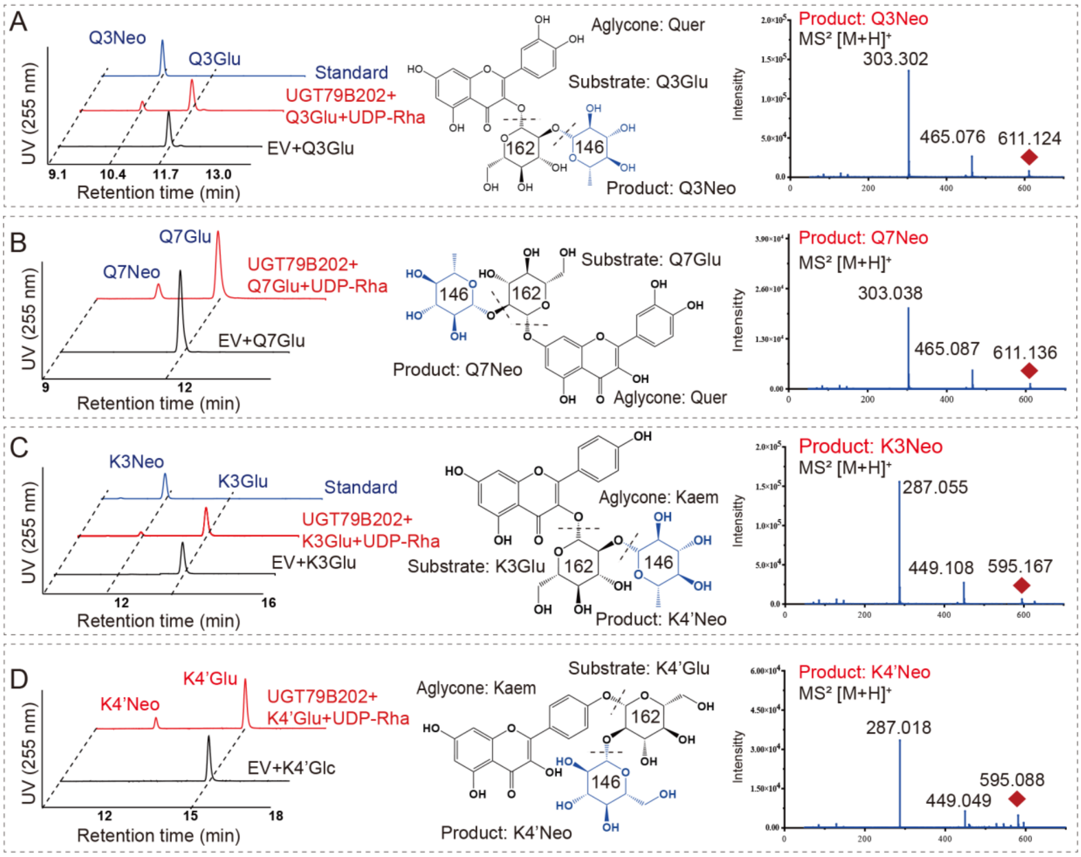

Supplemental Figure 5. Identification of enzymatic products catalyzed by UGT79B202 using flavonol mono-glucosides and UDP-Rha as substrates. (A-D) HPLC chromatograms, chemical structures of reaction products, and LC/MS fragmentation spectra (positive ion mode) for reaction using Q3Glu (A), Q7Glu (B), K3Glu (C) and K4'Glu (D) used as substrate. In chromatograms, blue traces represent authentic standards, red traces represent reactions with UGT79B202, and black traces correspond to empty vector (EV) controls. The glycosyl moiety transferred by the enzyme is highlighted in blue in the product structures. In MS/MS spectra, precursor ions are marked with red diamonds. Quer, quercetin; Q3Glu, quercetin 3-*O*-glucoside; Q3Neo, quercetin 3-*O*-neohesperidoside; Q7Glu, quercetin 7-*O*-glucoside; Q7Neo, quercetin 7-*O*-neohesperidoside; Kaem, kaempferol; K3Glu, kaempferol 3-*O*-glucoside; K3Neo, kaempferol 3-*O*-neohesperidoside; K4'Glu, kaempferol 4'-*O*-glucoside; K4'Neo, kaempferol 4'-*O*-neohesperidoside. EV, empty vector pMAL-c2x.

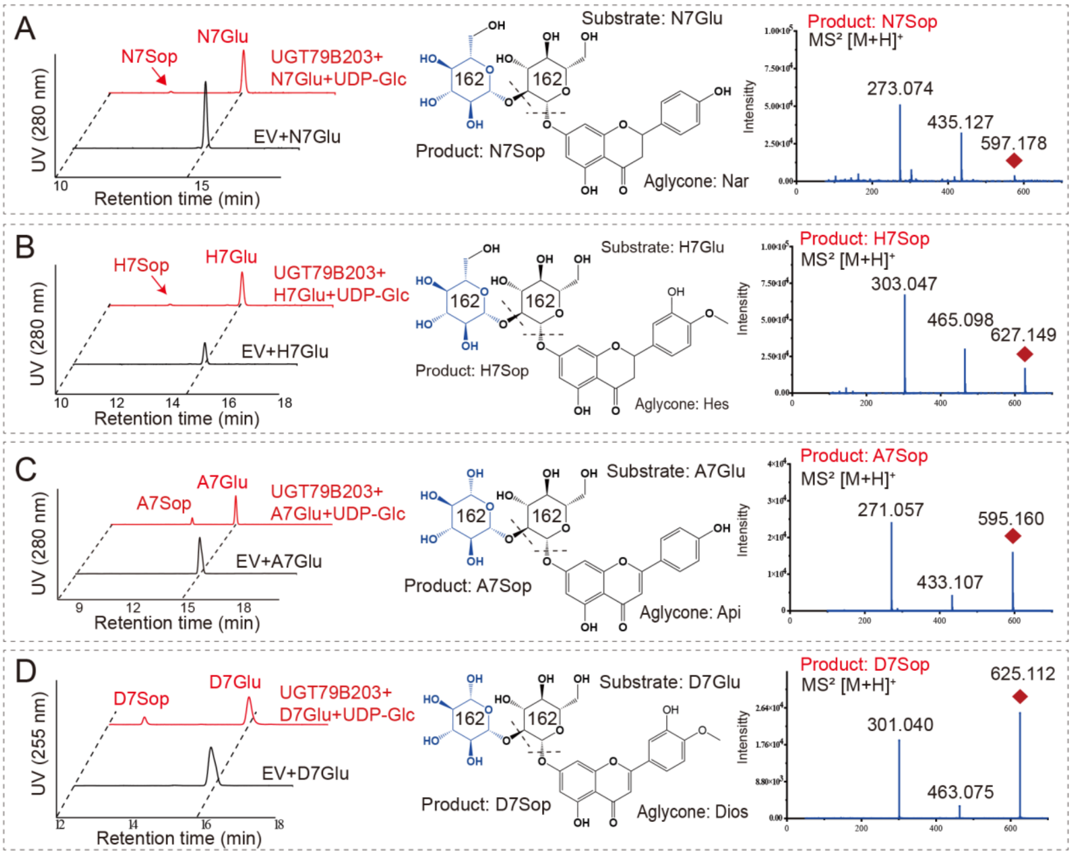

Supplemental Figure 6. Identification of enzymatic products catalyzed by UGT79B203 using flavanone and flavone mono-glucosides and UDP-Glc as substrates. (A-D) HPLC chromatograms, chemical structures of reaction products, and MS/MS fragmentation spectra (positive ion mode) for reactions using N7Glu (A), H7Glu (B), A7Glu (C), and D7Glu (D) used as substrate. In chromatograms, blue traces represent authentic standards, red traces represent reactions with UGT79B202, and black traces correspond to empty vector (EV) controls. The glycosyl moiety transferred by the enzyme is highlighted in blue in the product structures. In MS/MS spectra, precursor ions are marked with red diamonds. Nar, naringenin; N7Glu, naringenin 7-*O*-glucoside; N7Sop, naringenin 7-*O*-sophoroside; Hes, hesperetin; H7Glu, hesperetin 7-*O*-glucoside; H7Sop, hesperetin 7-*O*-sophoroside; Api, apigenin; A7Glu, apigenin 7-*O*-glucosid; A7Sop, apigenin 7-*O*-sophoroside; Dios, diosmetin; D7Glu, diosmetin 7-*O*-glucoside; D7Sop, diosmetin 7-*O*-sophoroside. EV, empty vector pMAL-c2x.

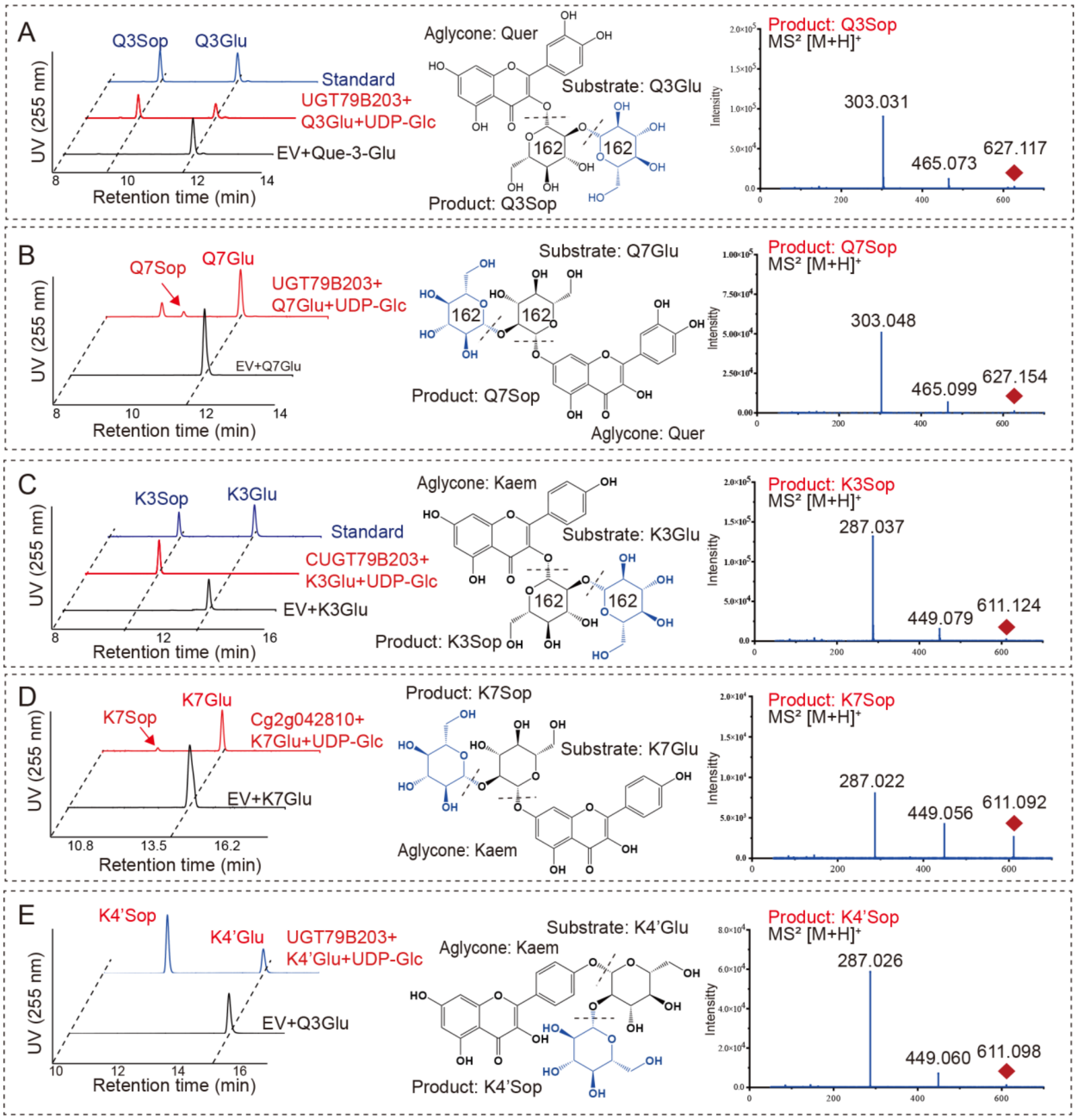

Supplemental Figure 7. Identification of enzymatic products catalyzed by UGT79B203 using flavonol mono-glucosides and UDP-Glc as substrates. (A-E) HPLC chromatograms, chemical structures of reaction products, and MS/MS fragmentation spectra (positive ion mode) for reactions using Q3Glu (A), Q7Glu (B), K3Glu (C), K7Glu (D) and K4'Glu (E) used as substrate. In chromatograms, blue traces represent authentic standards, red traces represent reactions with UGT79B202, and black traces correspond to empty vector (EV) controls. The glycosyl moiety transferred by the enzyme is highlighted in blue in the product structures. In MS/MS spectra, precursor ions are marked with red diamonds. Que, quercetin; Q3Glu, quercetin 3-*O*-glucoside; Q3Sop, quercetin 3-*O*-sophoroside; Q7Glu, quercetin 7-*O*-glucoside; Q7Sop, quercetin 7-*O*-sophoroside; Kaem, kaempferol; K3Glu, kaempferol 3-*O*-glucoside; K3Sop, kaempferol 3-*O*-sophoroside; K7Glu, kaempferol 7-*O*-glucoside; K7Sop, kaempferol 7-*O*-sophoroside; K4'Glu, kaempferol 4'-*O*-glucoside; K4'Sop, kaempferol 4'-*O*-sophoroside. EV, empty vector pMAL-c2x.

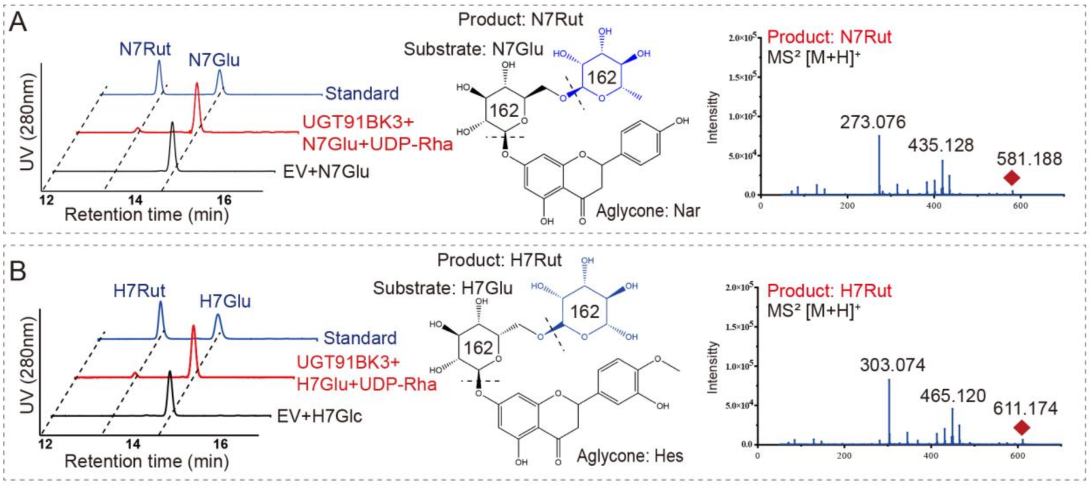

Supplemental Figure 8. Identification of enzymatic products catalyzed by UGT91BK3 using flavanone mono-glucosides and UDP-Rha as substrates. (A-B) HPLC chromatograms, chemical structures of reaction products, and MS/MS fragmentation spectra (positive ion mode) for reactions using N7Glu (A) and H7Glu (B) as substrates. In the chromatograms, blue traces represent authentic standards, red traces represent reactions with UGT91BK3, and black traces correspond to empty-vector (EV) controls. The transferred sugar moiety is highlighted in blue in the product structures. In MS/MS spectra, precursor ions are marked with red diamonds. N7Rut, naringenin 7-*O*-rutinoside; N7Glu, naringenin 7-*O*-glucoside; H7Rut, hesperetin 7-*O*-rutinoside; H7Glu, hesperetin 7-*O*-glucoside. EV, empty vector pMAL-c2x.

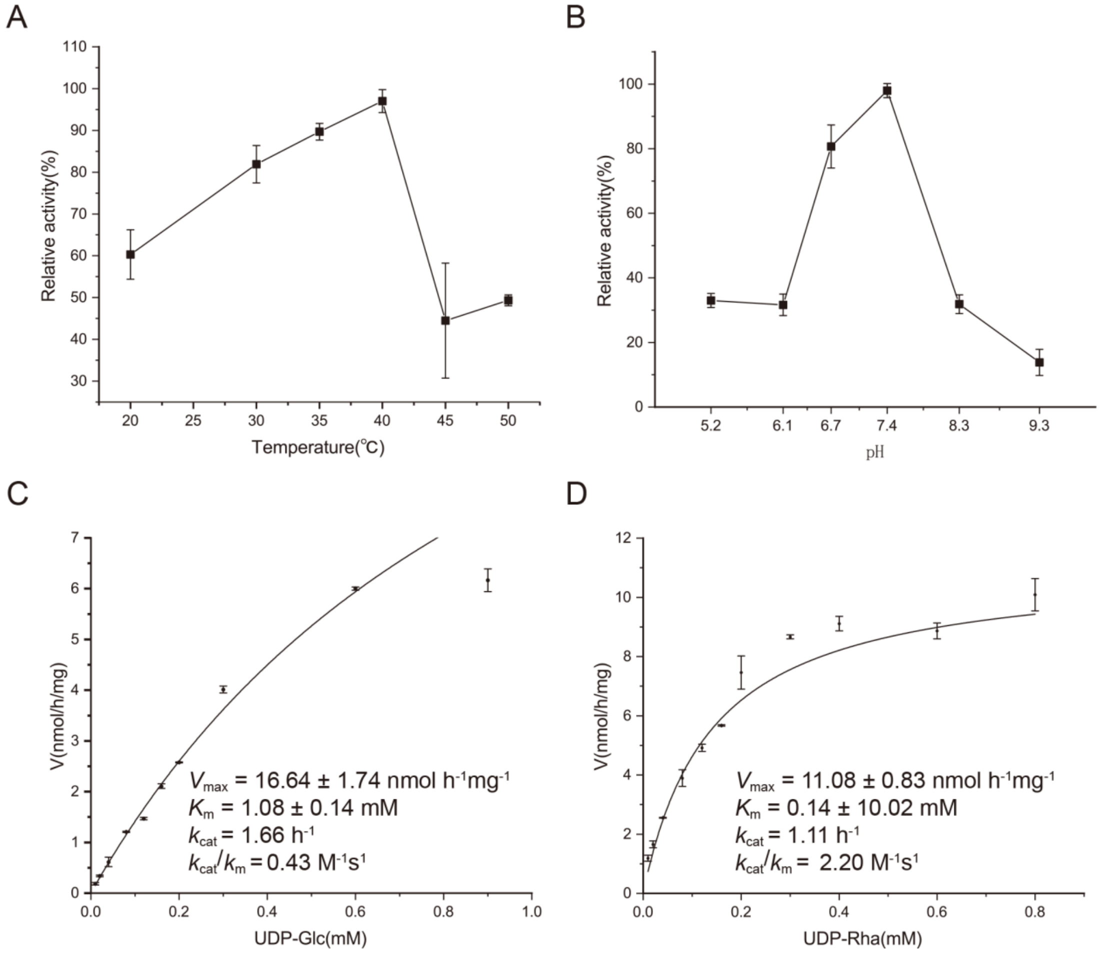

Supplemental Figure 9. Biochemical characterization of UGT79B202. (A and B) Effects of temperature (A) and pH (B) on enzyme activity. (C and D) Kinetic analysis with UDP-Glc (C) and UDP-Rha (D) as the sugar donor. All assays were performed in triplicate (mean ± SD).

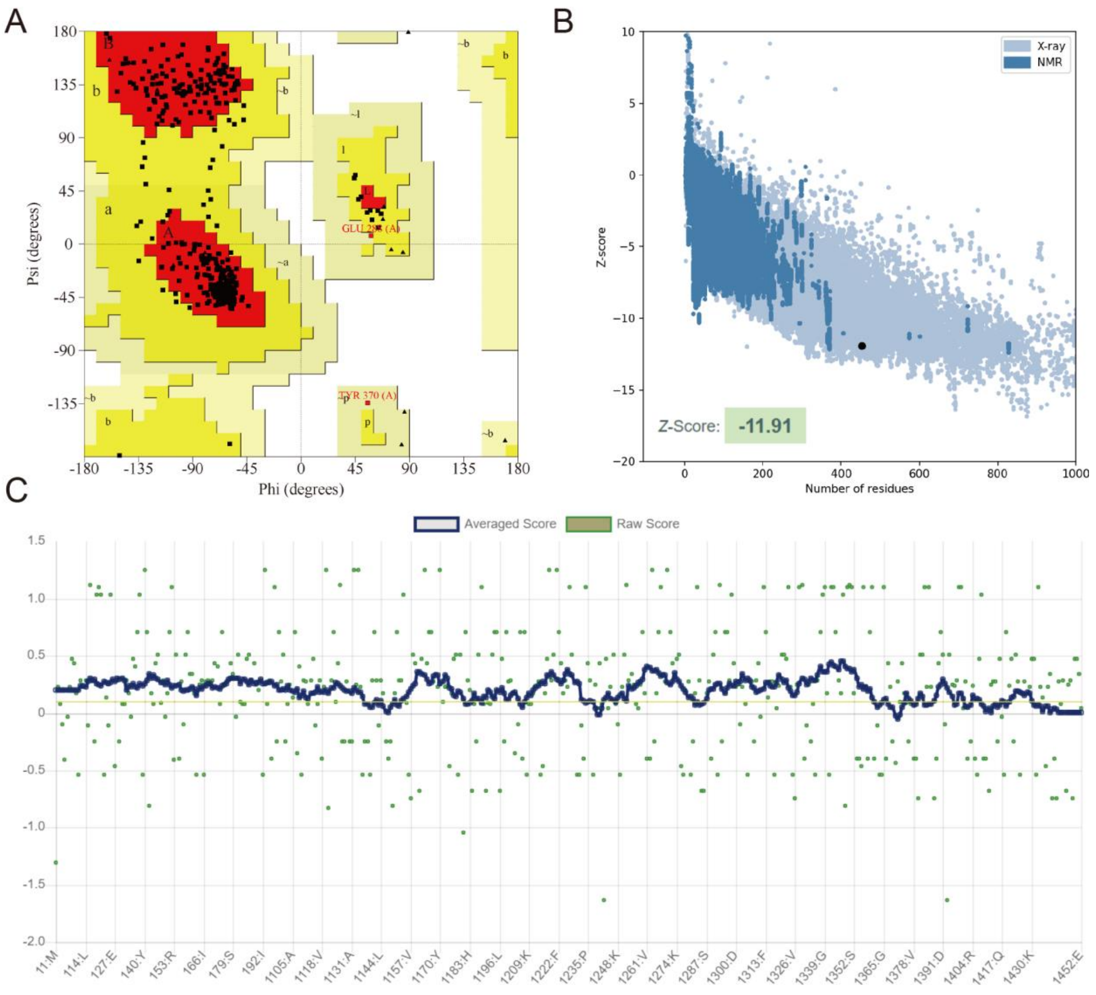

Supplemental Figure 10. Protein conformational rationality assessment of Cm1,2RhaT. (A) The Ramachandran plot analysis of Cm1,2RhaT protein (86.7% most favored, 13.1% additional allowed, 0.3% generously allowed, 0.0% disallowed). (B) Evaluation results of ProSA for Cm1,2RhaT protein. The overall model Z-score of -11.91 lies well within the range of scores typically found for native proteins of similar size determined by X-ray crystallography (light blue area). (C) The 3D-1D score plots of each residue in Cm1,2RhaT. 83.85% of the residues have averaged 3D-1D score >= 0.1.

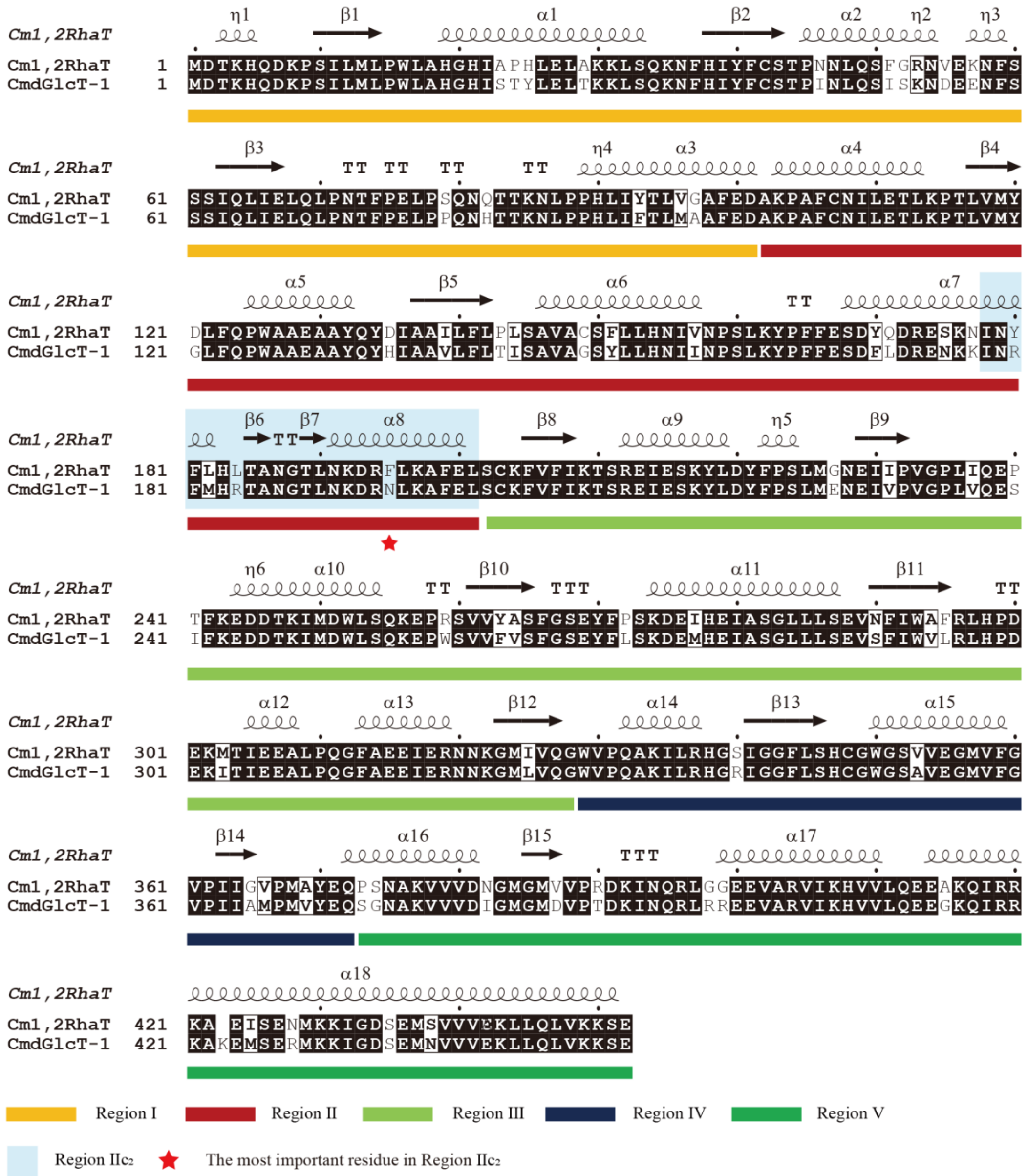

Supplemental Figure 11. Sequence alignment and region division of Cm1,2RhaT and CmdGlcT-1. Amino acid sequences of Cm1,2RhaT and CmdGlcT-1 were aligned and annotated with predicted secondary structural elements, including α-helices (α), β-strands (β), η-helices (η), and turns (T). Five regions (Region I–V) were defined based on sequence segmentation. Region IIc2, which plays a key role in determining sugar donor specificity, is highlighted with a blue color block, and the critical amino acid residue Phe195 is marked with a red five-pointed star.

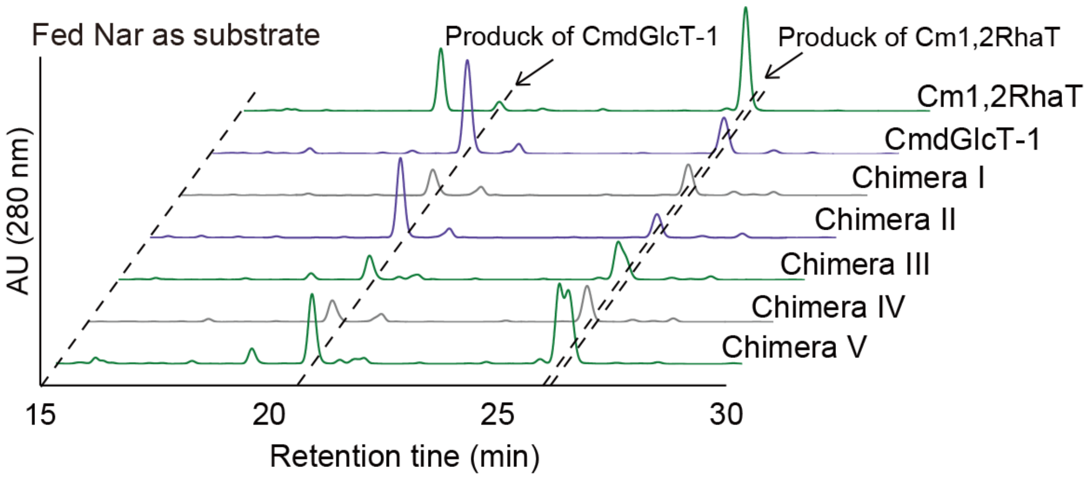

Supplemental Figure 12. Functional analysis of chimeric proteins in BY2 transgenic cell using Nar as substrate. Nar, narigenin.The dashed line represents a characteristic peak used to distinguish compounds with similar retention times. To better illustrate the functional differences, chromatograms are color-coded: green indicates proteins exhibiting 1,2RhaT activity, purple indicates proteins with dGlcT activity, and gray indicates proteins lacking both activities.

.

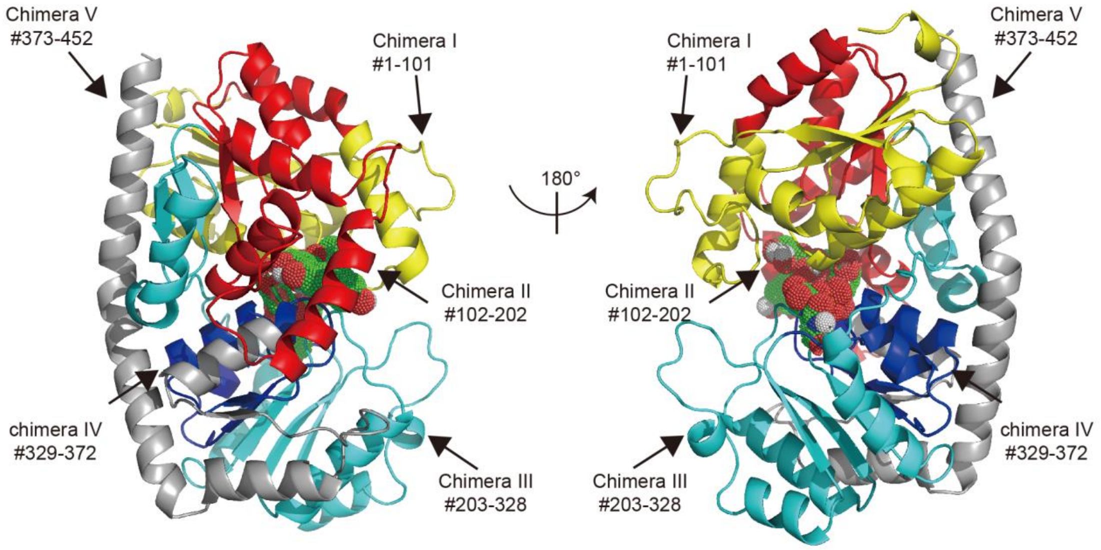

Supplemental Figure 13. Schematic representation of region division in Cm1,2RhaT protein. The protein is divided into five regions (Region I–V), with the corresponding amino acid positions indicated beside each segment. The docked substrate molecules are shown as dots.

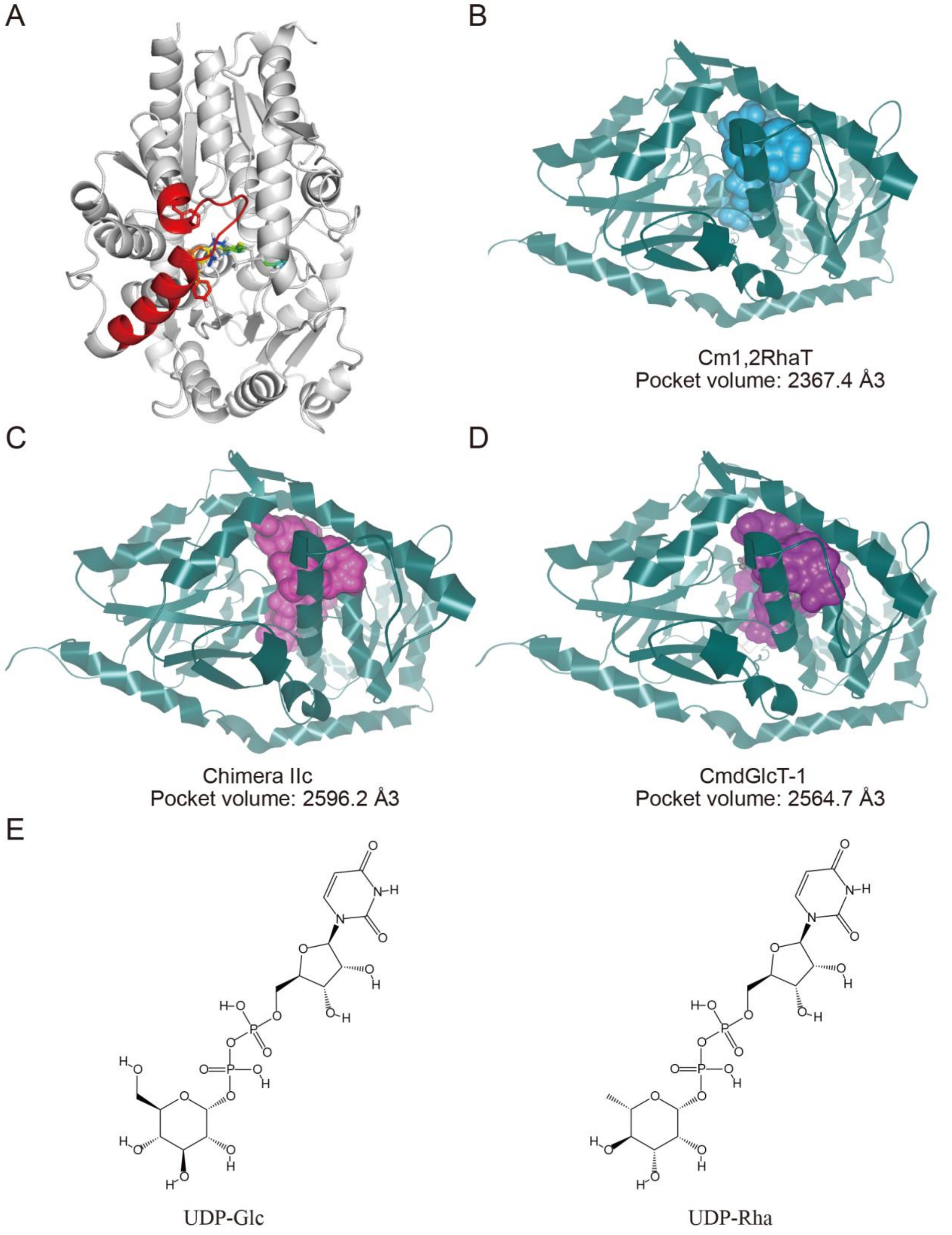

Supplemental Figure 14. Comparison of pocket sizes among Cm1,2RhaT, Chimeric IIc, and CmdGlcT-1. (A) Location of the region IIc (highlighted in red) on the Cm1,2RhaT structure. (B-D) Predicted pocket size of Cm1,2RhaT (B), Chimera IIc (C) and CmdGlcT-1 (D). (E) Chemical structures of the sugar donors UDP-glucose (UDP-Glc) and UDP-rhamnose (UDP-Rha). Functional assays showed that substitution of region IIc led to a shift from rhamnosylation to glucosylation, demonstrating that region IIc plays a critical role in determining sugar donor preference.

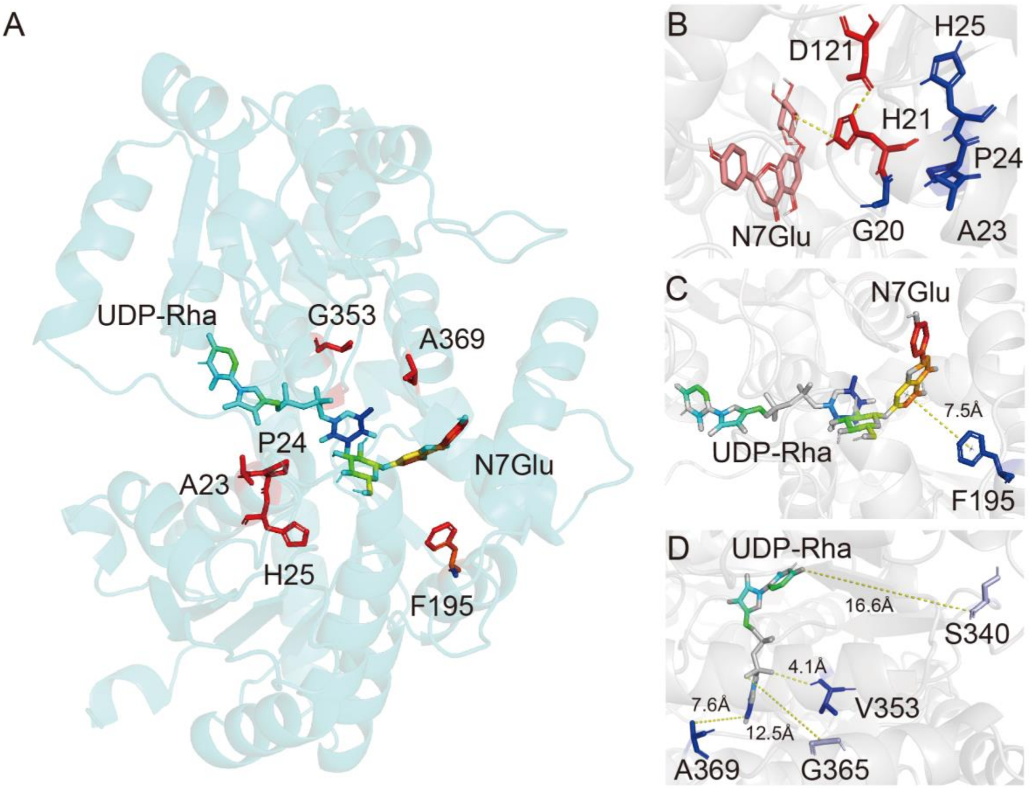

Supplemental Figure 15. Structural location of key differential amino acid residues identified by domain swapping. (A) The location of the key differential amino acid residues onto the Cm1,2RhaT protein structure. (C-E) Structural location and/or distance of key differential residues within region I (C), region II (D) and region V (E) of Cm1,2RhaT.

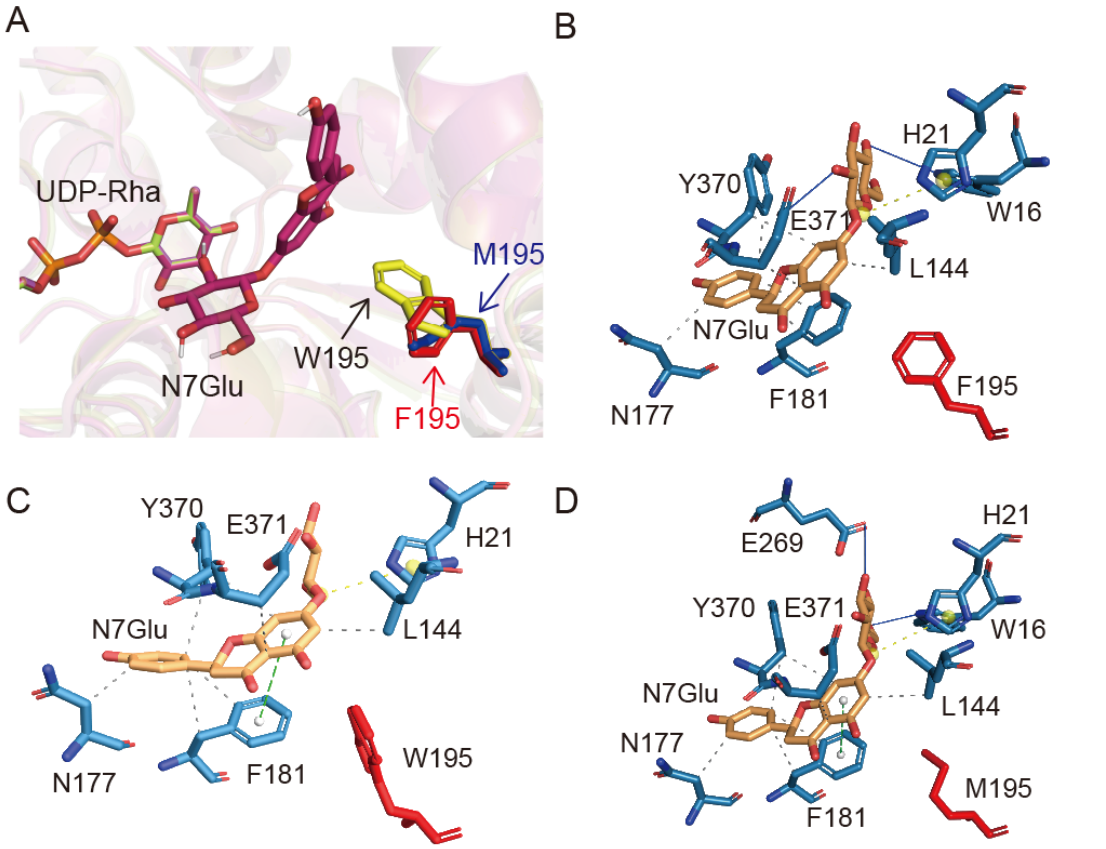

Supplemental Figure 16. Interaction analysis of representative mutations at residues 195 of Cm1,2RhaT. (A) Location of represents mutants of residues 195 on the Cm1,2RhaT structure. (B-D) Interaction analysis of Cm1,2RhaT-F195(B), Cm1,2RhaT-F195W (C) and Cm1,2RhaT-F195M (D) with N7Glu (naringin 7-*O*-glucoside). Hydrogen bonds are represented by solid blue lines, hydrophobic interactions as gray dashed line, salt bridges as yellow dotted line, and π–stacking by green dashed lines. The residue at position 195 is highlighted in red sticks.

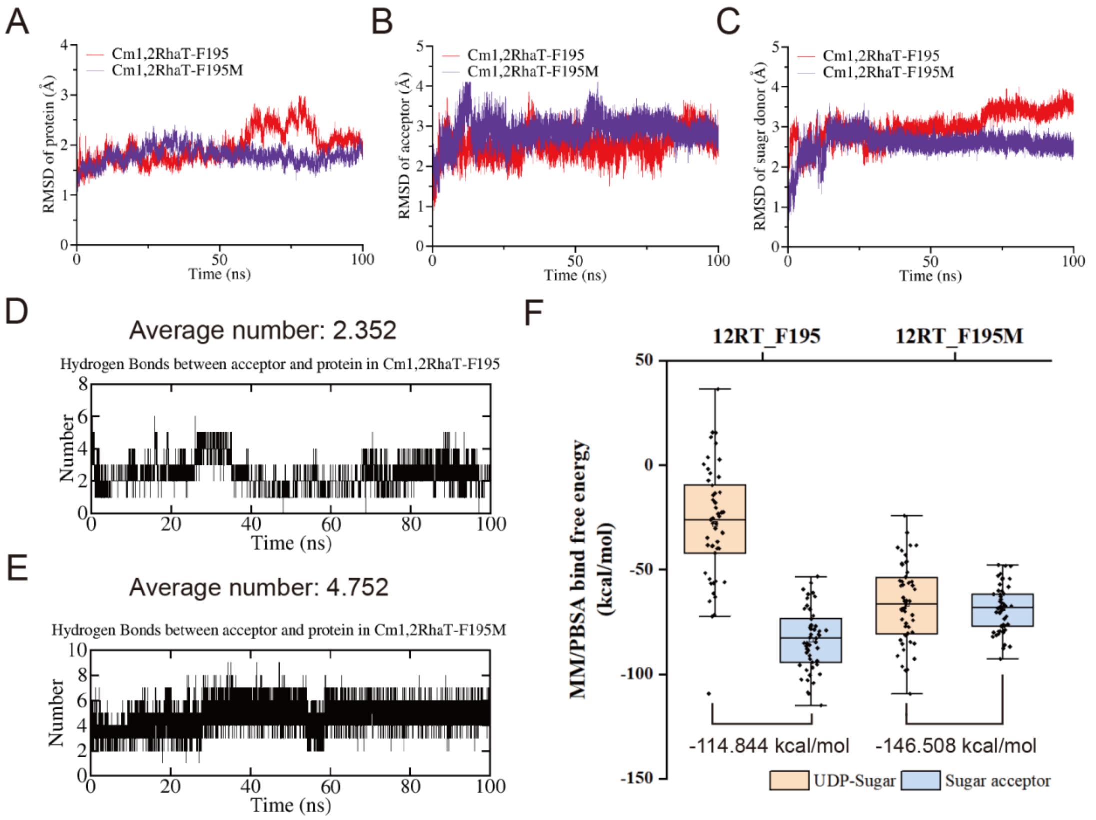

Supplemental Figure 17. Molecular dynamics simulations of Cm1,2RhaT-F195-UDP-Rha-A7Glu and Cm1,2RhaT-F195M-UDP-Rha-A7Glu. (A-C) Root mean square deviation (RMSD) of protein backcone (A), acceptor substrate A7Glu (B), and sugar donor UDP-Rha (C) during the simulation. (D-E) Number of hydrogen bonds between the protein and acceptor substrate in Cm1,2RhaT-F195 (D) and Cm1,2RhaT-F195M (E). (F) Binding free energy analysis of Cm1,2RhaT-F195 and Cm1,2RhaT-F195M.

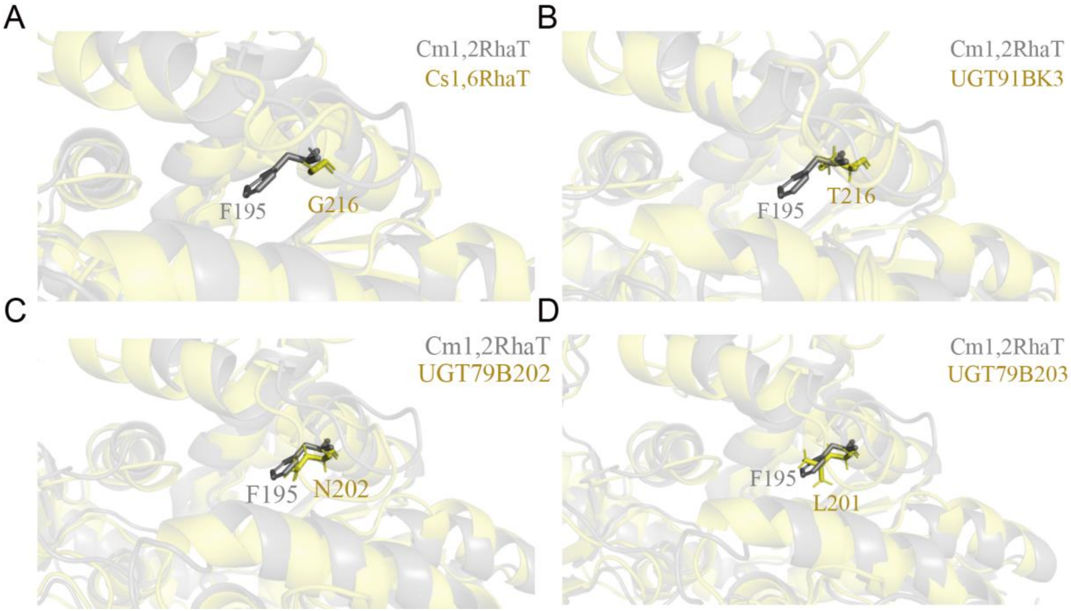

Supplemental Figure 18. Superimposition of Cm1,2RhaT with other dGlyTs to identify the residue corresponding to Phe195. (A-D) Superimposition of Cm1,2RhaT with Cs1,6RhaT (A), UGT91BK3 (B), UGT79B202 (C), and UGT79B203 (D), respectively.

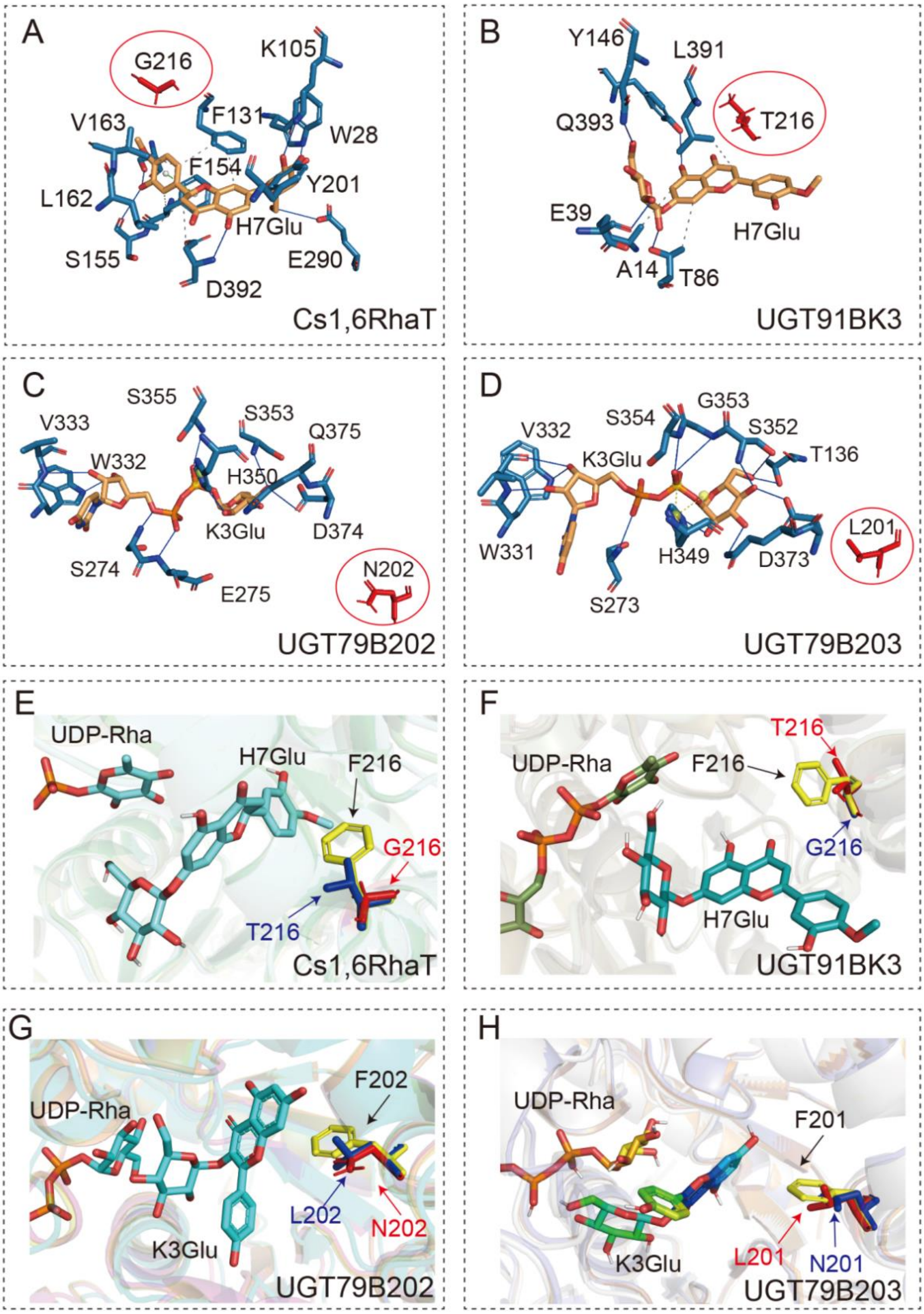

Supplemental Figure 19. Substrate interactions and the position of the residue corresponding to Phe195 in citrus dGlyTs. (A-D) Substrate interacting residues and the residue corresponding to Cm1,2RhaT-Phe195 in Cs1,6RhaT (A), UGT91BK3 (B), UGT79B202 (C), and UGT79B203 (D). (E–H) Structural positions of the corresponding residue 195 and its mutants in Cs1,6RhaT (E), UGT91BK3 (F), UGT79B202 (G), and UGT79B203 (H). Hydrogen bonds are represented as solid blue lines, hydrophobic interactions as gray dashed line, and salt bridges as yellow dotted line. Residues corresponding to Phe195 are highlighted in red stick.

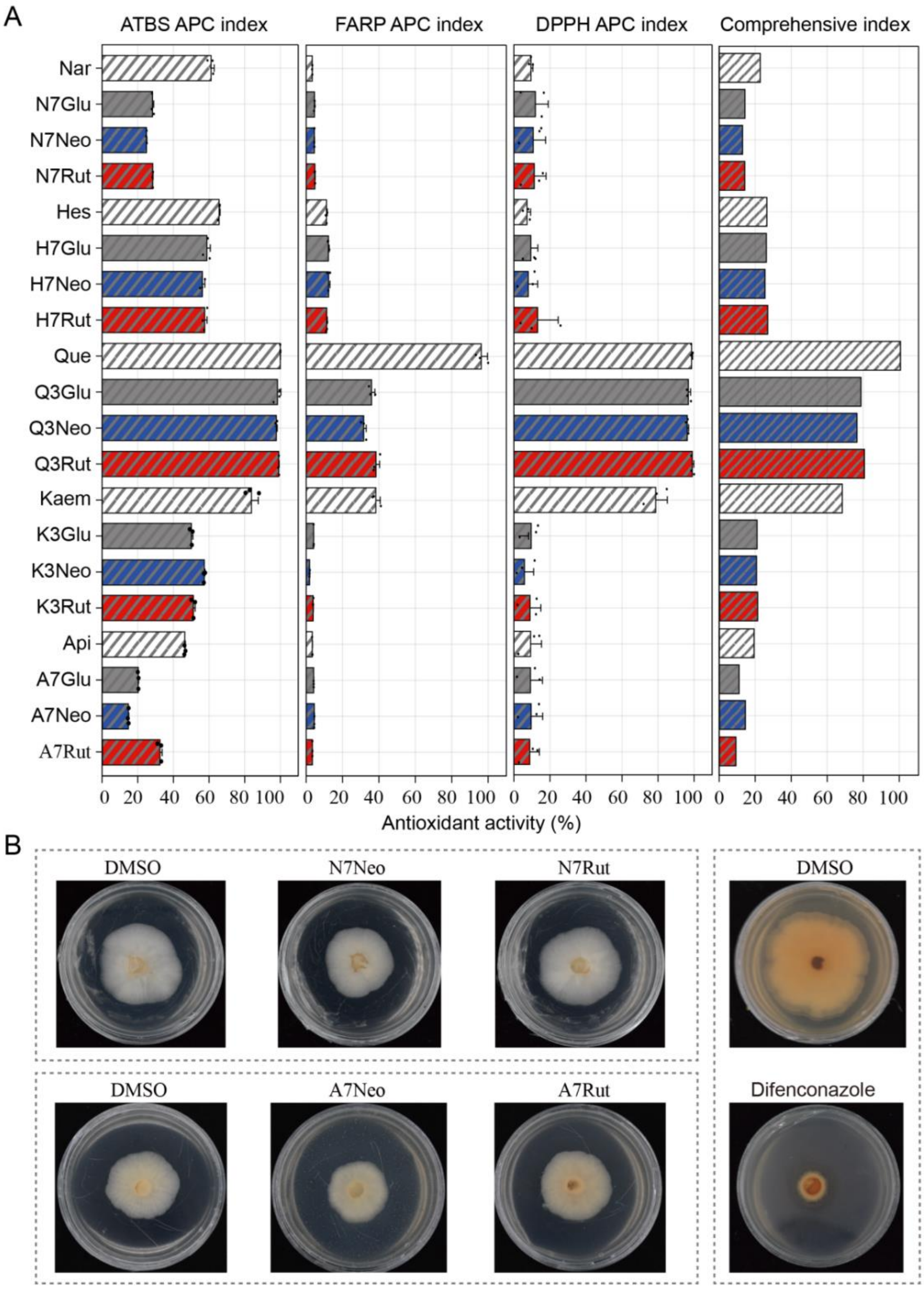

Supplemental Figure 20. Antioxidant and antifungal activities of representative compounds. (A) Comprehensive comparison of 20 flavonoid compounds. Antioxidant capacity was evaluated using three assays: ABTS (2,2'-azinobis-(3-ethylbenzothiazoline-6-sulfonic acid) radical scavenging assay), FRAP (ferric reducing antioxidant power assay), and DPPH (2,2-diphenyl-1-picrylhydrazyl radical scavenging assay). A combined antioxidant index was calculated as the mean of the values from the three assays. (B) Representative image of fungal growth inhibition by selected compounds. Experiments were performed with at least three biological replicates per compound. Difenoconazole served as the positive control.

A

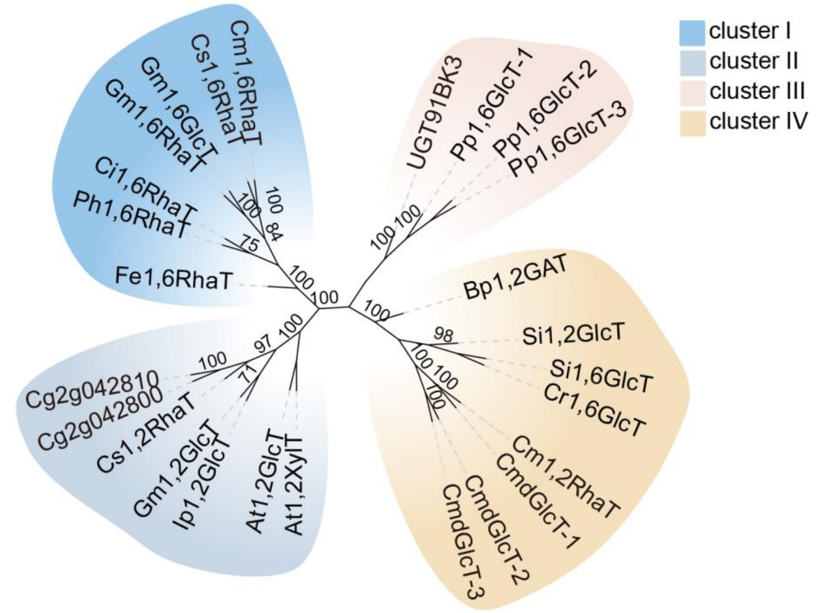

B

	protein	catalytic dyay			sugar donor recognition				domain-swapping					
cluster I	Cs1,6RhaT	G32	H33	D130	V153	S372	D392	N396	S35	P36	F37	G216	V374	K390
	Cm1,6RhaT	G32	H33	D130	V153	S372	D392	N396	R35	P36	F37	G216	V374	K390
	Gm1,6RhaT	G23	H24	D115	V137	F360	D381	N385	S26	P27	F28	V206	V363	K379
	Gm1,6GlcT	G22	H23	D114	S136	F359	D380	N384	N25	A26	F27	V205	V362	K378
	Ph1,6RhaT	G23	H24	D121	V143	F364	D385	N389	S26	P27	F28	I210	V367	K383
	Ci1,6RhaT	G21	H22	D120	V142	F360	D381	N385	S24	P25	F26	F206	V363	K379
	Fe1,6RhaT	G21	H22	D119	V141	F359	D380	N384	N24	P25	F26	N206	V362	K378
cluster II	UGT79B202	G17	H18	D115	I136	S350	D374	N378	T20	P21	F22	N202	L356	V372
	UGT79B203	G17	H18	D115	T136	S352	D373	N377	T20	S21	Y22	L201	L355	V371
	Cs1,2RhaT	G17	H18	D115	V136	Y356	D377	N381	T20	P21	F22	I205	L359	Y375
	Gm1,2GlcT	G17	H18	D115	T136	S353	D374	N378	I20	A21	F22	Q202	L356	V372
	Ip1,2GlcT	G19	H20	D117	T138	W355	D376	N380	T22	G23	F24	I204	L358	V374
	At1,2GlcT	G16	H17	D114	T135	F345	E366	N370	T19	A20	F21	I193	M348	L364
	At1,2XlyT	G23	H24	D121	I142	F364	E385	N389	T26	P27	F28	K210	M367	H383
cluster III	UGT91BK3	G16	H17	D123	A145	G372	D392	N396	M19	P20	F21	T216	V374	I390
	Pp1,6GlcT-1	G19	H20	D120	L142	W356	E377	N381	A23	P23	F24	F207	I359	V375
	Pp1,6GlcT-2	G19	H20	D123	L145	W358	D379	N383	L22	P23	F24	F209	I361	L377
	Pp1,6GlcT-3	G19	H20	D120	L142	W357	E378	N382	L22	P23	F24	L208	I360	M376
cluster IV	Cm1,2RhaT	G20	H21	D121	P143	W350	E371	N375	A23	P24	H25	F195	V353	A369
	CmdGlcT-1	G20	H21	G121	T143	W350	E371	N375	S23	T24	Y25	N195	A353	V369
	CmdGlcT-2	G21	H22	D123	T145	W351	D372	N376	T24	P25	Y26	F194	T354	V370
	CmdGlcT-3	G21	H22	D123	I145	W351	D372	N376	T24	P25	Y26	F194	T354	V370
	Cr1,6GlcT	G23	H24	D123	T145	W356	D377	N381	S26	S27	F28	E197	V359	N375
	Si1,6GlcT	G19	H20	D119	T141	W345	D366	N370	T22	P23	F24	E193	L348	H364
	Bp1,2GAT	S21	H22	D121	S143	W336	D357	N361	S24	R25	F26	F190	T339	Q355
	Si1,2GlcT	G19	H20	D121	S143	W350	D371	N375	F22	P23	F24	A195	I353	Q369

Supplemental Figure 21. Phylogenetic and structural analysis of validated important residues among plant dGlyTs. (A) Phylogenetic analysis of different plant dGlyTs from citrus and other plant. (B) Structural alignment of representative dGlyTs showing the positions corresponding to functionally validated residues of Cm1,2RhaT. Key amino acids identified in this study are highlighted in yellow.

A

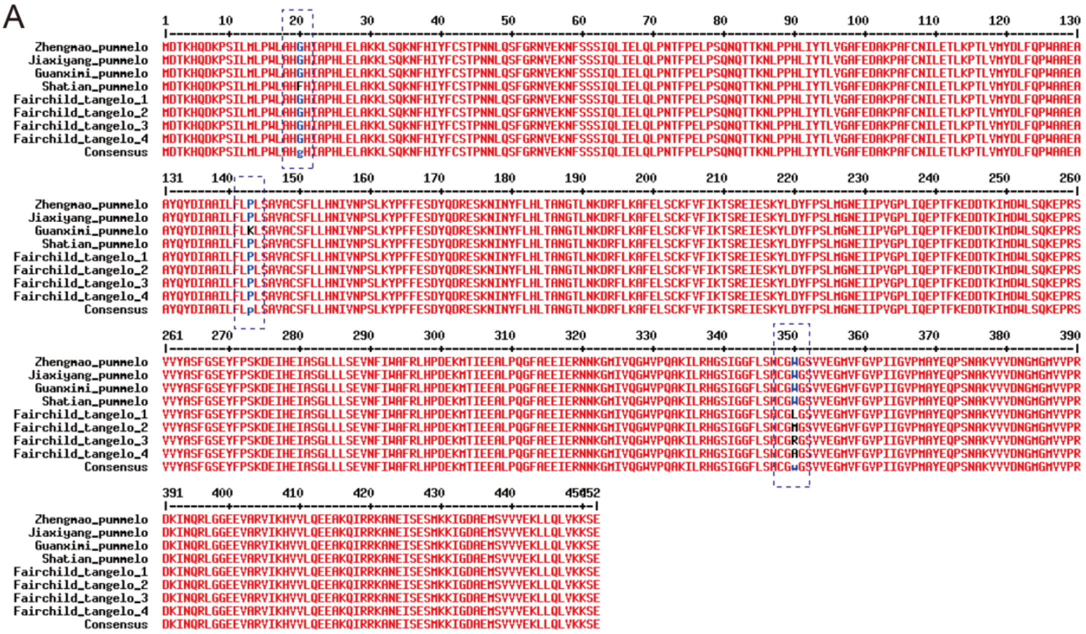

B

Germplasm	Normal sequence	Mutant sequence
Zhengmao pummelo	+	-
Jiaxiyang pummelo	+	-
Shatian pummelo	+	#143 ： Pro→ Lys
Guanximi pummelo	+	#20 ： Gly→Phe
Fairchild tangelo	-	#350 : Trp→Leu 、Met 、Ala 、Arg

C

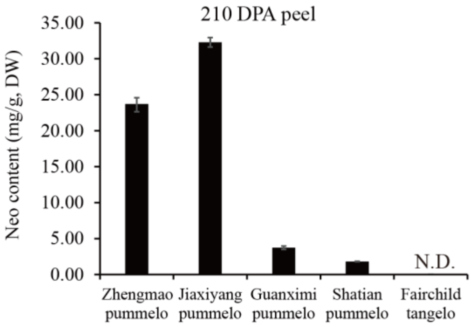

Supplemental Figure 22. Sequence variation of Cm1,2RhaT and accumulation of Neo in different citrus accessions. (A) Sequence alignment of Cm1,2RhaT from representative citrus accessions. Variant positions are highlighted with blue boxes. (B) Summary of Cm1,2RhaT allelic variation across different accessions. ‘+’ indicates the presence of a sequence variant; ‘–’ indicates no variation compared to the reference sequence. (C) Accumulation level of Neo in the corresponding accessions.

235 **Supplemental Table 1. Thirty-five plant accessions used for metabolite profiling in this study.**

No.	Abbreviation	Accession Common name	Scientific name	Tissue sampled	Catalog	Collection site
1	Cl1	Guilin wampee	*Clausena lansium*	mature fruit peel (flavedo)	*Citrus*-related genera	Guilin, Guangxi
2	Cl2	Huanong wampee	*C. lansium*	mature fruit peel (flavedo)	*Citrus*-related genera	Wuhan, Hubei
3	Cl3	Jixin wampee	*C. lansium*	mature fruit peel (flavedo)	*Citrus*-related genera	Guilin, Guangxi
4	Ab1	Chinese box orange	*Atalantia buxfoliata*	mature fruit peel (flavedo)	*Citrus*-related genera	Wuhan, Hubei
5	Cms1	Mangshanyegan	*C. mangshanensis*	mature fruit peel (flavedo)	Early-diverging citrus	Wuhan, Hubei
6	Ci1	Ningqiang No.2	*C. ichangensis*	mature fruit peel (flavedo)	Early-diverging citrus	Hanzhong, Shanxi
7	Ci2	Ningqiang No.4	*C. ichangensis*	mature fruit peel (flavedo)	Early-diverging citrus	Hanzhong, Shanxi
8	Ci3	Ningqiang No.5	*C. ichangensis*	mature fruit peel (flavedo)	Early-diverging citrus	Hanzhong, Shanxi
9	Ci4	Ningqiang No.8	*C. ichangensis*	mature fruit peel (flavedo)	Early-diverging citrus	Hanzhong, Shanxi
10	Ci5	Ningqiang No.9	*C. ichangensis*	mature fruit peel (flavedo)	Early-diverging citrus	Hanzhong, Shanxi
11	Ct1	Flying dragon trifoliated orange	*C. trifoliata*	mature fruit peel (flavedo)	Early-diverging citrus	Beibei, Chongqing
12	Ct2	Daguo trifoliated orange	*C. trifoliata*	mature fruit peel (flavedo)	Early-diverging citrus	Beibei, Chongqing
13	Ct3	Japanese trifoliated orange	*C. trifoliata*	mature fruit peel (flavedo)	Early-diverging citrus	Chenggu, Shanxi
14	Ct4	Donghai trifoliated orange	*C. trifoliata*	mature fruit peel (flavedo)	Early-diverging citrus	Beibei, Chongqing
15	Cg1	Huanong red pummelo	*C. grandis*	mature fruit peel (flavedo)	Domesticated citrus	Wuhan, Hubei
16	Cg2	Jiaxiyang pummelo	*C. grandis*	mature fruit peel (flavedo)	Domesticated citrus	Wuhan, Hubei
17	Cg3	Beibei pummelo	*C. grandis*	mature fruit peel (flavedo)	Domesticated citrus	Beibei, Chongqing
18	Cg4	Taiguoqing pummelo	*C. grandis*	mature fruit peel (flavedo)	Domesticated citrus	Beibei, Chongqing
19	Cg5	Guanximi pummelo	*C. grandis*	mature fruit peel (flavedo)	Domesticated citrus	Beibei, Chongqing
20	Cg6	Fenghuang pummelo	*C. grandis*	mature fruit peel (flavedo)	Domesticated citrus	Beibei, Chongqing
21	Cr1	Satsuma mandarin	*C. reticulata*	mature fruit peel (flavedo)	Domesticated citrus	Beibei, Chongqing
22	Cr2	Red tangerine	*C. reticulata*	mature fruit peel (flavedo)	Domesticated citrus	Beibei, Chongqing

23	Cr3	Nanfengmi tangerine	*C. reticulata*	mature fruit peel (flavedo)	Domesticated citrus	Beibei, Chongqing
24	Cr4	Shatangju mandarin	*C. reticulata*	mature fruit peel (flavedo)	Domesticated citrus	Beibei, Chongqing
25	Cr5	Beni Madonna	*C. reticulata*	mature fruit peel (flavedo)	Domesticated citrus	Beibei, Chongqing
26	Cr6	Shiranui	*C. reticulata*	mature fruit peel (flavedo)	Domesticated citrus	Beibei, Chongqing
27	Cs1	Taoyesweet orange	*C. sinensis*	mature fruit peel (flavedo)	Domesticated citrus	Beibei, Chongqing
28	Cs2	Bingtangcheng	*C. sinensis*	mature fruit peel (flavedo)	Domesticated citrus	Beibei, Chongqing
29	Cs3	Cara cara navel orange	*C. sinensis*	mature fruit peel (flavedo)	Domesticated citrus	Beibei, Chongqing
30	Cs4	Newhall navel orange	*C. sinensis*	mature fruit peel (flavedo)	Domesticated citrus	Beibei, Chongqing
31	Ca1	Daidai sour range	*C. aurantium*	mature fruit peel (flavedo)	Domesticated citrus	Beibei, Chongqing
32	Ca2	Brazil sour range	*C. aurantium*	mature fruit peel (flavedo)	Domesticated citrus	Beibei, Chongqing
33	Cli1	Taichui rough lemon	*C. limon*	mature fruit peel (flavedo)	Domesticated citrus	Beibei, Chongqing
34	Cli2	Eureka lemon	*C. limon*	mature fruit peel (flavedo)	Domesticated citrus	Beibei, Chongqing
35	Cau1	Kesai lime	*C. aurantifolia*	mature fruit peel (flavedo)	Domesticated citrus	Beibei, Chongqing
36	Cme1	Yuan citron	*C. medica*	mature fruit peel (flavedo)	Domesticated citrus	Beibei, Chongqing
37	Cme2	Danna citron	*C. medica*	mature fruit peel (flavedo)	Domesticated citrus	Beibei, Chongqing
38	Cme3	Muli citron	*C. medica*	mature fruit peel (flavedo)	Domesticated citrus	Beibei, Chongqing

Supplemental Table 2. Information of UGTs from different families used for phylogenetic analysis.

Group	Sequence UGTs	Genus/species
A	UGT79A1:CAA81057	*Petunia x hybrida*
	UGT79B1:BAA97127	*Arabidopsis thaliana*
	UGT79C1:XP_025877288.1	*Oryza sativa japonica group*
	UGT91A1:AAD15567	*Arabidopsis thaliana*
	UGT91B1:BAA98174	*Arabidopsis thaliana*
	UGT91C1:BAA98157	*Arabidopsis thaliana*
B	UGT89C1:AAF80123	*Arabidopsis thaliana*
C	UGT90A1:AAC64220	*Arabidopsis thaliana*
D	UGT73B23:XP_004304022.1	*Fragaria vesca subsp. vesca*
	UGT73C6:AAD20155	*Arabidopsis thaliana*
E	UGT71C5:AAG48783	*Arabidopsis thaliana*
	UGT71W2:XP_011468178.1	*Fragaria vesca subsp. vesca*
	UGT88F1:ACZ44840	*Malus x domestica*
	UGT88A1:BAB01151	*Arabidopsis thaliana*
	UGT72B2:NP_171649	*Arabidopsis thaliana*
F	UGT78D1:AAF19756	*Arabidopsis thaliana*
G	UGT85A1:AAF18537	*Arabidopsis thaliana*
	UGT85A2:BAA34687	*Arabidopsis thaliana*
H	UGT76F1:CAB81596	*Arabidopsis thaliana*
	UGT76C2:BAB10791	*Arabidopsis thaliana*
I	UGT83A1:AAF14850	*Arabidopsis thaliana*
J	UGT87A2:AAC16958	*Arabidopsis thaliana*
K	UGT86A1:AAD31582	*Arabidopsis thaliana*
L	UGT74E2:AAD30627	*Arabidopsis thaliana*
	UGT75A1:BAA19155	*Nicotiana tabacum*
M	UGT92A1:CAB88253	*Arabidopsis thaliana*
N	UGT82A1:BAB01943	*Arabidopsis thaliana*
O	UGT93A1:AAD51778	*Phaseolus vulgarus*
	UGT93B2:XP_015633949.1	*Oryza sativa japonica group*
P	UGT709A1:XP_015611418.1	*Oryza sativa japonica group*
Q	UGT95A1:ACB56927	*Hieracium pilosella*

	UGT95B6:XP_010664783.1	*Vitis vinifera*
R	UGT708A1:EAZ00578	*Oryza sativa indica group*
	UGT708A7:KQK18571	*Brachypodium distachyon*
OG	UGT80A2:NP_566297	*Arabidopsis thaliana*
	UGT80A5:XP_015647949.1	*Oryza sativa japonica group*
	UGT81A1:AAP68329	*Arabidopsis thaliana*

Supplemental Table 3. Functionally validated plant dGlyTs used in phylogenetic analysis.

Name	Function	Species	Order	Accession No.
Ph1,6RhaT (UGT79A1)	Anthocyanidin 3-*O*-glucoside 6''-*O*-rhamnosyltransferase	*Petunia × hybrida*	*Solanales*	CAA81057
At1,2XylT (UGT79B1)	Anthocyanin 3-*O*-glucoside 2''-*O*-xylosyltransferase	*Arabidopsis thaliana*	*Brassicales*	NM_124785
At1,2GlcT (UGT79B6)	Flavonoid 3-*O*-glucoside 2''-*O*-glucosyltransferase	*Arabidopsis thaliana*	*Brassicales*	NM_124780
Ip1,2GlcT (UGT79B16)	Anthocyanidin 3-*O*-glucoside 2''-*O*-glucosyltransferase	*Ipomoea purpurea*	*Solanales*	AB192315
Bp1,2GAT (UGT94B1)	Anthocyanin 3-*O*-glucoside 2''-*O*-glucuronosyltransferase	*Bellis perennis*	*Asterales*	AB190262
Si1,6GlcT (UGT94D1)	Sesaminol 2'-*O*-glucoside 6''-*O*-glucosyltransferase	*Sesamum indicum*	*Lamiales*	BAF99027
Si1,2GlcT (UGT94AG1)	Sesaminol 2'-*O*-glucoside 2''-*O*-glucosyltransferase	*Sesamum indicum*	*Lamiales*	LC484013
Cr1,6GlcT	Flavonol and flavone glucosides 6''-*O*-glucosyltransferase	*Catharanthus roseus*	*Gentianales*	AB443870
Gm1,6RhaT (UGT79A6)	Flavonol 3-*O*-glucoside 6''-*O*-rhamnosyltransferase	*Glycine max*	*Fabales*	AB828193
Gm1,2GlcT (UGT79B30)	Flavonol 3-*O*-glucoside 2''-O-glucosyltransferase	*Glycine max*	*Fabales*	LC017844
Gm1,6GlcT (UGT79A7)	Flavonol 3-*O*-glucoside/galactoside 6''-O-glucosyltransferase	*Glycine max*	*Fabales*	LC126028
Fe1,6RhaT (UGT79A8)	Flavonol 3-*O*-glucoside 6''-O-rhamnosyltransferase	*Fagopyrum esculentum*	*Caryophyllales*	LC312144
Cs1,2RhaT (UGT79B28)	Flavonoid 7-*O*-glucoside 2''-O-rhamnosyltransferase	*Camellia sinensis*	*Parietales*	KP682353
Ci1,6RhaT	Flavonoid 3/7-*O*-glucoside 6''-O-rhamnosyltransferase	*Chrysanthemum indicum*	*Asterales*	OL422134
Pp1,6GlcT-1 (UGT73AH1)	Sterol 3-*O*-glucoside 6''-O-glucosyltransferase	*Paris polyphylla*	*Liliales*	OP198197
Pp1,6GlcT-2 (UGT73AH2)	Sterol 3-*O*-glucoside 6''-O-glucosyltransferase	*Paris polyphylla*	*Liliales*	OP198198
Pp1,6GlcT-3 (UGT73AH3)	Sterol 3-*O*-glucoside 6''-O-glucosyltransferase	*Paris polyphylla*	*Liliales*	OP651003
Cm1,2RhaT (UGT94BU1)	Flavanone 7-*O*-glucoside 2''-*O*-rhamnosyltransferase	*Citrus grandis*	*Sapindales*	AY048882
Cs1,6RhaT (UGT79V2)	Flavonoid 3/7-O-glucoside 6''-*O*-rhamnosyltransferase	*Citrus sinensis*	*Sapindales*	DQ119035
Cm1,6RhaT (UGT79V3)	Flavonoid 3/7-O-glucoside 6''-*O*-rhamnosyltransferase	*Citrus grandis*	*Sapindales*	LC057678
CmdGlcT-1 (UGT94BU2)	Flavonoid 3/7-O-glucoside 6''-O-glucosyltransferase	*Citrus grandis*	*Sapindales*	-
CmdGlcT-2 (UGT94BU3)	Flavonoid 3/7-O-glucoside 6''-O-glucosyltransferase	*Citrus grandis*	*Sapindales*	-

CmdGlcT-3 (UGT94BU4)	Flavonoid 3/7-O-glucoside 6"-O-glucosyltransferase	*Citrus grandis*	*Sapindales*	-
Cg2g042800 (UGT79B202)	Flavonoid 3/7-O-glucoside 2"-*O*-rhamnosyltransferase	*Citrus grandis*	*Sapindales*	-
Cg2g042810 (UGT79B203)	Flavonoid 3/7-O-glucoside 2"-O-glucosyltransferase	*Citrus grandis*	*Sapindales*	-
Cg5g041670 (UGT91BK3)	Flavanone 7-O-glucoside 6"-O-rhamnosyltransferase	*Citrus grandis*	*Sapindales*	-

Supplemental Table 4. Functional characterization of group A genes verified in this study against different substrates.

Protein	N7Glu	H7Glu	Q3Glu	Q7Glu	S3Glu	S7Glu	S4'Glu	A7Glu	D7Glu
Cg1g023820 (Cm1,2RhaT)	+	+	-	-	-	-	-	+	+
Cg8g011410	-	-	-	-	-	-	-	-	-
Cg8g018000	-	-	-	-	-	-	-	-	-
Cg8g018010	-	-	-	-	-	-	-	-	-
Cg2g042810 (UGT79B203)	+	+	+	+	+	+	+	+	+
Cg2g042800 (UGT79B202)	-	-	+	+	+	+	+	-	-
Cg5g041670 (UGT91BK3)	+	+	-	-	-	-	-	-	-
Cg4g008340	-	-	-	-	-	-	-	-	-
Cg4g008360	-	-	-	-	-	-	-	-	-
Cg2g035460	-	-	-	-	-	-	-	-	-
Cg1g007740	-	-	-	-	-	-	-	-	-
Cg4g010340	-	-	-	-	-	-	-	-	-
**Cg9g014500**	-	-	-	-	-	-	-	-	-

“+” indicates detectable catalytic activity, while “–” denotes no observed activity. Putative homologs of Cs1,6RhaT are highlighted in bold. Cm1,2RhaT, GenBank accession No. AY048882.

Supplemental Table 5. The conversion rate of citrus dGlyTs and their mutants in this study.

Protein and sugar donor	N7Glu	H7Glu	Q3Glu	Q7Glu	K3Glu	K7Glu	K4'Glu	A7Glu	D7Glu
Cm1,2RhaT (UDP-Rha)	54.98±0.89 a	42.98±0.88 b	0.00	0.00	0.00	0.00	0.00	44.24±1.17 b	47.36±1.67 b
F195M (UDP-Rha)	56.40±0.49 a	59.33±1.39 a	0.00	0.00	0.00	0.00	0.00	58.91±1.27 a	62.08±1.09 a
F195W (UDP-Rha)	5.72±0.19 c	6.46±0.23 d	0.00	0.00	0.00	0.00	0.00	34.24±0.20 c	42.80±4.89 b
F195N (UDP-Rha)	4.48±0.4 cd	4.30±0.08 de	0.00	0.00	0.00	0.00	0.00	5.60±0.72 e	5.14±0.61 c
F195T (UDP-Rha)	2.75±0.18 d	3.6±0.06 e	0.00	0.00	0.00	0.00	0.00	6.97±0.62 de	9.55±0.84 c
F195G (UDP-Rha)	10.73±0.37 b	11.02±0.33 c	0.00	0.00	0.00	0.00	0.00	10.74±0.19 d	10.83±0.83 c
F195L (UDP-Rha)	0.00	0.00	0.00	0.00	0.00	0.00	0.00	0.00	0.00
F195P (UDP-Rha)	0.00	0.00	0.00	0.00	0.00	0.00	0.00	0.00	0.00
Cs1,6RhaT (UDP-Rha)	85.08±3.95 a	98.77±1.74 a	94.30±0.54 a	91.98±0.98 a	99.04±0.41 a	81.55±1.58 b	98.91±0.19 a	81.41±0.71 a	75.64±3.79 a
G216F (UDP-Rha)	0.00	0.00	0.00	0.00	0.00	0.00	0.00	0.00	0.00
G216T (UDP-Rha)	75.42±0.31 b	92.91±1.02 b	60.93±1.70 b	72.28±1.25 b	87.55±0.09 b	84.03±1.58 a	70.17±0.48 b	45.81±5.53 b	39.37±2.09 b
UGT91BK3 (UDP-Rha)	2.23±0.07 b	2.34±0.49 b	0.00	0.00	0.00	0.00	0.00	0.00	0.00
T216F (UDP-Rha)	4.02±0.20 b	4.11±0.12 b	13.63±0.25 a	1.17±0.30 a	17.18±0.43 a	14.31±0.63 a	18.98±1.04 a	8.97±0.38 a	11.09±0.27 a
T216G (UDP-Rha)	20.61±0.75 a	20.31±1.65 a	0.22±0.01 b	0.91±0.15 b	0.14±0.01 b	0.24±0.03 b	0.15±0.04 b	0.53±0.06 b	0.49±0.05 b
UGT79B202 (UDP-Glc)	0.00	0.00	0.34±0.04	0.67±0.23 a	2.47±0.42 b	0.00	2.00±0.14 c	0.00	0.00
N202F (UDP-Glc)	0.00	0.00	2.36±0.13 b	0.43±0.04 b	4.25±0.19 a	0.00	3.70±0.05 b	0.00	0.00
N202L (UDP-Glc)	0.00	0.00	6.13±0.12 a	0.00	4.28±0.09 a	0.00	5.19±0.19 a	0.00	0.00
UGT79B203 (UDP-Glc)	0.55±0.10 b	0.59±0.07 a	94.34±0.58 a	2.51±0.09 b	99.57±0.08 a	1.17±0.20 a	99.73±0.38 a	0.99±0.02 a	2.12±0.26 ab
L201F (UDP-Glc)	0.00	0.89±0.20 a	23.29±2.56 b	0.56±0.07 c	69.47±0.85 b	0.28±0.05 b	86.24±0.63 b	0.00	0.68±0.10 b
L201N (UDP-Glc)	0.78±0.09 a	0.69±0.09 a	87.35±3.34 a	6.19±0.55 a	99.26±0.24 a	1.32±0.16 a	99.64±0.11 a	0.36±0.02 b	3.78±0.64 a

Data represent mean ± SD (n = 3). Statistical analysis was performed using one-way ANOVA (Tukey's test) for multiple groups and Student's t-test for two-group comparisons. Different letters indicate significant differences at $P < 0.01$.

Supplemental Table 6. Information of standards used in this study.

Experiment	Name	Abbreviation	Molecular formula	CAS number
Substrate of functional verification	Naringenin 7-*O*-glucoside	N7Glu	$C_{21}H_{22}O_{10}$	529-55-5
	Hesperetin 7-*O*-glucoside	H7Glu	$C_{22}H_{24}O_{11}$	31712-49-9
	Quercetin 3-*O*-glucoside	Q3Glu	$C_{21}H_{20}O_{12}$	482-35-9
	Quercetin 7-*O*-glucoside	Q7Glu	$C_{21}H_{20}O_{12}$	491-50-9
	Kaempferol 3-*O*-glucoside	K3Glu	$C_{21}H_{20}O_{11}$	480-10-4
	Kaempferol 7-*O*-glucoside	K7Glu	$C_{21}H_{20}O_{11}$	16290-07-6
	Kaempferol 4'-*O*-glucoside	K4'Glu	$C_{21}H_{20}O_{11}$	52222-74-9
	Apigenin 7-*O*-glucoside	A7Glu	$C_{21}H_{20}O_{10}$	578-74-5
	Diosmetin-7-*O*-glucoside	D7Glu	$C_{22}H_{22}O_{11}$	20126-59-4
	Naringenin	Nar	$C_{15}H_{12}O_{5}$	480-41-1
	Hesperitin	Hes	$C_{16}H_{14}O_{6}$	520-33-2
Sugar donor of functional verification	UDP-glucose	UDP-Glc	$C_{15}H_{22}N_{2}Na_{2}O_{17}P_{2}$	28053-08-9
	UDP-rhamnose	UDP-Rha	$C_{15}H_{24}N_{2}O_{16}P_{2}$	1955-26-6
	UDP-xylose	UDP-Xyl	$C_{14}H_{23}N_{2}NaO_{16}P_{2}$	108320-89-4
	UDP-galactose	UDP-Gal	$C_{15}H_{24}N_{2}O_{17}P_{2}$	2956-16-3
Product of functional verification	Naringenin 7-*O*-neohesperidoside	N7Neo	$C_{27}H_{32}O_{14}$	10236-47-2
	Hesperetin 7-*O*-neohesperidoside	H7Neo	$C_{28}H_{34}O_{15}$	13241-33-3
	Naringenin 7-*O*-rutinoside	N7Rut	$C_{27}H_{32}O_{14}$	14259-46-2
	Hesperetin 7-*O*-rutinoside	H7Rut	$C_{28}H_{34}O_{15}$	520-26-3
	Quercetin 3-*O*-neohesperidoside	Q3Neo	$C_{27}H_{30}O_{16}$	32453-36-4
	Quercetin 3-*O*-sophoroside	Q3Sop	$C_{27}H_{30}O_{17}$	18609-17-1
	Kaempferol-3-*O*-neohesperidoside	K3Neo	$C_{27}H_{30}O_{15}$	142451-65-8
	Kaempferol-3-*O*-sophoroside	K3Sop	$C_{27}H_{30}O_{16}$	19895-95-5
Internal standard for metabolic analysis	Acyclovir		$C_{8}H_{11}N_{5}O_{3}$	59277-89-3
	Roxithromycin		$C_{41}H_{76}N_{2}O_{15}$	80214-83-1
