# Supplementary material for: Evolutionary origin of neohesperidoside, a bitter metabolite, and its potential role in biotic defense and citrus dissemination
Source: Plant Commun. 2026 Jan 5;7(4):101697. doi: 10.1016/j.xplc.2026.101697 (PMC13084081; doi:10.1016/j.xplc.2026.101697)
Supplement: Document S2. Article plus supplemental information [file mmc3.pdf]

Plant Communications
Research article

CellPress
Partner Journal

Evolutionary origin of neohesperidoside, a bitter metabolite, and its potential role in biotic defense and citrus dissemination

Gu Li[1,2,3,4], Huan Wen[1,2], Hanxin Zhou[1,2], Yuan Liu[1,2], Ziyu Yuan[1,2], Huixian Zhang[1,2], Zhehui Hu[1,2], Zeyang Liu[1,2], Huili Ma[1,2], Qi Chen[1,2], Guixiang Chen[1,2], Jia-Long Yao[3], Juan Xu[1,2,4,5,*] and Jiajing Chen[1,2,4,5,*]

[1]National Key Laboratory for Germplasm Innovation & Utilization of Horticultural Crops, College of Horticulture and Forestry Sciences, Huazhong Agricultural University, Wuhan 430070, P.R. China

[2]Hainan Research Institute of Huazhong Agricultural University, Sanya 572025, P.R. China

[3]New Zealand Institute for Bioeconomy Science Limited, Private Bag 92169, Auckland 1142, New Zealand

[4]Hubei Hongshan Laboratory, Wuhan 430070, P.R. China

[5]Sensory Evaluation and Quality Analysis Centre of Horticultural Products, Huazhong Agricultural University, Wuhan 430070, P.R. China

***Correspondence: Juan Xu (xujuan@mail.hzau.edu.cn), Jiajing Chen (chenjiajing@mail.hzau.edu.cn)**

https://doi.org/10.1016/j.xplc.2026.101697

ABSTRACT

Fruit secondary metabolites play pivotal roles in plant evolution by deterring herbivores and attracting seed dispersers. However, the mechanisms by which these compounds evolve and drive diversification in citrus remain poorly understood. In this study, we demonstrate that the emergence of the bitter compound neohesperidoside (Neo) has contributed to citrus dissemination by enhancing defense against biotic stresses. Targeted metabolomic analyses revealed that Neo accumulation emerged in early-diverging citrus lineages, whereas its non-bitter counterpart rutinoside (Rut) can be traced back to *Citrus*-related species. Comparative genomic analyses and enzyme functional assays further revealed that Neo biosynthesis originated from the duplication of two di-glucosyltransferase genes, *CmdGlcT-1* and *UGT79B203*, in early-diverging citrus, followed by neofunctionalization into Cm1,2RhaT and UGT79B202, enzymes capable of synthesizing Neo. A structurally conserved amino acid residue—corresponding to Phe195 in Cm1,2RhaT and Leu201 in UGT79B203—was identified as critical for this functional transition. Compared with Rut, Neo exhibits stronger antifungal and anti-feeding activities, suggesting a role in enhanced biotic defense that may have contributed to the broader geographical distribution of early-diverging citrus species. Together, these findings provide new insights into the evolutionary origin of citrus bitterness and highlight the adaptive role of specialized metabolites in shaping plant–environment interactions.

Key words: citrus, bitterness, neohesperidoside, disaccharide glycosyltransferases, neofunctionalization, biotic defense

Li G., Wen H., Zhou H., Liu Y., Yuan Z., Zhang H., Hu Z., Liu Z., Ma H., Chen Q., Chen G., Yao J.-L., Xu J., and Chen J. (2026). Evolutionary origin of neohesperidoside, a bitter metabolite, and its potential role in biotic defense and citrus dissemination. Plant Comm. **7**, 101697.

INTRODUCTION

Flavonoids are phenolic secondary metabolites that have evolved in plants as adaptive responses to environmental challenges such as ultraviolet (UV) radiation, herbivory, pathogen attacks, and the need to attract pollinators (Tanaka et al., 2008; Cho and Lee, 2015; Peng et al., 2017; Trunschke et al., 2021; Forster et al., 2022; Sugimoto et al., 2022). In plants, flavonoids often accumulate as glycosides, which can be classified into monoglycosides and diglycosides (Shen et al., 2022). The latter are further categorized based on sugar composition and glycosidic linkage, with common types including neohesperidoside (Neo), rutinoside (Rut), gentiobioside (Gen), robinobioside (Rob), sophoroside (Sop), sambubioside (Sam), and apiosylrhamnoside (Api). Citrus species are particularly known for the accumulation of flavonoid disaccharide glycosides, represented by the bitter Neo, such as naringin, or the bitterless Rut, such as hesperidin (Li et al., 2022). Genomic analyses have

This is an open access article under the CC BY license (http://creativecommons.org/licenses/by/4.0/).

categorized the orange subfamily (Rutaceae: Aurantioideae) into three major groups: *Citrus*-related genera, early-diverging citrus, and domesticated citrus (Huang et al., 2023). The *Citrus*-related genera group, including *Atalantia buxifolia*, was previously considered to represent primitive citrus species (Huang et al., 2018). The early-diverging citrus group contains wild species such as *Citrus trifoliata*, *Citrus mangshanensis,* and *Citrus ichangensis*. The domesticated citrus group experienced a complex history of admixture, and most modern citrus cultivars are derived from three basic species, *Citrus grandis* (pummelo), *Citrus reticulata* (mandarin), and *Citrus medica* (citron) (Wu et al., 2018). Neo compounds, which contribute to the primary bitterness of citrus fruits, accumulate at high levels in pummelo but are largely absent in mandarin and citron (Frydman et al., 2004; Chen et al., 2019; Li et al., 2022). This gain and loss of Neo biosynthetic capacity during citrus evolution makes citrus an excellent model for investigating the evolution and domestication of flavonoid-derived bitterness.

Neo is synthesized through rhamnosylation of flavonoid 7-*O*-glucosides, a reaction that elongates the sugar chain and often imparts distinct biological functions and flavor properties to flavonoids. For example, Neo formation results in a pronounced bitter taste, whereas 2″-*O*-rhamnosylation of sterol 3-*O*-glucosides (e.g., Polyphyllin I and Polyphyllin H) has been linked to enhanced antifungal activity in *Paris polyphylla* (Chen et al., 2023). These rhamnosylation reactions are predominantly catalyzed by UDP-dependent glycosyltransferases (UGTs), particularly a subgroup known as disaccharide-forming UGTs (dGlyTs), which are phylogenetically distinct from monosaccharide UGTs (Wilson and Tian, 2019). In pummelo, 1,2-rhamnosyltransferase (Cm1,2RhaT) has been reported to catalyze the formation of Neo from flavanone and flavone substrates (Frydman et al., 2004). Additionally, the related di-glucosyltransferase CmdGlcT-1, which shares a common evolutionary origin with Cm1,2RhaT, exhibits distinct sugar-donor specificity and catalyzes the formation of glucosyl–glucoside disaccharides (Chen et al., 2019). Previous studies suggested that artificial selection against bitterness contributed to the loss of the *1,2RhaT* gene in sweet oranges (Chen et al., 2019). However, the genetic basis underlying *1,2RhaT* evolution and bitterness formation in citrus remains unclear.

Amino acid substitutions in enzymes can give rise to novel enzymatic activities, leading to the emergence of new specialized metabolites. In glycosyltransferases, single-amino-acid changes have been shown to alter sugar-donor or sugar-acceptor specificity, thereby expanding the diversity of glycosylated metabolites (Chen and Li, 2016; Zhang et al., 2021). When such metabolic innovations confer ecological advantages, such as enhanced defense or increased stress tolerance, they may be favored by natural selection, leading to the fixation of relevant genetic variants in plant populations (Noda-Garcia et al., 2018). Flavonoids are generally associated with antioxidant activity and protection against temperature stress, whereas bitter compounds frequently deter herbivores and pests (Thodberg et al., 2018; Gouot et al., 2019; Wang et al., 2022). Despite extensive research on Neo biosynthesis and accumulation across citrus species, the ecological functions of Neo in shaping interactions between citrus and their environment remain largely unexplored. Investigating how Neo influences plant–environment interactions could therefore provide valuable insights into the selective pressures driving the evolution of specific plant metabolites.

Here, we performed targeted metabolomic analyses on 38 accessions representing *Citrus*-related genera, early-diverging citrus, and domesticated citrus. We found that the accumulation of bitter Neo originated in early-diverging citrus species. Enzyme functional validation combined with comparative genomic analyses revealed that duplication and neofunctionalization of two di-glucosyltransferase genes in early-diverging citrus enabled the transition from 2″-*O*-glucosylation to 2″-*O*-rhamnosylation, thereby facilitating Neo biosynthesis. A key amino acid residue was identified as critical for this neofunctionalization. Furthermore, bitter Neo exhibited stronger antifungal and antiherbivory effects than its Rut counterpart. Given the broader geographical distribution of early-diverging citrus compared to *Citrus*-related genera, we propose that Neo may have contributed to adaptive evolution by enhancing defense against biotic stresses. Overall, this study provides new insights into the evolution of citrus bitterness and highlights the role of specialized metabolites in mediating plant–environment interactions.

RESULTS

Accumulation of bitter neohesperidoside began in early-diverging citrus species

To characterize flavonoid variation during citrus evolution, a widely targeted metabolomic analysis was performed using four accessions of *Citrus*-related species (*A. buxifolia* and *Clausena lansium*), 10 accessions of early-diverging citrus species (*C. trifoliata*, *C. ichangensis*, and *C. mangshanensis*), and 24 accessions representing six domesticated citrus species (*C. grandis*, *C. reticulata*, *C. medica*, *Citrus aurantium*, *Citrus sinensis*, and *Citrus limon*) (Figure 1A; Supplemental Table 1). This analysis identified 67 flavonoid compounds, including seven aglycones, 21 monoglycosides, and 39 disaccharide glycosides (Supplemental Data 1). The results showed that *A. buxifolia* accumulated predominantly monoglycoside flavonoids, whereas citrus species accumulated mainly disaccharide glycosides and showed higher total flavonoid levels (Supplemental Figure 1). Heatmap analysis further revealed that the accumulation of these glycoside types displayed germplasm-specific patterns (Supplemental Figure 2).

We then examined the distribution and abundance of seven disaccharide glycoside types across the surveyed species. Neo and/or Rut were the predominant disaccharide glycosides. Rut was already present in *Citrus*-related species, whereas the accumulation of the bitter Neo emerged in early-diverging citrus species and was associated with a marked increase in total disaccharide glycoside content (Figure 1B; Supplemental Data 2). In domesticated citrus, the accumulation of Neo and Rut was species dependent. Neo was more abundant in pummelo (*C. grandis*), whereas Rut predominated in mandarin-derived species (*C. reticulata*, *C. sinensis*, and *C. limon*), as well as in citron (*C. medica*). Notably, sour orange (*C. aurantium*), a mandarin–pummelo hybrid, accumulated high levels of both Neo and Rut. In contrast, the remaining disaccharide glycoside types were consistently detected at low abundance across citrus species. Together, these findings indicate that Neo

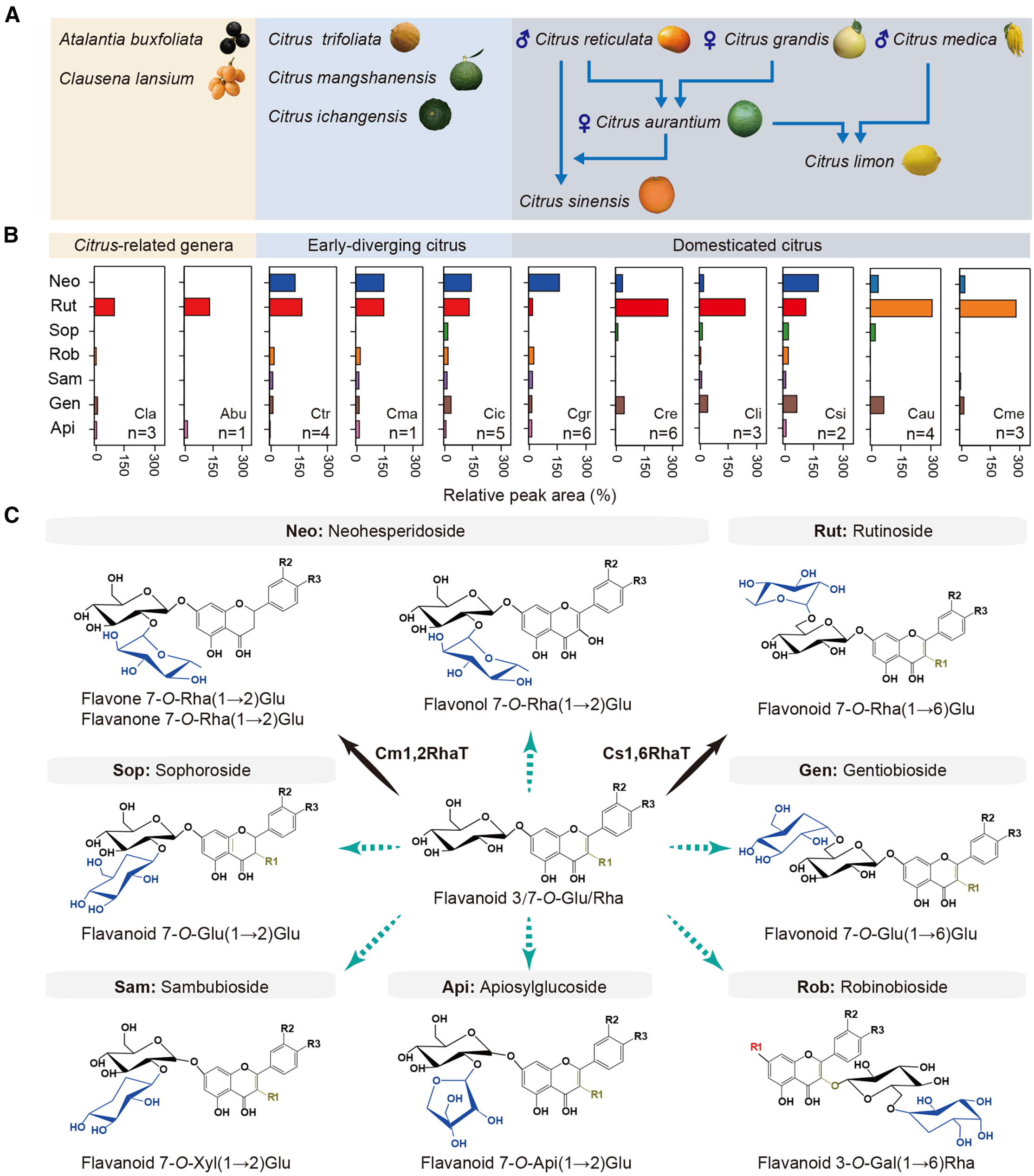

Figure 1. Flavonoid disaccharide glycoside metabolome among different citrus and *Citrus*-related species.
(A) Phylogenetic relationships of the species analyzed in this study. *Clausena lansium* (Cla) and *Atalantia buxifolia* (Abu) belong to *Citrus*-related genera; *Citrus trifoliata* (Ctr), *C. ichangensis* (Cic), and *C. mangshanensis* (Cma) represent early-diverging citrus; *C. grandis* (Cgr), *C. reticulata* (Cre), *C. medica* (Cme), *C. aurantium* (Cau), *C. sinensis* (Csi), and *C. limon* (Cli) represent domesticated citrus.
(B) Levels of seven flavonoid disaccharide glycosides in fruit flavedo from different species. Relative levels are presented as means of three biological replicates based on log2-transformed ion pair peak areas. "n" represents the number of accessions analyzed for each species. Neo, neohesperidosides; Rut, rutinosides; Sop, sophorosides; Rob, robinobiosides; Sam, sambubiosides; Gen, gentiobiosides; Api, apiosides.
(C) Molecular structures of seven representative disaccharide glycosides detected across species. Solid lines indicate that the corresponding biosynthetic enzyme has been characterized in citrus; dashed lines indicate that the relevant enzyme has not yet been characterized.

accumulation originated and became fixed in early-diverging citrus lineages, representing a key metabolic innovation during citrus evolution.

Structural analysis of the identified disaccharide glycosides revealed clear patterns in sugar linkage types and composition. Gen and Rob, similar to Rut, are characterized by β1–6 glycosidic linkages; however, Gen contains a glucosyl–glucose moiety, whereas Rob harbors a galactosyl–rhamnose moiety. The remaining three disaccharide glycosides are connected via β1–2 linkages, similar to Neo, but differ in their sugar composition. Specifically, Sop, Sam, and Api incorporate glucosyl, xylosyl, and apiosyl moieties, respectively, as the second sugar attached to the flavonoid glucoside acceptor (Figure 1C).

Among these glycosides, Neo is synthesized by 1,2RhaT and Rut by 1,6RhaT, whereas the enzymes responsible for synthesizing the remaining disaccharides have not yet been identified. The previously characterized 1,2RhaT (Cm1,2RhaT; accession no. AY048882) was reported to lack activity toward flavonol substrates (Frydman et al., 2004). However, the detection of flavonol neohesperidosides—such as isorhamnetin 3-*O*-neohesperidoside—in pummelo and sour orange suggests the existence of a distinct 1,2RhaT capable of catalyzing neohesperidoside formation in citrus (Supplemental Data 1).

Two pairs of paralogous *dGlyT* genes are associated with neohesperidoside biosynthesis in citrus

To identify dGlyTs potentially involved in citrus Neo metabolism, we performed a phylogenetic analysis of UGT proteins identified from the reference genomes of eight *Citrus* and *Citrus*-related species. Among these species, *C. grandis* contained the largest number of UGTs, with 17 members belonging to phylogenetic group A, which represents the main branch of dGlyTs (Figures 2A and 2B). Of these, 13 genes possessed intact open reading frames in *C. grandis* Wanbaiyou, whereas the remaining four genes—including a homolog of *Cs1,6RhaT* (accession no. DQ119035)—were prematurely terminated due to frameshift mutations (Supplemental Data 3). Recombinant proteins corresponding to the 13 intact *UGTs* were heterologously expressed in *Escherichia coli* and purified for subsequent functional characterization.

Enzymatic activities of the recombinant proteins were assessed using nine flavonoid glucosides (including flavanones, flavonols, and flavones) as acceptor substrates, together with UDP-Glc, UDP-Rha, UDP-Xyl, and UDP-Gal as sugar donors (Supplemental Figure 3). This screening identified three novel UGTs with dGlyT activity. UGT79B202 (Cg2g042800) exhibited dual 1,2RhaT and 1,2GlcT activities, catalyzing 2″-glycosylation and 2″-rhamnosylation of Q3Glu and K3Glu to produce the corresponding Sop and Neo, respectively (Figure 2C). UGT79B203 (Cg2g042810) was identified as a 1,2GlcT, catalyzing 2″-glycosylation of Q3Glu and K3Glu to generate Sop (Figure 2D). In addition, UGT91BK3 (Cg5g041670) catalyzed 1,6-rhamnosylation of N7Glu and H7Glu to generate the corresponding Rut compounds (Figure 2E).

Substrate specificities of UGT79B202, UGT79B203, and UGT91BK3 were further evaluated alongside Cm1,2RhaT and Cs1,6RhaT. Among enzymes catalyzing β1–2 glycosidic linkages, Cm1,2RhaT demonstrated strong activity toward flavanone and flavone substrates, with conversion rates ranging from 42.98% to 54.98%, but showed no detectable activity toward flavonol substrates (Figure 2F; Supplemental Table 4). In contrast, UGT79B202 exhibited strict substrate specificity for flavonols, albeit with modest conversion rates (0.34%–2.47%) (Figure 2F; Supplemental Figures 4 and 5; Supplemental Table 4). Both Cm1,2RhaT and UGT79B202 catalyzed Neo formation but exhibited complementary substrate preferences. UGT79B203 showed strong activity in converting flavonol glucosides (Q3Glu, K3Glu, and K4′Glu) into their corresponding sophorosides, with conversion rates of 94.34%–99.57%, but exhibited only weak activity toward flavanones and flavones (0.55%–2.51%) (Figure 2F; Supplemental Figures 6 and 7; Supplemental Table 4).

Despite sharing 78.26% amino acid identity and clustering closely in the phylogenetic tree (Figure 2B), UGT79B202 and UGT79B203 differed markedly in sugar-donor specificity. A similar functional divergence was observed between Cm1,2RhaT and CmdGlcT-1, which share 85.84% amino acid identity. These observations suggest that two phylogenetically related yet functionally distinct dGlyT pairs may have coevolved and contributed to the emergence of bitter Neo biosynthesis.

Among enzymes catalyzing β1 → 6 glycosidic linkages, Cs1,6RhaT demonstrated broad substrate promiscuity, catalyzing all tested substrates with high conversion rates (75.64%–99.04%). In contrast, UGT91BK3 showed weak activity that was restricted to flavanone substrates, with conversion rates of only 2.23%–2.34% (Figure 2G; Supplemental Figure 8; Supplemental Table 4). Although both enzymes catalyze Rut biosynthesis, they share only 30.66% amino acid sequence identity, indicating that they function as distinct isoenzymes acting in concert.

Finally, sugar-donor preferences of UGT79B202 and UGT79B203 were examined using their optimal acceptor substrates. Although UGT79B202 showed comparable conversion rates with UDP-Rha and UDP-Glc in endpoint assays (Figure 2H), kinetic analyses revealed a 4.9-fold higher catalytic efficiency, measured as k_{cat}/K_m, for UDP-Rha compared with UDP-Glc (Supplemental Figure 9). In addition, UGT79B203 exhibited trace activity toward UDP-Gal, corresponding to 2.17% of its activity with UDP-Glc (Figure 2H), whereas no detectable activity toward alternative sugar donors was observed for the remaining dGlyTs.

Duplication and functional diversification of *dGlyT* genes in early-diverging citrus led to the production of bitter neohesperidosides

Total flavonoid content was higher in early-diverging and domesticated citrus species than in *Citrus*-related species (Supplemental Figure 1). Therefore, we conducted a comparative genomic analysis across the Rutaceae family. This analysis revealed that *C. trifoliata*, an early-diverging citrus species, has experienced gene family expansions relative to its ancestors, including expansions in families associated with flavonoid biosynthetic pathways. These genomic changes are consistent with the observed diversification of flavonoid metabolism (Figure 3A).

To investigate the evolutionary trajectories of the two pairs of paralogous *dGlyT* genes associated with Neo biosynthesis, we conducted a collinearity analysis across multiple taxa, including *Citrus*-related genera (*C. lansium*, *Luvunga scandens*, *A. buxifolia*), early-diverging citrus (*C. trifoliata*, *C. mangshanensis*, *C. ichangensis*), and domesticated citrus (*C. grandis*, *C. aurantium*, *C. sinensis*, *Citrus clementina*) (Figure 3B; Supplemental Data 4). This analysis showed that *dGlcT-1* and *UGT79B203*, which encode UDP-Glc-utilizing enzymes responsible for Sop biosynthesis, are present in the genomes of *Citrus*-related genera. In contrast, *1,2RhaT* and *UGT79B202*, which encode UDP-Rha-utilizing enzymes that catalyze Neo formation, first appeared in the genomes of early-diverging citrus species (Figure 3B). Duplication analysis further indicated that *1,2RhaT* arose through a dispersed duplication of *dGlcT-1*, whereas *UGT79B202* originated from a tandem

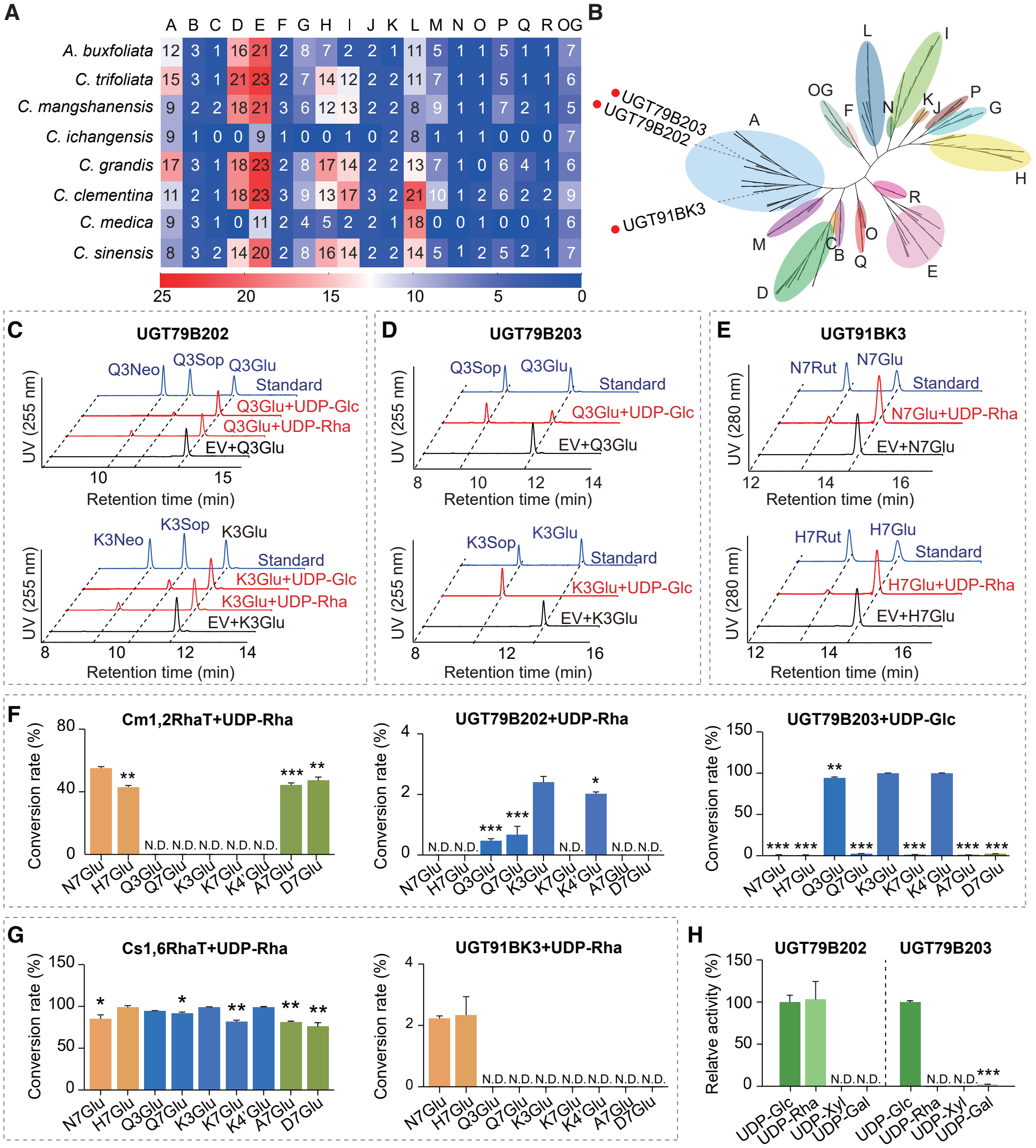

Figure 2. Functional characterization and substrate specificity of citrus dGlyTs.

(A) Heatmap showing the number of UGT genes identified in each UGT phylogenetic group (A–N and the outgroup [OG]) across eight citrus reference genomes.

(B) Phylogenetic tree constructed using protein sequences of putative UGTs identified from the *C. grandis* cv. Wanbaiyou reference genome, showing different UGT groups. Group A contains functionally characterized dGlyTs. The tree was adapted from Wilson and Tian (2019). The three dGlyTs functionally characterized in this study are highlighted with red dots.

(C–E) HPLC elution profiles of compound standards (blue), reaction mixtures catalyzed by recombinant UGT79B202 **(C)**, UGT79B203 **(D)**, and UGT91BK3 **(E)** (red lines), and reaction mixtures catalyzed by recombinant protein from the empty vector (black lines).

(F and G) Substrate specificity of enzymes catalyzing $\beta 1 \rightarrow 2$ linkages **(F)** and $\beta 1 \rightarrow 6$ linkages **(G)** in the disaccharide moiety.

(H) Sugar-donor specificity of UGT79B202 and UGT79B203.

N7Glu, naringenin 7-*O*-glucoside; H7Glu, hesperetin 7-*O*-glucoside; Q3Glu, quercetin 3-*O*-glucoside; Q7Glu, quercetin 7-*O*-glucoside; K3Glu, kaempferol 3-*O*-glucoside; K7Glu, kaempferol 7-*O*-glucoside; K4′Glu, kaempferol 4′-*O*-glucoside; A7Glu, apigenin 7-*O*-glucoside; D7Glu, diosmetin 7-*O*-glucoside; UDP-Glc, UDP-glucose; UDP-Rha, UDP-rhamnose. Error bars represent standard deviations from three biological replicates. Statistical significance was assessed using Student's *t*-test by comparison with the substrate showing the highest conversion rate. *$p < 0.05$, **$p < 0.01$, ***$p < 0.001$.

duplication of *UGT79B203* (Figure 3B). These results suggest that gene duplication followed by neofunctionalization enabled a functional transition from 2″-*O*-glucosylation to 2″-*O*-rhamnosylation. This molecular innovation coincided with the absence of Neo in *Citrus*-related species and its emergence in early-diverging citrus lineages.

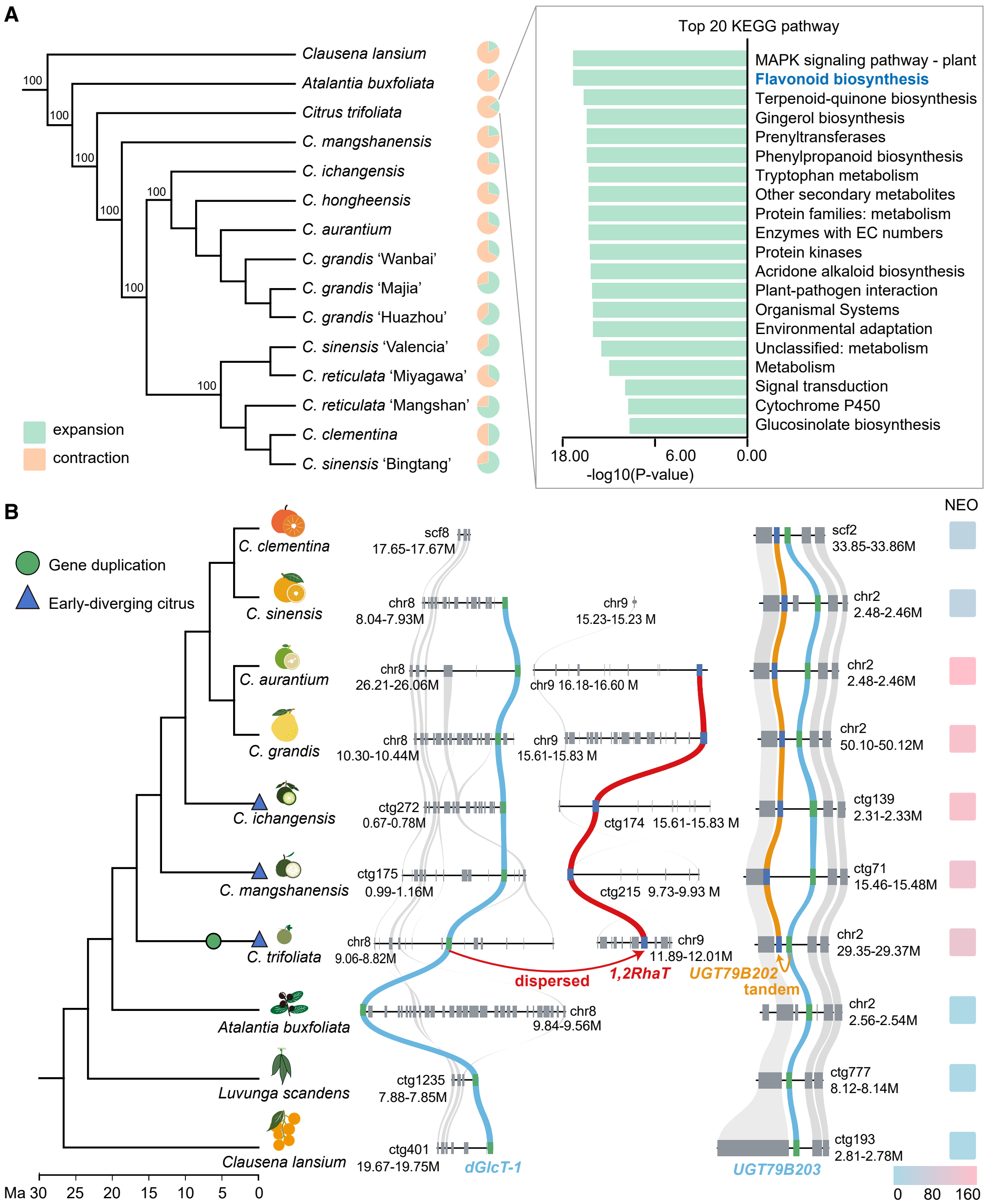

Figure 3. Gene family expansion and evolutionary analysis of two *dGlyT* pairs related to neohesperidoside biosynthesis.

(A) Phylogenetic tree constructed using protein sequences of single-copy orthologous genes identified from reference genomes of 15 *Citrus* and *Citrus*-related species (left panel). Kyoto Encyclopedia of Genes and Genomes (KEGG) enrichment analysis was carried out using gene families expanded in *C. trifoliata* relative to its ancestor (right panel).

(B) Collinearity analysis of two *dGlyT* gene pairs involved in neohesperidoside (Neo) biosynthesis within the Rutaceae family. In *Citrus*-related species, *dGlcT-1* and *UGT79B203* (both utilizing UDP-Glc as the sugar donor) are present, whereas *1,2RhaT* and *UGT79B202* (both utilizing UDP-Rha and catalyzing Neo biosynthesis) are absent. In early-diverging citrus (*C. trifoliata*), dispersed duplication of *dGlcT-1* gave rise to *1,2RhaT*, which exclusively utilizes UDP-Rha. Similarly, tandem duplication of *UGT79B203* generated *UGT79B202*, which exhibits dual activity toward UDP-Glc and UDP-Rha. During this evolutionary phase, duplication and neofunctionalization of *dGlcT-1* and *UGT79B203* produced two rhamnosyltransferases, *1,2RhaT* and *UGT79B202*, thereby enabling Neo formation. The heatmap (right panel) shows Neo accumulation levels across different citrus and citrus-related species.

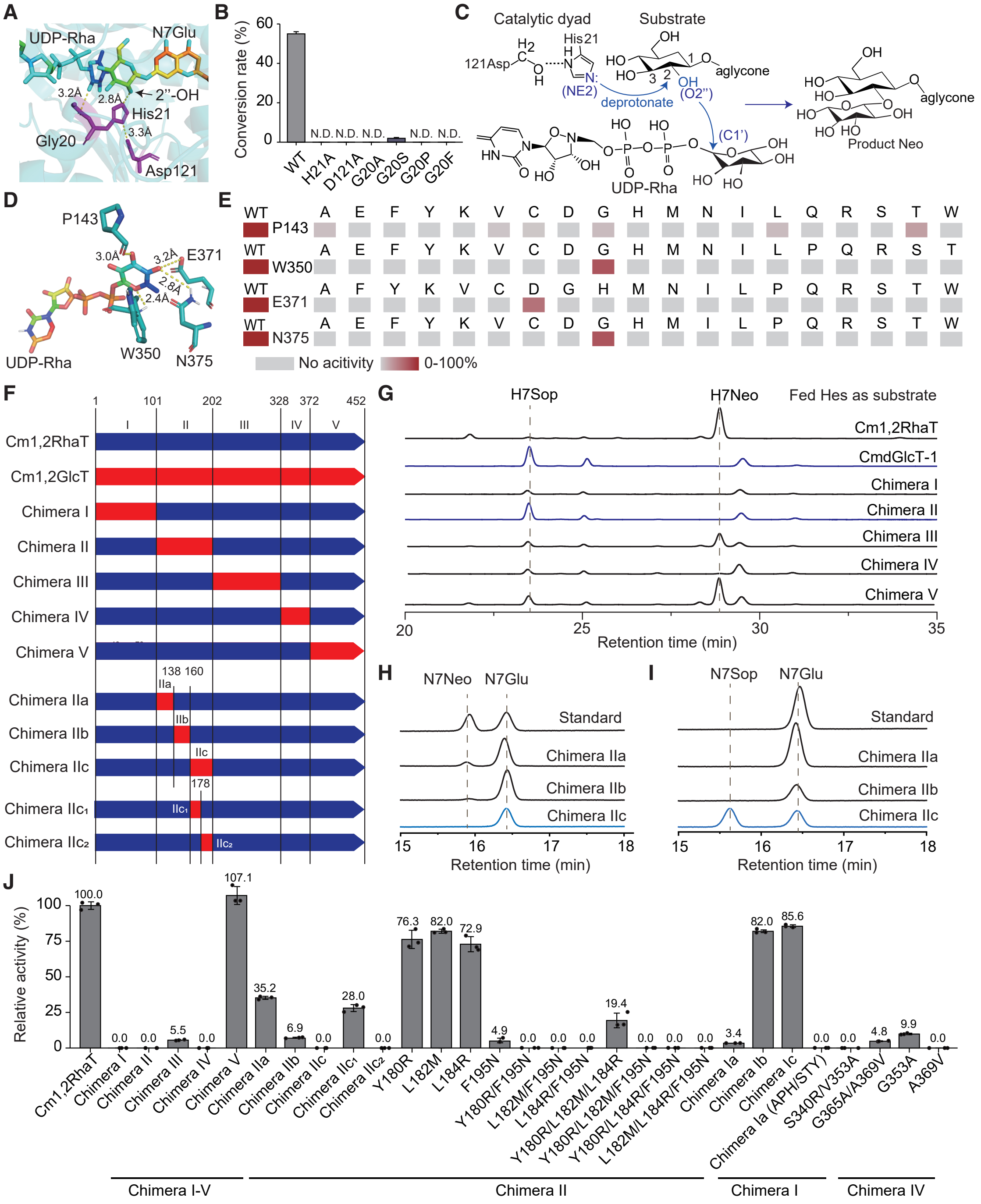

Figure 4. Catalytic mechanism, sugar-donor recognition, and specificity of Cm1,2RhaT.

(A) Docking analysis of Cm1,2RhaT showing key catalytic residues involved in the catalytic mechanism. Yellow dashed lines indicate hydrogen bonds.

(B) Enzyme activity assay of Cm1,2RhaT demonstrating that single-point mutations at key catalytic residues abolish the chemical conversion rate when N7Glu is used as the acceptor and UDP-Rha as the sugar donor.

(C) Proposed S_N2 catalytic mechanism of Cm1,2RhaT. His21 (assisted by Asp121) deprotonates the 2″-hydroxyl group of the glucose moiety attached to the aglycone, creating a nucleophile that initiates an S_N2 attack on the C1′ carbon of UDP-Rha, resulting in neohesperidoside (Neo) formation. Key atoms involved in the reaction are shown in blue.

(D) Positions of four residues that interact with the rhamnose moiety of UDP-Rha.

(E) Heatmap showing functional screening of wild-type (WT) and mutant Cm1,2RhaT using N7Glu as the acceptor and UDP-Rha as the sugar donor. Mutations included replacement of each of the four interacting residues with the remaining 19 amino acids.

(F) Schematic comparison of protein sequences of Cm1,2RhaT, CmdGlcT-1, and the chimeric variants generated in this study. Lines represent region boundaries, and numbers denote corresponding residue positions in the Cm1,2RhaT sequence.

(legend continued on next page)

Collinearity analysis further revealed the absence of syntenic genes corresponding to *1,2RhaT* in mandarin and its derived species, *C. clementina* and *C. sinensis*. To confirm this absence, we analyzed whole-genome re-sequencing data from 135 accessions representing the three basic citrus species. The results showed that *1,2RhaT* is present in all pummelo accessions but absent in citron and most wild mandarin accessions (Supplemental Data 5). The loss of *1,2RhaT* in domesticated citrus may reflect adaptive responses to novel ecological or agronomic environments during domestication. Notably, *1,2RhaT* was detected in several cultivated mandarin accessions, likely due to introgression from pummelo. Consistently, heatmap analysis revealed a strong correlation between Neo accumulation and the presence of *1,2RhaT* across species. Although *C. sinensis* and *C. clementina* both retain *UGT79B202*, they exhibit markedly reduced Neo accumulation, underscoring the indispensable role of *1,2RhaT* in Neo biosynthesis.

In addition, collinearity analysis of other citrus *dGlyTs* revealed that *1,6RhaT* and *UGT91BK3* are present in *Citrus*-related genera and exhibit conserved synteny across multiple species (Supplemental Data 4), consistent with the detection of Rut in *C. lansium* and *A. buxifolia*.

Catalytic mechanism, sugar-donor recognition, and specificity of Cm1,2RhaT

Given the crucial role of 1,2RhaT in Neo biosynthesis, we performed homology modeling and molecular docking analyses to identify key amino acid residues responsible for its catalytic activity and sugar-donor specificity (Supplemental Figure 10).

Structural modeling revealed a catalytic dyad composed of highly conserved His and Asp residues located near the active site. Docking analysis showed that His21 forms hydrogen bonds with both the 2-hydroxyl group of N7Glu and the side chain of Asp121 in Cm1,2RhaT (Figure 4A). Alanine substitution of either His21 or Asp121 completely abolished rhamnosylation activity, confirming the indispensable role of this catalytic dyad in Cm1,2RhaT function (Figure 4B). Additionally, Gly20 forms a hydrogen-bond with the substrate, and substitution at this position resulted in a complete loss of catalytic activity (Figure 4B), suggesting a critical role in stabilizing substrate binding and facilitating proper positioning of His21 within the active site. The catalytic reaction is mediated by the His–Asp dyad via a direct-displacement, S_N2-like pathway (Figure 4C). Briefly, the 2-hydroxyl group (O2″) of the glucose moiety attached to the aglycone forms a hydrogen bond with the nitrogen (NE2) of His21. His21 is, in turn, hydrogen bonded to Asp121, creating the catalytic dyad. His21 acts as a general base to deprotonate O2″, generating a nucleophile that subsequently attacks the C1′ carbon of UDP-Rha, resulting in Neo formation.

In addition, residues Phe143, Glu371, Trp350, and Asn375 were found to interact with the rhamnose moiety of UDP-Rha (Figure 4D), suggesting potential roles in sugar-donor recognition or specificity. To evaluate their functional importance, saturation mutagenesis was performed at each position. Crude protein extracts from the resulting variants were assayed using N7Glu as the acceptor substrate in the presence of four sugar donors: UDP-Rha, UDP-Glc, UDP-Xyl, and UDP-Gal. Nearly all substitutions led to a substantial reduction or complete loss of rhamnosylation activity (Figure 4E). However, no activity toward alternative sugar donors was detected except for UDP-Rha, indicating that although Phe143, Glu371, Trp350, and Asn375 are essential for sugar-donor recognition, they do not independently determine sugar-donor specificity.

To further identify residues governing sugar-donor specificity, we conducted domain-swapping analyses between Cm1,2RhaT and CmdGlcT-1. A series of chimeric proteins was generated by replacing defined regions of Cm1,2RhaT with the corresponding segments from CmdGlcT-1. These regions were designated as regions I (residues 1–101), II (102–202), III (203–328), IV (329–372; PSPG box), and V (363–452) (Figure 4F; Supplemental Figure 11). Functional assays were conducted in Tobacco Bright Yellow 2 (BY2) suspension cells, which endogenously express enzymes capable of producing 7-*O*-glucosides. When hesperidin was used as the substrate, chimeras I, II, and IV completely lost rhamnosylation activity, whereas chimera III showed a marked reduction in activity. In contrast, chimera V retained activity comparable to that of wild-type Cm1,2RhaT. Notably, chimera II gained glucosylation activity while losing rhamnosylation activity, indicating that region II contains key residues governing sugar-donor specificity (Figure 4G). A similar pattern was observed when naringenin was used as the substrate (Supplemental Figure 12). To further refine this region, region II was subdivided into regions IIa, IIb, and IIc (Figure 4F). Functional assays showed that chimeras IIa and IIb retained weak rhamnosylation activity, whereas chimera IIc completely lost rhamnosylation activity while gaining glucosylation activity (Figures 4H and 4I). Structural modeling indicated that the sugar-donor binding pocket is primarily formed by α-helices within regions I, II, and IV, whereas region V is positioned outside the pocket (Supplemental Figure 13). Compared with Cm1, 2RhaT, the binding pocket of chimera IIc was enlarged to a size similar to that of CmdGlcT-1 (Supplemental Figures 14A–14D). This expanded pocket more readily accommodates UDP-Glc, which is bulkier than UDP-Rha due to the presence of an additional hydroxyl group (Supplemental Figure 14E). These results suggest that region IIc plays a pivotal role in determining sugar-donor specificity, likely by modulating the size and geometry of the donor-binding pocket.

Further subdivision and site-directed mutagenesis of regions I, II, and IV were subsequently performed in *E. coli* (Figure 4J).

(G) Functional analysis of chimeric proteins (chimeras I–V) using hesperetin (Hes) as the substrate in Tobacco Bright Yellow 2 (BY2) suspension cells.
(H and I) Functional analysis of chimeric proteins (chimeras IIa, IIb, and IIc) using UDP-Rha **(H)** or UDP-Glc **(I)** as the sugar donor and N7Glu as the acceptor in the *E. coli* expression system. Blue lines represent HPLC elution profiles corresponding to dGlcT activity.
(J) Relative rhamnosylation activity of chimeric and single or multiple mutant proteins expressed in *E. coli*.
H7Neo, hesperetin 7-*O*-neohesperidoside; H7Sop, hesperetin 7-*O*-sophoroside; N7Glu was used as the acceptor. N7Glu, naringenin 7-*O*-glucoside; N7Neo, naringenin 7-*O*-neohesperidoside; N7Sop, naringenin 7-*O*-sophoroside. Error bars represent standard deviation from three biological replicates. The dashed line marks a reference peak used to distinguish compounds with similar retention times.

Although none of the resulting mutants acquired glucosylation activity, the loss of rhamnosylation activity provided important insights into the functional relevance of these regions. Within region IIc, all four simultaneous substitutions in mutant chimera IIc_2 completely abolished rhamnosylation function. Among these sites, multipoint mutations involving Phe195 abolished activity, whereas mutations at the other three positions retained rhamnosylation activity, indicating that Phe195 is the most critical residue within region IIc. Additionally, replacement of the Ala–Pro–His tripeptide (residues 23–25) in region I, as well as single or multiple mutations in region IV, resulted in substantial or complete loss of rhamnosylation function (Figure 4J). Structural analyses revealed that these key residues are located at positions distal to the ligand-binding site, beyond van der Waals contact distance (Supplemental Figure 15), suggesting that their effects are mediated through indirect structural modulation rather than direct substrate interactions.

A distal residue corresponding to Phe195 modulates catalytic activity and specificity across functional citrus dGlyTs

To further assess the functional importance of Phe195 in Cm1,2RhaT, this residue was substituted with several representative amino acids. Substitutions with non-hydrophobic residues resulted in a pronounced reduction or complete loss of catalytic activity, indicating that hydrophobicity at position 195 is essential for enzyme function (Figure 5A). Among hydrophobic substitutions, F195L completely abolished catalytic activity, F195W reduced activity toward flavanone substrates while retaining high activity toward flavone substrates, and F195M significantly enhanced catalytic activity across most tested substrates (Figure 5A; Supplemental Table 5). These results indicate that, beyond hydrophobicity, the size and chemical properties of the side chain at position 195 play a critical role in regulating the rhamnosylation activity and substrate preference of Cm1,2RhaT.

Because Phe195 and its variants are located at a distal site and do not directly interact with ligands (Supplemental Figure 16), we performed 100-ns unconstrained molecular dynamics (MD) simulations of Cm1,2RhaT-F195 and Cm1,2RhaT-F195M in complex with UDP-Rha and A7Glu to explore their mechanistic roles. Based on the proposed catalytic mechanism of Cm1,2RhaT (Figure 3C), two distances were analyzed to evaluate catalytic efficiency: d1, between the NE2 atom of catalytic His21 and the O2″ atom of A7Glu, and d2, between the C1′ carbon of UDP-Rha and the O2″ atom of A7Glu. The results showed that Cm1,2RhaT-F195M had a shorter d1 and a more stable d2 than Cm1,2RhaT-F195 (Figure 5B). Representative conformations from the MD simulations showed a reduction in d1 from 4.1 Å to 2.8 Å, together with a lower Molecular Mechanics/Poisson-Boltzmann Surface Area calculated binding free energy, which may reduce the energetic barrier for deprotonation and thereby enhance catalytic activity (Figures 5C and 5D; Supplemental Figure 17F). Additionally, root-mean-square deviations of both the protein and UDP-Rha were more stable in Cm1,2RhaT-F195M, and this variant formed more hydrogen bonds between the enzyme and its ligands (Supplemental Figures 17A–17E). Collectively, these findings suggest that residue 195 modulates enzymatic activity by reshaping the binding pocket and strengthening enzyme–ligand interactions.

To assess whether this site also plays a functional role in other citrus dGlyTs, we structurally aligned multiple citrus dGlyTs with Cm1,2RhaT to identify residues corresponding to Phe195 and mutated them to phenylalanine (Phe) (Supplemental Figure 18). We then introduced substitutions at the corresponding positions in Cs1,6RhaT and UGT91BK3, which catalyze β1–6 linkages but differ in substrate promiscuity, as well as in UGT79B202 and UGT79B203, which catalyze β1–2 linkages but differ in sugar-donor specificity. Nine substrates, including flavanones, flavonols, and flavones, were used to evaluate substrate promiscuity, and four sugar donors were used to examine sugar-donor specificity.

In Cs1,6RhaT, the G216F mutation completely abolished 1,6-rhamnosylation activity, whereas the G216T mutation caused a reduction in enzyme activity (Figure 5E; Supplemental Table 5). In UGT91BK3, the T216G mutation increased activity toward the flavanone substrates N7Glu and H7Glu by 9.24- and 8.68-fold, respectively, while maintaining weak activity toward other substrates. The T216F mutation significantly enhanced substrate promiscuity and shifted the preferred substrate from flavanone to flavonol (Figure 5F; Supplemental Table 5). In UGT79B202, the N202F and N202L mutations enhanced activity toward the optimal substrate K3Glu by 1.72- and 1.73-fold, respectively (Figure 5G; Supplemental Table 5). In contrast, in UGT79B203, the L201F mutation reduced activity, whereas the L201N mutation had little effect (Figure 5H; Supplemental Table 5). Sugar-donor assays showed that mutations in UGT79B202 did not alter sugar-donor specificity or preference (Figure 5I; Supplemental Table 5). However, mutations in UGT79B203 conferred weak rhamnosylation activity in addition to glucosylation function, indicating that this site contributes to the evolution of sugar-donor specificity in UGT79B203 (Figure 5J; Supplemental Table 5).

Docking and interaction analyses showed that residues corresponding to site 195 in these citrus dGlyTs do not directly interact with substrates (Supplemental Figures 19A–19D). Structural analyses of the mutant proteins revealed that the Cs1,6RhaT-G216F substitution introduced steric hindrance between F216 and the acceptor substrate, resulting in loss of activity. No obvious steric hindrance was observed in the other mutants, suggesting that their functional effects are mediated through indirect structural mechanisms similar to those observed for Cm1,2RhaT-F195M (Supplemental Figures 19E−19H).

Potential roles of Neo accumulation in the adaptive evolution of citrus

Given its distinct evolutionary trajectory, Neo may possess biological functions that differ from those of Rut. To explore and compare the potential functional differences between these two disaccharide glycosides, we first analyzed their antioxidant activities, together with those of their corresponding aglycones and monoglucosides. The results showed that glycosylation generally reduced antioxidant capacity relative to aglycones, with no consistent trend in antioxidant reduction across different glycosylation types (Supplemental Figure 20A).

We next investigated their antifungal properties. Naringenin 7-*O*-neohesperidoside (N7Neo), the most abundant Neo compound in

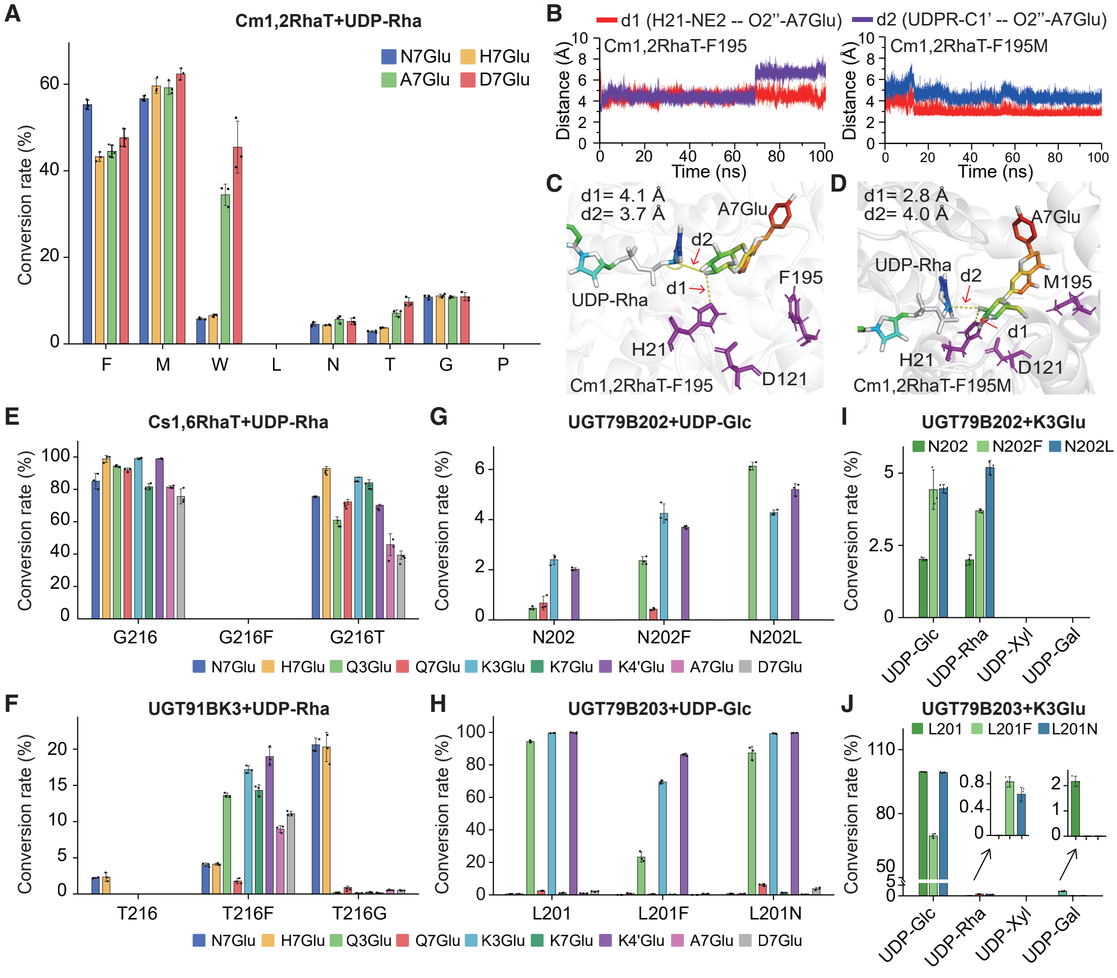

Figure 5. Functional analysis of mutations at the residue corresponding to F195 in different citrus dGlyTs.

(A) Conversion rates of Cm1,2RhaT variants carrying mutations at residue F195 using UDP-Rha as the sugar donor and nine different flavonoids as acceptors. Residue F195 was substituted with eight different amino acids (F, W, M, L, N, T, G, and P), generating eight mutant proteins.

(B) Distance variations of Cm1,2RhaT-F195 and Cm1,2RhaT-F195M during 100-ns molecular dynamics (MD) simulations. d1, distance between the nitrogen atom (NE2) of His21 and the 2-hydroxy (O2′′) of A7Glu; d2, distance between the C1′ carbon (C1′) of UDP-Rha and the 2-hydroxy (O2′′) of A7Glu.

(C and D) Representative catalytic conformations of Cm1,2RhaT-F195 **(C)** and Cm1,2RhaT-F195M **(D)** in MD simulations. Dashed lines indicate measured distances.

(E–H) Conversion rates of mutants at residues corresponding to site 195 in Cs1,6RhaT **(E)**, UGT91BK3 **(F)**, UGT79B202 **(G)**, and UGT79B203 **(H)** using nine different flavonoids as substrates and the corresponding UDP-sugar as donor.

(I and J) Conversion rates of mutants at residues corresponding to site 195 in UGT79B202 **(I)** and UGT79B203 **(J)** using K3Glu as the acceptor and four different UDP-sugars as donors.

Error bars represent standard deviations from three biological replicates. N7Glu, naringenin 7-*O*-glucoside; H7Glu, hesperetin 7-*O*-glucoside; Q3Glu, quercetin 3-*O*-glucoside; Q7Glu, quercetin 7-*O*-glucoside; K3Glu, kaempferol 3-*O*-glucoside; K7Glu, kaempferol 7-*O*-glucoside; K4′Glu, kaempferol 4′-*O*-glucoside; A7Glu, apigenin 7-*O*-glucoside; D7Glu, diosmetin 7-*O*-glucoside.

citrus, was first tested for antifungal activity against three common citrus fungal pathogens: *Colletotrichum gloeosporioides*, *Alternaria alternata*, and *Diaporthe citri*. These pathogens preferentially infect young and tender tissues, such as newly emerging shoots and young fruits. Preliminary screening revealed that N7Neo strongly inhibited the growth of *C. gloeosporioides*. We therefore compared the antifungal activities of two representative Neo compounds from pummelo with those of their corresponding Rut compounds against *Colletotrichum*. DMSO was used as a negative control, and difenoconazole, a broad-spectrum fungicide, served as a positive control. At a concentration of 400 μM, both Neo compounds exhibited significantly stronger antifungal activity than their Rut counterparts (Figure 6A; Supplemental Figure 20B). To further investigate the antifungal mechanism, we examined the ultrastructure of *Colletotrichum* hyphae treated with N7Neo or the negative control using scanning electron microscopy (SEM). Hyphae in the negative control displayed smooth and regular surfaces (Figure 6B), whereas hyphae exposed to 400 μM N7Neo appeared sunken and twisted (Figure 6C), indicating that N7Neo treatment disrupted cell wall and/or membrane integrity, thereby inhibiting fungal growth.

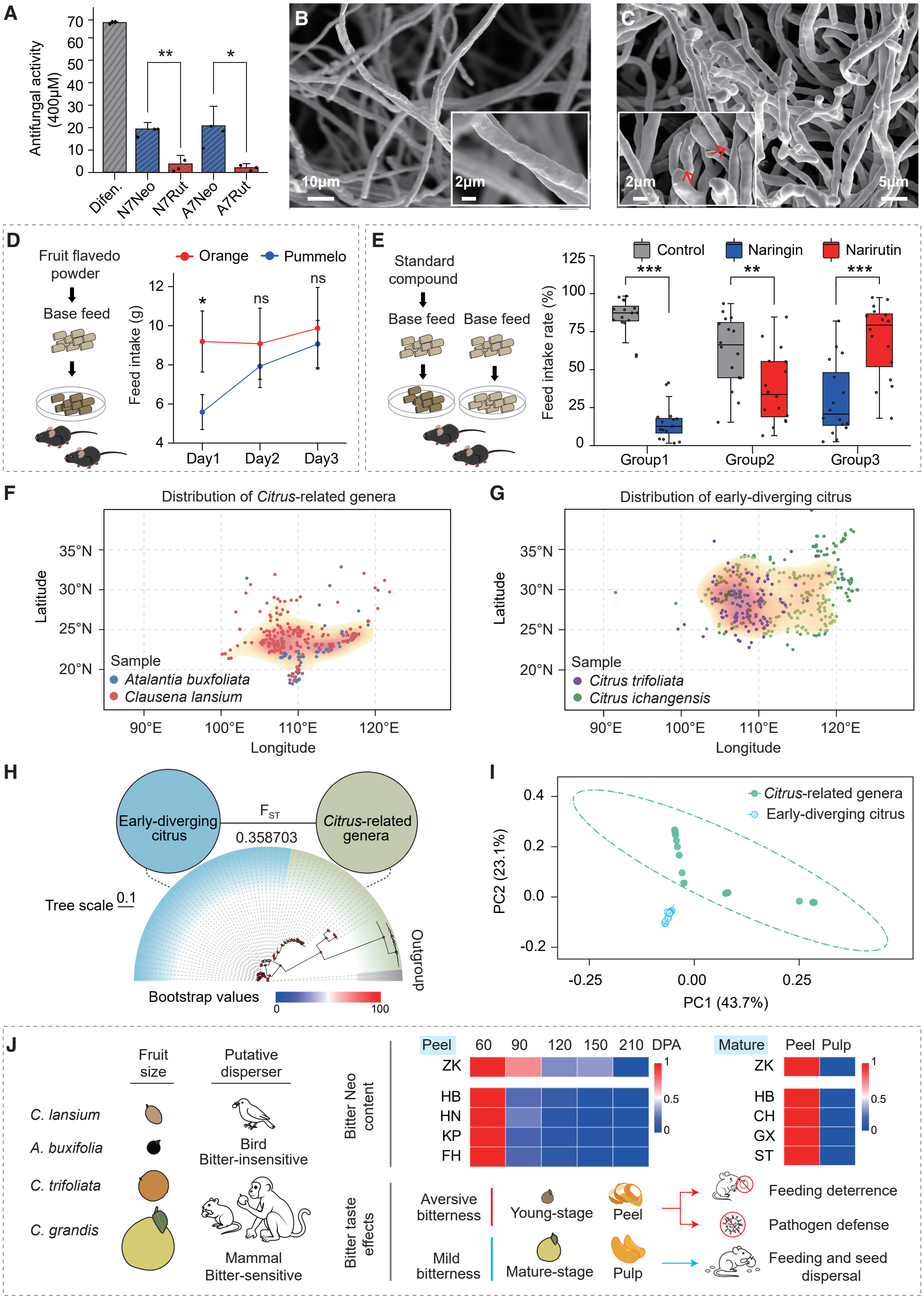

Figure 6. Biological functions of neohesperidosides and their potential roles in biotic defense and geographic distribution of *Citrus*.
(A) Antifungal activities of representative neohesperidoside and rutinoside compounds against the fungal pathogen *Colletotrichum gloeosporioides*. Difenoconazole (Difen.) was used as a positive control.
(B and C) SEM images of *Colletotrichum* hyphae exposed to the negative control (DMSO) **(B)** and N7Neo **(C)**.
(D) Left, schematic illustration of the feed intake assay. Right, feed intake of adult C57 mice over 3 days (n = 3 cages per group, 2 mice per cage).

(legend continued on next page)

Because bitter compounds are often linked to feeding deterrence, we further examined the anti-feeding effects of Neo and Rut compounds in mice. A feed-intake assay was conducted by mixing fruit flavedo powder from pummelo (Neo-rich) or sweet orange (Rut-rich) into a base diet and monitoring consumption over 3 days. On the first day, mice fed pummelo powder exhibited significantly lower feed intake than those fed orange powder (Figure 6D), indicating an immediate aversive response. However, intake in the pummelo-fed group increased on the second and third days and no longer differed significantly from that of the orange-fed group (Figure 6D), likely reflecting hunger overriding the initial aversion.

To directly compare the effects of Neo and Rut, a two-bowl feeding assay was performed using base feed as a control and base feed supplemented with either N7Neo or naringenin 7-*O*-rutinoside (N7Rut) standards (Figure 6E). In both group 1 (N7Neo vs. base feed control) and group 2 (N7Rut vs. base feed control), intake of the supplemented feed was significantly lower than that of the base feed, with N7Neo showing a lower intake rate than N7Rut. Consistently, in group 3 (N7Neo vs. N7Rut), intake of the N7Neo-supplemented feed was significantly lower than that of the N7Rut-supplemented feed. Together, these results demonstrate that Neo exhibits a stronger anti-feeding effect than Rut in mice.

To investigate whether these biological activities contribute to broader environmental adaptation, we analyzed geographic records from 341 samples of *Citrus*-related genera (*A. buxifolia* and *C. lansium*) and 439 samples of early-diverging citrus species (*C. trifoliata* and *C. ichangensis*) (Supplemental Data 6). The results showed that the distributions of *C. trifoliata* and *C. ichangensis* are significantly broader than those of *A. buxifolia* and *C. lansium* (Figures 6F and 6G). To genetically contextualize this geographic pattern, we conducted a population genomic analysis of 40 accessions from early-diverging citrus (*C. trifoliata* and *C. ichangensis*) and 30 accessions from *Citrus*-related genera (*A. buxifolia*, *Murraya paniculata*, and *C. lansium*) (Supplemental Data 7). Based on 7 158 420 high-quality SNPs, maximum likelihood (ML) phylogenetic analysis resolved two well-separated lineages corresponding to *Citrus*-related genera and early-diverging citrus. This separation is further supported by principal component analysis (PCA) clustering and high pairwise F_{ST} values (Figures 6H and 6I). Importantly, this pronounced genomic divergence mirrors contrasting metabolic phenotypes: all early-diverging citrus accessions uniformly accumulate Neo, whereas *Citrus*-related genera do not (Figure 1B). Together with the demonstrated antifungal and anti-feeding activities of Neo (Figures 6A–6E), these genomic and chemotypic differences support the conclusion that the emergence of Neo biosynthesis likely contributes to the enhanced ecological resilience of early citrus lineages and facilitates their broader geographic expansion.

DISCUSSION

The vast chemical diversity of plant metabolites is largely driven by complex modifications of core scaffolds. Recent advances, such as widely targeted metabolite modificomics, have substantially enhanced the detection and identification of modified metabolites (Yang et al., 2024). In this study, we conducted a systematic profiling of glycosylated metabolites in citrus using widely targeted metabolomic analysis. This approach refined our understanding of glycosylation diversity and revealed that the emergence of bitter Neo is closely associated with a functional shift from 2″-*O*-glucosylation to 2″-*O*-rhamnosylation.

Many studies have demonstrated that changes in one or a few amino acids, particularly at distal sites, can lead to pronounced alterations in enzyme function (Moghe and Last, 2015; Lopez-Nieves et al., 2022). For instance, only three amino acid substitutions in KAI2 conferred responsiveness to strigolactones, and a single coding SNP in *GmPM30* enhanced soybean salinity tolerance and yield (Arellano-Saab et al., 2021; Huang et al., 2025). Here, we identified a structurally conserved distal residue, Phe195 in Cm1,2RhaT and the corresponding Leu201 in UGT79B203, that modulates sugar-donor specificity and enables Neo biosynthesis. Structural comparisons across diverse plant dGlyTs revealed high variability at this position, in contrast to the conserved active-site residues essential for catalysis (Supplemental Figure 21). Unlike active-site residues, distal residues do not directly contact ligands and are therefore more prone to adaptive mutations that drive functional divergence (Soskine and Tawfik, 2010; Zong et al., 2019; Gu et al., 2023). MD simulations further showed that Phe195 reshapes the substrate-binding pocket of Cm1,2RhaT, thereby modulating enzyme activity. This mechanism is consistent with findings in UGT76G1, in which multiple distal mutations (T284S/M88L/L200A) remodel the hydrophobic pocket to accommodate the larger substrate Reb D (Guo et al., 2022). Additional studies

(E) Left, schematic illustration of the feed intake rate assay. Right, feed intake of adult C57 mice recorded every 24 h for different treatment groups (n = 8 cages per group, 2 mice per cage). Group 1 received base feed and feed supplemented with naringin. Group 2 received base feed and feed supplemented with narirutin. Group 3 received feed supplemented with naringin and feed supplemented with narirutin. The final supplement concentration was 30 mg/g. N7Neo, naringenin 7-*O*-neohesperidoside; N7Rut, naringenin 7-*O*-rutinoside; A7Neo, apigenin 7-*O*-neohesperidoside; A7Rut, apigenin 7-*O*-rutinoside. Data are presented as mean ± SD. Statistical significance was determined using Student's t-test. ns, not significant; $*p < 0.05$, $**p < 0.01$, $***p < 0.001$.
(F and G) Geographic distribution of *Citrus*-related genera (*Clausena lansium* and *A. buxifolia*) **(F)** and early-diverging citrus (*C. trifoliata* and *C. ichangensis*) **(G)**.
(H) Phylogenetic and population divergence analysis. A maximum likelihood (ML) phylogeny was constructed with RAxML using 1000 bootstrap replicates. Two outgroup accessions were included to root the tree. Population divergence (F_{ST}) among geographic groups was calculated in sliding windows of 50 kb with a 10 kb step size.
(I) PCA analysis based on genome-wide SNPs.
(J) Schematic model illustrating the effect of citrus bitter compounds on feeding behavior. Small-fruited species such as *C. lansium* and *A. buxifolia* are likely adapted for bird-mediated seed dispersal, which is insensitive to bitterness, whereas larger-fruited species such as *C. trifoliata* and *C. grandis* are adapted for mammalian dispersers that are sensitive to bitterness. Bitter neohesperidosides accumulate at high levels in young fruits and peels, potentially functioning in both feeding deterrence and pathogen defense, while reduced accumulation at maturity may facilitate fruit consumption and seed dispersal.

have shown that distal residues can influence enzyme function indirectly through residue–residue interaction networks or by altering the geometry and accessibility of the substrate channel (Tan et al., 2022). Together, these findings provide a molecular and structural basis for understanding how amino acid variation drives enzyme functional evolution.

Gene duplication is a major driver of metabolic innovation. Although most duplicated genes are eventually lost, some are selectively retained following acquisition of beneficial new functions through neofunctionalization (Huang et al., 2018; Peng et al., 2024). Our findings indicate that the Neo biosynthetic genes (*Cm1,2RhaT* and *UGT79B202*) originated from duplication of ancestral *dGlcT* genes and were retained because of the strong antifungal and anti-feeding activities conferred by Neo (Figures 6A–6E). Based on these findings, we propose a speculative model of a bitterness-mediated defense strategy in citrus (Figure 6J). *Citrus*-related species such as *C. lansium* and *A. buxifolia*, produce relatively small fruits, for which seed dispersal may rely on bitter-insensitive birds. In contrast, species such as *C. trifoliata* and *C. grandis* produce larger fruits that are more likely dispersed by bitter-sensitive mammals. Neo accumulation was observed in *C. trifoliata* (ZK) and in four *C. grandis* accessions (HB, HN, KP, and FH) across multiple developmental stages and tissues. Neo levels were highest in young fruits and gradually declined during fruit maturation, with mature fruits showing significantly higher Neo accumulation in the peel than in pulp tissues. This spatiotemporal pattern suggests a dual function for Neo: providing feeding deterrence and pathogen defense during early fruit development, while decreasing in edible tissues (pulp) at ripening to facilitate fruit consumption and seed dispersal. Such dynamic regulation aligns with general models of the functions of secondary metabolites in fruit, which balance defense and reproduction (Herrera, 1982; Tewksbury et al., 2008). For instance, capsaicinoid levels decline during fruit ripening in chili (*Capsicum chacoense*) (Tewksbury et al., 2008), and glucosinolates show tissue- and stage-specific accumulation patterns in *Arabidopsis thaliana* (Brown et al., 2003). Importantly, we also observed that two major limonoids in citrus, limonin and nomilin, which are responsible for delayed bitterness, accumulate predominantly in seeds, highlighting a potential division of labor between limonoids and Neo in citrus defense (De La Pena et al., 2023). In other tissues, limonoids show spatial distribution patterns similar to those of Neo, with high abundance in the peel and segment membranes (Supplemental Data 8), further supporting the proposed defense model. Together, these findings illustrate how the spatial and temporal regulation of bitterness contributes to adaptive trade-offs between defense and seed dispersal during citrus evolution.

The emergence of bitter Neo likely plays a significant role in the dissemination and ecological expansion of *Citrus*. Our findings show that the non-bitter Rut is present in both citrus and *Citrus*-related species, whereas Neo emerges specifically in early-diverging citrus lineages. *Citrus*-related genera such as *C. lansium* and *A. buxifolia* are distributed in tropical and subtropical regions (Figure 6F), where herbivory pressure is primarily insect-driven (Becerra, 2015). In contrast, early-diverging citrus species, including *C. trifoliata* and *C. grandis*, inhabit subtropical to temperate regions (Figure 6G), where bitter-sensitive mammals impose stronger selective pressure (Becerra, 2015). Notably, diversification of early-diverging citrus occurred during the late Miocene epoch, a period characterized by extensive mammalian radiation (Bininda-Emonds et al., 2007). Together, these observations suggest that the emergence of Neo represents an adaptive metabolic innovation in response to shifting herbivore pressure during this ecological transition. While Wu et al. (2018) emphasized climatic factors as primary drivers of citrus expansion, our results highlight the importance of biotic interactions, including pathogens and herbivores, as additional selective forces shaping citrus evolution.

Beyond natural selection, domestication has profoundly influenced the distribution and abundance of Neo in citrus. Previous studies demonstrated that artificial selection against bitterness led to the loss of the *1,2RhaT* gene in modern sweet orange cultivars (Chen et al., 2019). In this study, we further confirmed that multiple single-amino-acid substitutions in Cm1,2RhaT result in loss of enzyme function (Figure 5). Such mutations are present in modern commercial pummelo cultivars, including Guanximi and Shatian, which exhibit significantly lower Neo content (Supplemental Figure 22). In contrast, Zhengmao and Jiaxiyang pummelos—traditional cultivars from Huazhou in Guangdong Province used in medicinal preparations—retain a functional *1,2RhaT* gene and accumulate high levels of Neo, suggesting that Neo may have been selectively preserved for its medicinal value. A comparable pattern has been reported in cucumber, where selection on the Bt transcription factor during domestication led to the loss of bitterness (Shang et al., 2014). As cultivated plants experience reduced environmental pressures, defense strategies and metabolite profiles are often reshaped accordingly (Zust and Agrawal, 2017). The evolutionary gain and subsequent loss of Neo biosynthesis in citrus exemplify the complex interplay between natural and artificial selection in shaping plant specialized metabolism and phenotypic diversity.

In conclusion, our study demonstrates that two pairs of paralogous *dGlyTs* contribute to Neo biosynthesis in citrus. In early-diverging citrus species, gene duplication followed by neofunctionalization drove the evolution of rhamnosyltransferase activity required for Neo production. We identify a critical amino acid residue, corresponding to Phe195 in Cm1,2RhaT and Leu201 in UGT79B203, as a key determinant of this neofunctionalization and demonstrate that Neo possesses stronger antifungal and anti-herbivory activities than Rut. These traits likely facilitated the ecological expansion of early-diverging citrus beyond their *Citrus*-related ancestors. Overall, this study provides new insights into the evolutionary trajectory of bitter Neo in *Citrus* and underscores the adaptive roles of specialized metabolites in plant–environment interactions, with potential implications for citrus flavor improvement and functional food development.

METHODS

Plant materials

Fruits from 38 citrus accessions were used in this study, covering *Citrus*-related genera (*C. lansium* and *A. buxifolia*), early-diverging citrus (*C. trifoliata*, *C. ichangensis*, and *C. mangshanensis*), and domesticated citrus (*C. grandis*, *C. reticulata*, *C. medica*, *C. aurantium*, *C. sinensis*, and *C. limon*) (Supplemental Table 1). For each accession, 15–18 fruits

were randomly harvested from at least three healthy trees at the commercial harvest stage, approximately 230 days post anthesis. Fruits were randomly divided into three biological replicates. Flavedo tissues were isolated, immediately frozen in liquid nitrogen, and stored at −80°C until analysis. Collection locations and detailed accession information are provided in Supplemental Table 1.

Commercially available chemical standards

Chemical standards were purchased from Shanghai Yuanye Bio-Technology (Shanghai, China). These included flavanones (naringenin 7-*O*-glucoside, hesperetin 7-*O*-glucoside), flavonols (quercetin 3-*O*-glucoside, quercetin 7-*O*-glucoside, kaempferol 3-*O*-glucoside, kaempferol 7-*O*-glucoside, kaempferol 4′-*O*-glucoside), flavones (apigenin 7-*O*-glucoside, diosmetin 7-*O*-glucoside), three sugar donors (UDP-glucose, UDP-xylose, and UDP-galactose), and flavonoid disaccharide glycosides (naringenin 7-*O*-neohesperidoside, naringenin 7-*O*-rutinoside, hesperetin 7-*O*-neohesperidoside, hesperetin 7-*O*-rutinoside, quercetin 3-*O*-neohesperidoside, quercetin 3-*O*-sophoroside, kaempferol 3-*O*-neohesperidoside, and kaempferol 3-*O*-sophoroside). UDP-rhamnose was purchased from Angfei Biological Technology (Guangdong, China). Acyclovir and roxithromycin were obtained from Sigma-Aldrich (USA). Detailed information on all chemicalstandards used in this study is provided in Supplemental Table 6.

Metabolic profiling

Sample preparation for metabolic analysis was modified from a previously described method (Peng et al., 2024). All samples were freeze-dried using a lyophilizer (Heto Lylab 3000) and ground into a fine powder. Powdered samples (50 ± 5 mg) were extracted with 0.5 ml of 70% methanol containing 0.4% internal standards (acyclovir and roxithromycin, 0.1 ppm each). After ultrasonic treatment at 4°C for 20 min, samples were centrifuged at 12 000 rpm for 30 min at 4°C. The supernatant was filtered through a 0.22-μm membrane and analyzed by high-performance liquid chromatography (HPLC) and liquid chromatography–mass spectrometry (Ultimate 3000 Nano and Thermo Q Exactive Plus Orbitrap, Thermo Scientific, Waltham, MA, USA) as described by Chen et al. (2013, 2020b). Metabolites were identified by comparison with commercial standards, in-house metabolite databases (Chen et al., 2013, 2020b), and the public mzCloud database (https://www.mzcloud.org/). Relative quantification was performed using scheduled multiple reaction monitoring on a triple quadrupole–linear ion trap mass spectrometer (QTRAP 6500$^+$, AB SCIEX, Framingham, MA, USA). Data acquisition and processing were conducted using Analyst 1.5 software. Each sample was analyzed using three biological replicates.

Gene identification and phylogenetic analysis

Citrus UGTs were identified using the Simple HMM Search program in TBtools based on the UGT hidden Markov model profile PF00201 obtained from the Pfam database (http://pfam.xfam.org/) (Chen et al., 2020a). Predicted *UGT*s containing ORFs encoding 340–680 amino acids were selected for phylogenetic analysis following the criteria of Wilson and Tian (2019). Protein sequences of citrus UGTs and 37 previously characterized UGTs representing different phylogenetic groups were aligned using multiple sequence comparison by log-expectation in MEGA X. Phylogenies were inferred by neighbor joining with 3000 bootstrap replicates. Information on the known UGTs used for phylogenetic analysis is provided in Supplemental Table 2. Similarly, for phylogenetic analysis of plant dGlyTs, functional citrus dGlyTs and 17 characterized dGlyTs from other plant species were aligned using multiple sequence comparison by log-expectation, and phylogenies were inferred by neighbor joining with 3000 bootstrap replicates. Information on the dGlyTs included in this analysis is provided in Supplemental Table 3.

Protein expression and purification

Recombinant proteins corresponding to candidate *UGTs* and their mutant variants were produced in *E. coli*. Protein expression and purification followed previously described procedures (Li et al., 2022). Briefly, the relevant coding sequences were fused to a maltose-binding protein tag in the pMAL-c2x expression vector and subsequently transformed into *E. coli* BL21(DE3) cells. Protein expression was induced with isopropyl β-D-1-thiogalactopyranoside (IPTG) to a final concentration of 0.5 mM at 16°C for 16 h. Cells were harvested and lysed by sonication at 4°C in phosphate-buffered saline (pH 7.4) containing 400 mM NaCl. Target proteins were purified using a dextrin beads 6FF affinity column with a wash buffer (20 mM Tris–HCl, 20 mM NaCl, 1 mM EDTA, and 1 mM DTT, pH 7.4), followed by elution buffer (20 mM Tris–HCl, 1 mM EDTA, 10 mM maltose, and 1 mM DTT, pH 7.4).

Enzyme activity assay and kinetics

Enzyme activity assays were performed in a total reaction volume of 100 μl containing 15 μg of purified protein, 0.1 mM flavonoid substrate, 0.5 mM UDP-sugar, and 0.25 mM $MgCl_2$ in phosphate-buffered saline (50 mM, pH 7.4). Reactions were incubated for 0.5 h at 35 °C and terminated by adding 100 μl of ice-cold methanol prior to further analysis.

For crude enzyme extraction used in saturation mutagenesis experiments, bacterial cultures were established by inoculating 1 ml of Luria–Bertani liquid medium with 10 μl of overnight-activated cells. Cultures were incubated in a 24-well plate for 2.5 h at 37°C and then induced with IPTG to a final concentration of 0.5 mM at 16°C for 16 h. Cells were harvested and lysed in phosphate-buffered saline (pH 7.4) containing 750 mg/l lysozyme and 10 mg/l benzonase for 1 h at 37°C. Crude protein extracts were obtained from the supernatant following centrifugation. Crude enzyme assays were performed in a total volume of 100 μl containing 50 μl of supernatant, 0.1 mM flavonoid substrates, 0.5 mM UDP-sugar, and 0.25 mM $MgCl_2$ in phosphate-buffered saline (50 mM, pH 7.4) for 10 h at 35°C.

To determine the kinetic parameters of UGT79B202 toward UDP-Glc and UDP-Rha, enzyme assays were carried out using kaempferol 3-*O*-glucoside (0.1 mM) and varying concentrations of UDP-Glc or UDP-Rha (0.01–0.9 mM). Reactions contained 50 ng of purified recombinant UGT79B202 in a total volume of 100 μl and were incubated at 40°C for 120 min. Kinetic parameters were calculated using the Michaelis–Menten model (OriginPro 2021).

Genome evolution analysis

MCscan (Python version) (Wang et al., 2012) was used to identify synteny among the genomes of *Vitis vinifera*, *C. lansium*, *L. scandens*, *A. buxifolia*, *C. trifoliata*, *C. mangshanensis*, *C. ichangensis*, *C. medica*, *C. limon*, *C. grandis*, *C. aurantium*, *C. sinensis*, and *C. clementina*. Reference genome sequences of citrus and citrus-related genera were downloaded from the Citrus Pan-genome to Breeding Database (http://citrus.hzau.edu.cn/index.php).

Generation of mutant gene sequences

DNA sequences corresponding to chimera I–V, Ia–c, IIa–c, IIc_1, and IIc_2 were synthesized by Beijing Tsingke Biotech. Single-point and multipoint mutations were generated using the Mut Express II Fast Mutagenesis Kit V2 (https://www.vazyme.com/product/79.html). All generated sequences were verified by DNA sequencing at Beijing Tsingke Biotech. Primers used for mutagenesis experiments are listed in Supplemental Data 9.

Identification and quantification of flavonoids

Mass spectrometry data were acquired using a 1200 Series Rapid Resolution Ultra Performance Liquid Chromatography system coupled to a 1260 Infinity diode array detector and a 6520 Accurate-Mass Quadrupole Time-of-Flight (Q-TOF) MS system (Agilent Technologies, CA, USA). Ultrapure water and acetonitrile containing 0.04% (v/v) formic acid were used as mobile phases A and B, respectively. Flavonoid compounds were identified by comparing retention times, UV spectra, mass spectra, and ion fragmentation patterns with those of commercial chemical standards. Compounds lacking authentic standards were predicted based on molecular weight and characteristic ion fragments.

Flavonoid compounds were quantified using an HPLC system with the following conditions: chromatographic column (Accucore C18, 150 × 2.1 mm; Thermo Scientific, Waltham, MA, USA); mobile phase (A: ultrapure water containing 0.15% [v/v] formic acid; B: acetonitrile containing 0.15% [v/v] formic acid); gradient elution system (0 min, 10% B;10 min, 22% B; 20 min, 23% B; 25 min, 75% B; 27 min, 75% B; 30 min, 10% B; flow rate: 0.35 ml/min). Flavonoid substrates and their glycosides were quantified according to Yuan et al. (2024). Conversion rates were calculated as percentages based on peak areas of glycosylated products and corresponding sugar acceptors in HPLC chromatograms. Each reaction was performed with three independent experimental replicates.

Biotransformation assay in tobacco bright yellow 2 (BY2) cells

Transgenic BY2 cells expressing Cm1,2RhaT, CmdGlcT-1, or chimeras I–V were generated as previously described (Chen et al. 2019). Target genes were cloned into the Ph7WG2D vector harboring GFP and introduced into *Agrobacterium tumefaciens* GV3101. Positive transformants were confirmed by fluorescence detection. For biotransformation assays, substrate-feeding experiments were performed according to the protocol described by Frydman et al. (2013). Briefly, BY2 cells were cultured in Erlenmeyer flasks for 1 week at 28°C with shaking at 140 rpm. Then, 1 g of calli was transferred to 20 ml of Murashige and Skoog liquid medium and incubated under the same conditions for 4 days. Substrates were then added, and cultures were incubated for an additional 2 days prior to cell harvest and flavonoid extraction.

Molecular docking

Protein structure models were generated using AlphaFold3 (https://golgi.sandbox.google.com/). 3D structures of UDP-Rha and UDP-Glc, naringenin 7-*O*-glucoside, hesperetin 7-*O*-glucoside, apigenin 7-*O*-glucoside, and kaempferol 3-*O*-glucoside were extracted from the PubChem compound database (https://pubchem.ncbi.nlm.nih.gov/). Molecular structures of the substrates were optimized using the B97-3c composite method implemented in ORCA (v5.0.3) (Neese, 2022). Docking calculations were performed using AutoDock Vina to obtain enzyme–substrate complexes (Trott and Olson, 2010). Structural analyses and visualization were conducted using PyMOL for educational use (v2.4.1).

Molecular dynamics (MD) simulations

MD simulations were conducted using GROMACS 2020.6. The Amber14SB force field was applied for protein simulations. Parameters for UDP-Rha and A7Glu (apigenin 7-*O*-glucoside) were generated using Sobtop_1.0 (dev3) (http://sobereva.com/soft/Sobtop) and Multiwfn (Lu and Chen, 2011; Lu, 2024) applying the GAFF force field. To derive RESP2 charges for parameterization, single-point energy calculations were performed at the B3LYP-D3/def2-TZVP level of theory based on wavefunctions obtained from structure optimization using ORCA. The system was placed in a cubic simulation box with a distance of 10 Å between the protein surface and the box boundary. Solvation was carried out using the TIP3P water model under periodic boundary conditions, and sodium ions were added to neutralize the system. Energy minimization consisted of 2500 steps of the steepest descent algorithm followed by 2500 steps of the conjugate gradient method, with a total force threshold of 100 kJ $mol^{-1}nm^{-1}$ to reduce atomic collisions and ensure that the system reached the minimum energy state. The system was pre-equilibrated under NPT conditions at 1.0 bar and a temperature of 298.15 K. A 100-ns MD simulation employing a 2-fs integration time step was subsequently conducted. Visual MD and QtGrace were used to analyze the results.

Antioxidant assay

Total antioxidant capacity was determined using the 2,2′-azinobis(3-ethylbenzothiazoline-6-sulfonic acid) (ABTS) and 2,2-diphenyl-1-picrylhydrazyl (DPPH) radical scavenging assay, as well as the ferric ion reducing antioxidant power (FRAP) assay. Both ABTS and FRAP kits were purchased from Nanjing Jiancheng Bioengineering Institute, while the DPPH kit was purchased from Shanghailianzu Biotechnology.All assays were performed according to the manufacturer's instructions.

Antifungal assay and ultrastructure analysis

Strains of *C. gloeosporioides*, *A. alternata*, and *D. citri* were kindly provided by Professor Hongye Li from Zhejiang University. Fungal strains were activated on potato dextrose agar medium at 28°C. For antifungal assays, mycelial plugs excised from the margins of 3-day-old colonies were transferred to potato dextrose agar plates supplemented with 400 μM of the tested antifungal compounds (N7Neo, N7Rut, A7Neo, A7Rut). Potato dextrose agar plates containing an equal concentration of difenoconazole served as positive controls, while plates with an equal volume of DMSO were used as negative controls. After incubation for 72 h at 28°C, colony diameters were measured. Each experiment was performed with three biological replicates.

For SEM analyses, hyphal samples treated with N7Neo (400 μM) or DMSO (control) were collected and fixed overnight in 2.5% glutaraldehyde at 4°C. Samples were then examined using a scanning electron microscope (Hitachi SU-8010, Tokyo, Japan).

Anti-feeding assay

A feed intake assay was conducted using 12 male C57 mice. Following a pre-experiment to record baseline daily food intake, mice were housed in six cages and divided into two groups based on intake. Base feed was ground and mixed with pummelo or orange albedo powder at a final concentration of 15 mg/g. The mixtures were reshaped into pellets and air-dried for 24 h. Food intake for each cage was recorded every 24 h for 3 days.

A two-bowl feeding preference assay was conducted using 16 male C57 mice. After a pre-experiment to record baseline intake, mice were housed in eight cages. Base feed was ground and mixed with standard compounds to a final concentration of 30 mg/g, reshaped into pellets, and air-dried for 24 h. Control feed was prepared in the same manner without additives. Three treatment groups were established: group 1 received control base feed and N7Neo-supplemented feed, group 2 received control base feed and N7Rut-supplemented feed, and group 3 received N7Neo- and N7Rut-supplemented feeds. Feed intake was recorded every 24 h for 2 days, and intake rates were calculated to assess feeding preference.

All animal procedures were approved by the Institutional Animal Care and Use Committee of Huazhong Agricultural University (HZAUMO-2025-0215).

Geographical distribution

Distribution records for 341 samples of *Citrus*-related genera species (*A. buxifolia* and *C. lansium*) and 439 samples of early-diverging citrus (*C. trifoliata* and *C. ichangensis*) were collected from the Chinese Virtual Herbarium (https://www.cvh.ac.cn/) and the National Specimen Information Infrastructure (http://www.nsii.org.cn/node/79/cvh/12/f95/4850757). Coordinates and associated metadata are provided in Supplemental Data 2. Geographic distribution maps were generated using Python. Kernel density estimation was applied to records within the geographic range of 15°–40°N latitude and 85° − 130°E longitude. To emphasize areas of high specimen density, only the upper 50th percentile of kernel density values was visualized.

SNP calling and population genetic analysis

A total of 40 accessions of early-diverging citrus (*C. trifoliata* and *C. ichangensis*) and 30 accessions of *Citrus*-related genera (*A. buxifolia*, *M. paniculata*, and *C. lansium*), together with two outgroup accessions, were used for population genomic analyses (Supplemental Data 6). Following read mapping, variant calling, quality filtering, and linkage disequilibrium pruning, a high-confidence dataset of 7 158 420 SNPs

was obtained for downstream analyses. ML phylogenetic inference was performed using RAxML (v7.7.8) software (Stamatakis, 2006) with 1000 bootstrap replicates. PCA was conducted using PLINK2 (Purcell et al., 2007), and pairwise genetic differentiation (F_{ST}) was calculated using VCFtools v.0.1.16 (Danecek et al., 2011) with sliding windows of 50 kb and a 10-kb step size.

Statistical analysis

All experiments were performed with at least three biological replicates. Graphs were generated using Origin 2021 Learning Edition (Microcal Software, Northampton, MA, USA). Statistical significance was assessed using one-way ANOVA followed by Tukey's multiple range test or by Student's *t*-test in SPSS (SPSS, Chicago, IL). Structural formulas were drawn using ChemBioDraw Ultra 12.0 (PerkinElmer Informatics, Waltham, MA, USA).

FUNDING

This work was supported by the National Key Research and Development Program of China (grant no. 2023YFD2300600 to J.X. and J.C.), the National Natural Science Foundation of China (grant no. 32402481 to Z.Y.), and the National Natural Science Foundation of China (grant no. 32272685 to J.C.).

ACKNOWLEDGMENTS

The authors would like to thank Dongqin Li (Huazhong Agricultural University, China) for assistance with QTRAP mass spectrometry analyses. Patent applications related to the genes *UGT79B202*, *UGT79B203*, and *UGT91BK3* are currently in preparation.

AUTHOR CONTRIBUTIONS

J.X. and J.C. conceived the project and overall strategy. G.L. designed the experiments and analyzed the data. G.L., H. Zhou, and Y.L. performed metabolite data analysis. H.W. assisted with bioinformatics analyses. G.L. conducted molecular docking and MD analyses. H.Zhang., Z.Y., Z.L., Z.H., Q.C., and G.C. helped with biochemical experiments. G. L. wrote the manuscript with contributions from J.-L.Y., J.X., and J.C. All authors discussed the results and approved the final version of the manuscript.

SUPPLEMENTAL INFORMATION

Supplemental information is available at *Plant Communications Online*.

Received: August 12, 2025
Revised: October 6, 2025
Accepted: January 4, 2026
Published: January 5, 2026

REFERENCES

Arellano-Saab, A., Bunsick, M., Al Galib, H., Zhao, W., Schuetz, S., Bradley, J.M., Xu, Z., Adityani, C., Subha, A., McKay, H., et al. (2021). Three mutations repurpose a plant karrikin receptor to a strigolactone receptor. Proc. Natl. Acad. Sci. USA **118**:e2103175118. https://doi.org/10.1073/pnas.2103175118.

Becerra, J.X. (2015). On the factors that promote the diversity of herbivorous insects and plants in tropical forests. Proc. Natl. Acad. Sci. USA **112**:6098–6103. https://doi.org/10.1073/pnas.1418643112.

Bininda-Emonds, O.R.P., Cardillo, M., Jones, K.E., MacPhee, R.D.E., Beck, R.M.D., Grenyer, R., Price, S.A., Vos, R.A., Gittleman, J.L., and Purvis, A. (2007). The delayed rise of present-day mammals. Nature **446**:507–512. https://doi.org/10.1038/nature05634.

Brown, P.D., Tokuhisa, J.G., Reichelt, M., and Gershenzon, J. (2003). Variation of glucosinolate accumulation among different organs and developmental stages of *Arabidopsis thaliana*. Phytochemistry **62**:471–481. https://doi.org/10.1016/s0031-9422(02)00549-6.

Chen, C., Chen, H., Zhang, Y., Thomas, H.R., Frank, M.H., He, Y., and Xia, R. (2020a). TBtools: An integrative toolkit developed for interactive analyses of big biological data. Mol. Plant **13**:1194–1202. https://doi.org/10.1016/j.molp.2020.06.009.

Chen, H.Y., and Li, X. (2017). Identification of a residue responsible for UDP-sugar donor selectivity of a dihydroxybenzoic acid glycosyltransferase from *Arabidopsis* natural accessions. Plant J. **89**:195–203. https://doi.org/10.1111/tpj.13271.

Chen, J., Hu, X., Shi, T., Yin, H., Sun, D., Hao, Y., Xia, X., Luo, J., Fernie, A.R., He, Z., and Chen, W. (2020b). Metabolite-based genome-wide association study enables dissection of the flavonoid decoration pathway of wheat kernels. Plant Biotechnol. J. **18**:1722–1735. https://doi.org/10.1111/pbi.13335.

Chen, J., Yuan, Z., Zhang, H., Li, W., Shi, M., Peng, Z., Li, M., Tian, J., Deng, X., Cheng, Y., et al. (2019). Cit1,2RhaT and two novel CitdGlcTs participate in flavor-related flavonoid metabolism during citrus fruit development. J. Exp. Bot. **70**:2759–2771. https://doi.org/10.1093/jxb/erz081.

Chen, W., Gong, L., Guo, Z., Wang, W., Zhang, H., Liu, X., Yu, S., Xiong, L., and Luo, J. (2013). A novel integrated method for large-scale detection, identification, and quantification of widely targeted metabolites: application in the study of rice metabolomics. Mol. Plant **6**:1769–1780. https://doi.org/10.1093/mp/sst080.

Chen, Y., Yan, Q., Ji, Y., Bai, X., Li, D., Mu, R., Guo, K., Yang, M., Tao, Y., Gershenzon, J., et al. (2023). Unraveling the serial glycosylation in the biosynthesis of steroidal saponins in the medicinal plant *Paris polyphylla* and their antifungal action. Acta Pharm. Sin. B **13**:4638–4654. https://doi.org/10.1016/j.apsb.2023.05.033.

Cho, M.H., and Lee, S.W. (2015). Phenolic phytoalexins in rice: biological functions and biosynthesis. Int. J. Mol. Sci. **16**:29120–29133. https://doi.org/10.3390/ijms161226152.

Danecek, P., Auton, A., Abecasis, G., Albers, C.A., Banks, E., DePristo, M.A., Handsaker, R.E., Lunter, G., Marth, G.T., Sherry, S.T., et al. (2011). The variant call format and VCFtools. Bioinformatics **27**:2156–2158. https://doi.org/10.1093/bioinformatics/btr330.

De La Pena, R., Hodgson, H., Liu, J.C., Stephenson, M.J., Martin, A.C., Owen, C., Harkess, A., Leebens-Mack, J., Jimenez, L.E., Osbourn, A., et al. (2023). Complex scaffold remodeling in plant triterpene biosynthesis. Science **379**:361–368. https://doi.org/10.1126/science.adf1017.

Forster, C., Handrick, V., Ding, Y., Nakamura, Y., Paetz, C., Schneider, B., Castro-Falcon, G., Hughes, C.C., Luck, K., Poosapati, S., et al. (2022). Biosynthesis and antifungal activity of fungus-induced *O*-methylated flavonoids in maize. Plant Physiol. **188**:167–190. https://doi.org/10.1093/plphys/kiab496.

Frydman, A., Liberman, R., Huhman, D.V., Carmeli-Weissberg, M., Sapir-Mir, M., Ophir, R., W Sumner, L., and Eyal, Y. (2013). The molecular and enzymatic basis of bitter/non-bitter flavor of citrus fruit: evolution of branch-forming rhamnosyltransferases under domestication. Plant J. **73**:166–178. https://doi.org/10.1111/tpj.12030.

Frydman, A., Weisshaus, O., Bar-Peled, M., Huhman, D.V., Sumner, L.W., Marin, F.R., Lewinsohn, E., Fluhr, R., Gressel, J., and Eyal, Y. (2004). Citrus fruit bitter flavors: isolation and functional characterization of the gene *Cm1,2RhaT* encoding a 1,2 rhamnosyltransferase, a key enzyme in the biosynthesis of the bitter flavonoids of citrus. Plant J. **40**:88–100. https://doi.org/10.1111/j.1365-313X.2004.02193.x.

Gouot, J.C., Smith, J.P., Holzapfel, B.P., Walker, A.R., and Barril, C. (2019). Grape berry flavonoids: a review of their biochemical responses to high and extreme high temperatures. J. Exp. Bot. **70**:397–423. https://doi.org/10.1093/jxb/ery392.

Gu, J., Xu, Y., and Nie, Y. (2023). Role of distal sites in enzyme engineering. Biotechnol. Adv. **63**:108094. https://doi.org/10.1016/j.biotechadv.2023.108094.

Guo, B., Deng, Z., Meng, F., Wang, Q., Zhang, Y., Yuan, Z., and Rao, Y. (2022). Enhancement of Rebaudioside M production by structure-guided engineering of glycosyltransferase UGT76G1. J. Agric. Food Chem. **70**:5088–5094. https://doi.org/10.1021/acs.jafc.2c01209.

Herrera, C.M. (1982). Defense of ripe gruit from pests: its significance in relation to plant-disperser interactions. Am. Nat. **120**:218–241. https://doi.org/10.1086/283984.

Huang, D., Wang, X., Tang, Z., Yuan, Y., Xu, Y., He, J., Jiang, X., Peng, S.-A., Li, L., Butelli, E., et al. (2018). Subfunctionalization of the *Ruby2–Ruby1* gene cluster during the domestication of citrus. Nat. Plants **4**:930–941. https://doi.org/10.1038/s41477-018-0287-6.

Huang, S., Xia, Y., Yang, J., Si, Y., Chen, X., Zhang, H., Liu, T., Zheng, W., Chen, X., Zhao, Z., et al. (2025). A Coding SNP in *GmPM30* enhances Soybean salinity tolerance and yield through the GmLEA1-GmPM30-GmLEC1 module. Adv. Sci. (Weinh.) **12**:e09391. https://doi.org/10.1002/advs.202509391.

Huang, Y., He, J., Xu, Y., Zheng, W., Wang, S., Chen, P., Zeng, B., Yang, S., Jiang, X., Liu, Z., et al. (2023). Pangenome analysis provides insight into the evolution of the orange subfamily and a key gene for citric acid accumulation in citrus fruits. Nat. Genet. **55**:1964–1975. https://doi.org/10.1038/s41588-023-01516-6.

Li, W., Li, G., Yuan, Z., Li, M., Deng, X., Tan, M., Ma, Y., Chen, J., and Xu, J. (2022). Illustration of the variation in the content of flavanone rutinosides in various citrus germplasms from genetic and enzymatic perspectives. Hortic. Res. **9**:uhab017. https://doi.org/10.1093/hr/uhab017.

Lopez-Nieves, S., El-Azaz, J., Men, Y., Holland, C.K., Feng, T., Brockington, S.F., Jez, J.M., and Maeda, H.A. (2022). Two independently evolved natural mutations additively deregulate TyrA enzymes and boost tyrosine production in planta. Plant J. **109**:844–855. https://doi.org/10.1111/tpj.15597.

Lu, T. (2024). A comprehensive electron wavefunction analysis toolbox for chemists, Multiwfn. J. Chem. Phys. **161**:082503. https://doi.org/10.1063/5.0216272.

Lu, T., and Chen, F. (2012). Multiwfn: A multifunctional wavefunction analyzer. J. Comput. Chem. **33**:580–592. https://doi.org/10.1002/jcc.22885.

Moghe, G.D., and Last, R.L. (2015). Something old, something new: conserved enzymes and the evolution of novelty in plant specialized metabolism. Plant Physiol. **169**:1512–1523. https://doi.org/10.1104/pp.15.00994.

Neese, F. (2022). Software update: The ORCA program system—Version 5.0. WIREs Comput. Mol. Sci. **12**:e1606. https://doi.org/10.1002/wcms.1606.

Noda-Garcia, L., Liebermeister, W., and Tawfik, D.S. (2018). Metabolite-enzyme coevolution: from single enzymes to metabolic pathways and networks. Annu. Rev. Biochem. **87**:187–216. https://doi.org/10.1146/annurev-biochem-062917-012023.

Peng, M., Shahzad, R., Gul, A., Subthain, H., Shen, S., Lei, L., Zheng, Z., Zhou, J., Lu, D., Wang, S., et al. (2017). Differentially evolved glucosyltransferases determine natural variation of rice flavone accumulation and UV-tolerance. Nat. Commun. **8**:1975. https://doi.org/10.1038/s41467-017-02168-x.

Peng, Z., Song, L., Chen, M., Liu, Z., Yuan, Z., Wen, H., Zhang, H., Huang, Y., Peng, Z., Yang, H., et al. (2024). Neofunctionalization of an *OMT* cluster dominates polymethoxyflavone biosynthesis associated with the domestication of citrus. Proc. Natl. Acad. Sci. USA **121**:e2321615121. https://doi.org/10.1073/pnas.2321615121.

Purcell, S., Neale, B., Todd-Brown, K., Thomas, L., Ferreira, M.A.R., Bender, D., Maller, J., Sklar, P., de Bakker, P.I.W., Daly, M.J., and Sham, P.C. (2007). PLINK: a tool set for whole-genome association and population-based linkage analyses. Am. J. Hum. Genet. **81**:559–575. https://doi.org/10.1086/519795.

Shang, Y., Ma, Y., Zhou, Y., Zhang, H., Duan, L., Chen, H., Zeng, J., Zhou, Q., Wang, S., Gu, W., et al. (2014). Biosynthesis, regulation, and domestication of bitterness in cucumber. Science **346**:1084–1088. https://doi.org/10.1126/science.1259215.

Shen, N., Wang, T., Gan, Q., Liu, S., Wang, L., and Jin, B. (2022). Plant flavonoids: classification, distribution, biosynthesis, and antioxidant activity. Food Chem. **383**:132531. https://doi.org/10.1016/j.foodchem.2022.132531.

Soskine, M., and Tawfik, D.S. (2010). Mutational effects and the evolution of new protein functions. Nat. Rev. Genet. **11**:572–582. https://doi.org/10.1038/nrg2808.

Stamatakis, A. (2006). RAxML-VI-HPC: maximum likelihood-based phylogenetic analyses with thousands of taxa and mixed models. Bioinformatics **22**:2688–2690. https://doi.org/10.1093/bioinformatics/btl446.

Sugimoto, K., Zager, J.J., Aubin, B.S., Lange, B.M., and Howe, G.A. (2022). Flavonoid deficiency disrupts redox homeostasis and terpenoid biosynthesis in glandular trichomes of tomato. Plant Physiol. **188**:1450–1468. https://doi.org/10.1093/plphys/kiab488.

Tan, F.-F., Zhu, R., Xiong, B., Zhang, G.-M., Zhao, W., and Jia, K.-Z. (2022). Engineering the entrance of a flavonoid glycosyltransferase promotes the glycosylation of etoposide aglycone. ACS Synth. Biol. **11**:1874–1880. https://doi.org/10.1021/acssynbio.2c00032.

Tanaka, Y., Sasaki, N., and Ohmiya, A. (2008). Biosynthesis of plant pigments: anthocyanins, betalains and carotenoids. Plant J. **54**:733–749. https://doi.org/10.1111/j.1365-313X.2008.03447.x.

Tewksbury, J.J., Reagan, K.M., Machnicki, N.J., Carlo, T.A., Haak, D.C., Peñaloza, A.L.C., and Levey, D.J. (2008). Evolutionary ecology of pungency in wild chilies. Proc. Natl. Acad. Sci. USA **105**:11808–11811. https://doi.org/10.1073/pnas.0802691105.

Thodberg, S., Del Cueto, J., Mazzeo, R., Pavan, S., Lotti, C., Dicenta, F., Jakobsen Neilson, E.H., Møller, B.L., and Sánchez-Pérez, R. (2018). Elucidation of the amygdalin pathway reveals the metabolic basis of bitter and sweet almonds (*Prunus dulcis*). Plant Physiol. **178**:1096–1111. https://doi.org/10.1104/pp.18.00922.

Trott, O., and Olson, A.J. (2010). AutoDock Vina: improving the speed and accuracy of docking with a new scoring function, efficient optimization, and multithreading. J. Comput. Chem. **31**:455–461. https://doi.org/10.1002/jcc.21334.

Trunschke, J., Lunau, K., Pyke, G.H., Ren, Z.X., and Wang, H. (2021). Flower color evolution and the evidence of pollinator-mediated selection. Front. Plant Sci. **12**:617851. https://doi.org/10.3389/fpls.2021.617851.

Wang, Y., Liu, X.J., Chen, J.B., Cao, J.P., Li, X., and Sun, C.D. (2022). Citrus flavonoids and their antioxidant evaluation. Crit. Rev. Food Sci. Nutr. **62**:3833–3854. https://doi.org/10.1080/10408398.2020.1870035.

Wang, Y., Tang, H., DeBarry, J.D., Tan, X., Li, J., Wang, X., Lee, T.h., Jin, H., Marler, B., Guo, H., et al. (2012). MCScanX: a toolkit for detection and evolutionary analysis of gene synteny and collinearity. Nucleic Acids Res. **40**:e49. https://doi.org/10.1093/nar/gkr1293.

Wilson, A.E., and Tian, L. (2019). Phylogenomic analysis of UDP-dependent glycosyltransferases provides insights into the evolutionary landscape of glycosylation in plant metabolism. Plant J. **100**:1273–1288. https://doi.org/10.1111/tpj.14514.

Wu, G.A., Terol, J., Ibanez, V., López-García, A., Pérez-Román, E., Borredá, C., Domingo, C., Tadeo, F.R., Carbonell-Caballero, J.,

Alonso, R., et al. (2018). Genomics of the origin and evolution of *Citrus*. Nature **554**:311–316. https://doi.org/10.1038/nature25447.

Yang, J., Chen, R., Wang, C., Li, C., Ye, W., Zhang, Z., and Wang, S. (2024). A widely targeted metabolite modificomics strategy for modified metabolites identification in tomato. J. Integr. Plant Biol. **66**:810–823. https://doi.org/10.1111/jipb.13629.

Yuan, Z., Li, G., Zhang, H., Peng, Z., Ding, W., Wen, H., Zhou, H., Zeng, J., Chen, J., and Xu, J. (2024). Four novel *Cit7GlcTs* functional in flavonoid 7-*O*-glucoside biosynthesis are vital to flavonoid biosynthesis shunting in citrus. Hortic. Res. **11**:uhae098. https://doi.org/10.1093/hr/uhae098.

Zhang, M., Yi, Y., Gao, B.H., Su, H.F., Bao, Y.O., Shi, X.M., Wang, H.D., Li, F.D., Ye, M., and Qiao, X. (2022). Functional characterization and protein engineering of a triterpene 3-/6-/2′-*O*-glycosyltransferase reveal a conserved residue critical for the regiospecificity. Angew. Chem. Int. Ed. Engl. **61**:e202113587. https://doi.org/10.1002/anie.202113587.

Zong, G., Fei, S., Liu, X., Li, J., Gao, Y., Yang, X., Wang, X., and Shen, Y. (2019). Crystal structures of rhamnosyltransferase UGT89C1 from *Arabidopsis thaliana* reveal the molecular basis of sugar donor specificity for UDP-β-l-rhamnose and rhamnosylation mechanism. Plant J. **99**:257–269. https://doi.org/10.1111/tpj.14321.

Zust, T., and Agrawal, A.A. (2017). Trade-offs between plant growth and defense against insect herbivory: an emerging mechanistic synthesis. Annu. Rev. Plant Biol. **68**:513–534. https://doi.org/10.1146/annurev-arplant-042916-040856.

Plant Communications, Volume 7

Supplemental information

Evolutionary origin of neohesperidoside, a bitter metabolite, and its potential role in biotic defense and citrus dissemination

Gu Li, Huan Wen, Hanxin Zhou, Yuan Liu, Ziyu Yuan, Huixian Zhang, Zhehui Hu, Zeyang Liu, Huili Ma, Qi Chen, Guixiang Chen, Jia-Long Yao, Juan Xu, and Jiajing Chen

Supporting Information for

Emergence of the Bitter Metabolite Neohesperidoside and Its Potential Role in Biotic Defense and Citrus Dissemination

Gu Li [a,b,c], Huan Wen [a], Hanxin Zhou [a], Yuan Liu [a], Ziyuyuan [a], Huixian Zhang [a], Zhehui Hu [a], Zeyang Liu [a], Huili Ma [a], Qi Chen [a],Guixiang Chen [a], Jia-Long Yao [b], Juan Xu [a,c,d*], Jiajing Chen [a,c,d*]

[a] National Key Laboratory for Germplasm Innovation & Utilization of Horticultural Crops, College of Horticulture and Forestry Sciences, Huazhong Agricultural University, Wuhan, 430070, P.R. China. Hainan Research Institute of Huazhong Agricultural University, Sanya 572025, P.R. China.

[b] New Zealand Institute for Bioeconomy Science Limited, Private Bag 92169, Auckland 1142, New Zealand

[c] Hubei Hongshan Laboratory, Wuhan, 430070, P.R. China.

[d] Sensory Evaluation and Quality Analysis Centre of Horticultural Products, Huazhong Agricultural University, Wuhan 430070, China

*Correspondence: chenjiajing@mail.hzau.edu.cn (Jiajing Chen), xujuan@mail.hzau.edu.cn (Juan Xu)

This PDF file includes:

Supplemental Figures 1 to 22

Supplemental Tables 1 to 6

Other supporting materials for this manuscript include the following:

Supplemental Data 1. Summary of flavonoid compounds detected in mature fruit flavedo of thirty-five citrus accessions.

Supplemental Data 2. Concentrations (mg/g, DW) of the major Neo and Rut in citrus germplasms analyzed in this study.

Supplemental Data 3. Amplification analysis statistics of group A genes in *C. grands* (L.) Osbeck.cv. 'Wanbaiyou'.

Supplemental Data 4. Micro-synteny relationships among genes from species in *Citrus*-related genera, early-diverging citrus and other species.

Supplemental Data 5. Analysis of gene presence and absence for 135 sequenced genomes from citrus accessions and relatives.

Supplemental Data 6. Geographical occurrences of 341 *Citrus*-related genera and 439 early-diverging citrus samples.

Supplemental Data 7. Whole-genome resequencing metadata for 70 accessions used in population genomic analysis.

Supplemental Data 8. Limonoids content in different tissues of fifty-five citrus accessions.

Supplemental Data 9. Primers used in this study.

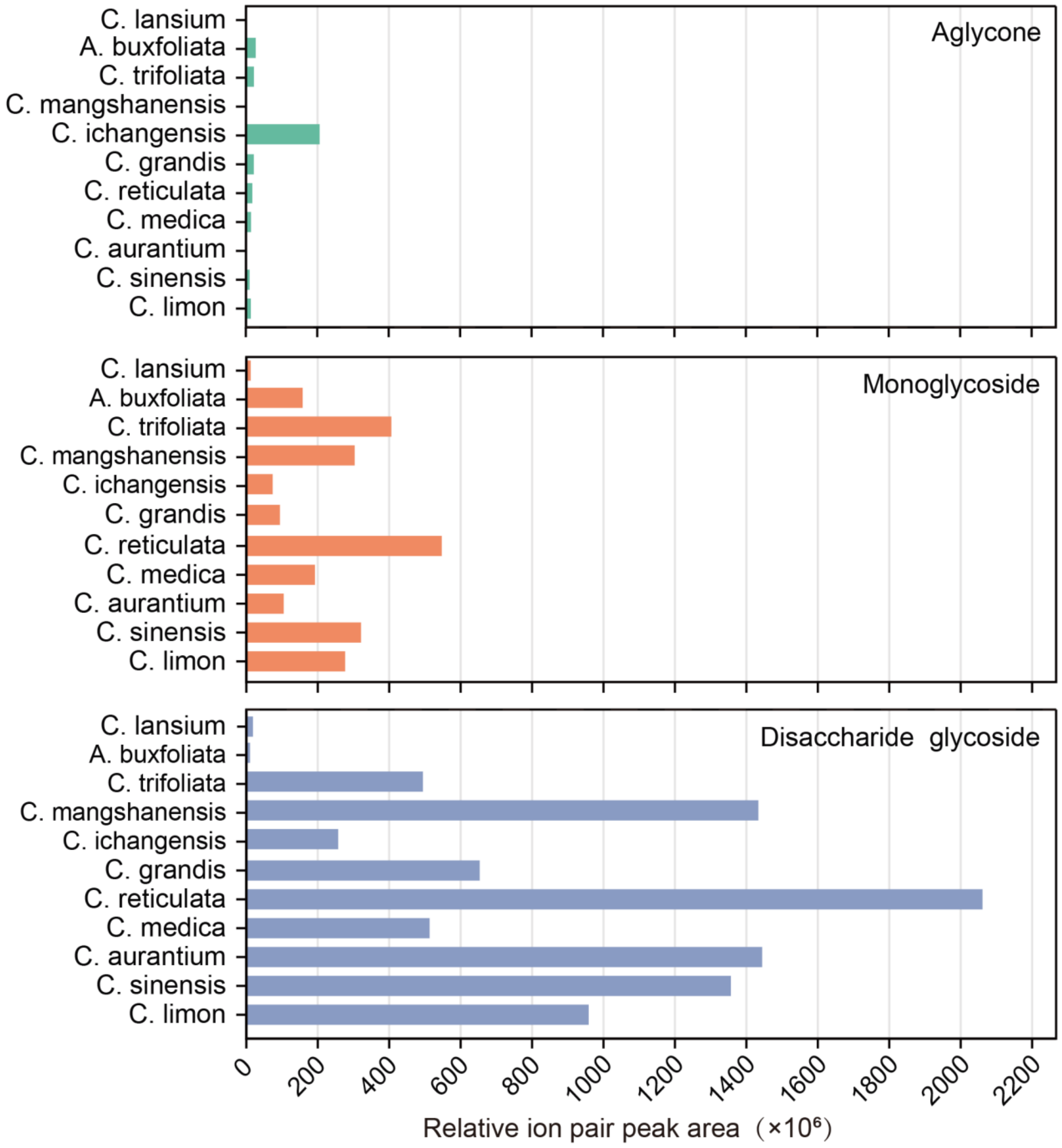

Supplemental Figure 1. Accumulation of flavonoid compounds in the fruit flavedo of various citrus species. Flavonoid metabolites were classified into aglycones, monoglycosides, and disaccharide glycosides. Each bar represents the summed peak areas of all detected compounds within each class in each species, as determined by LC-MS/MS. Colors represent different compound categories. Detailed compound information is listed in Dataset 1.

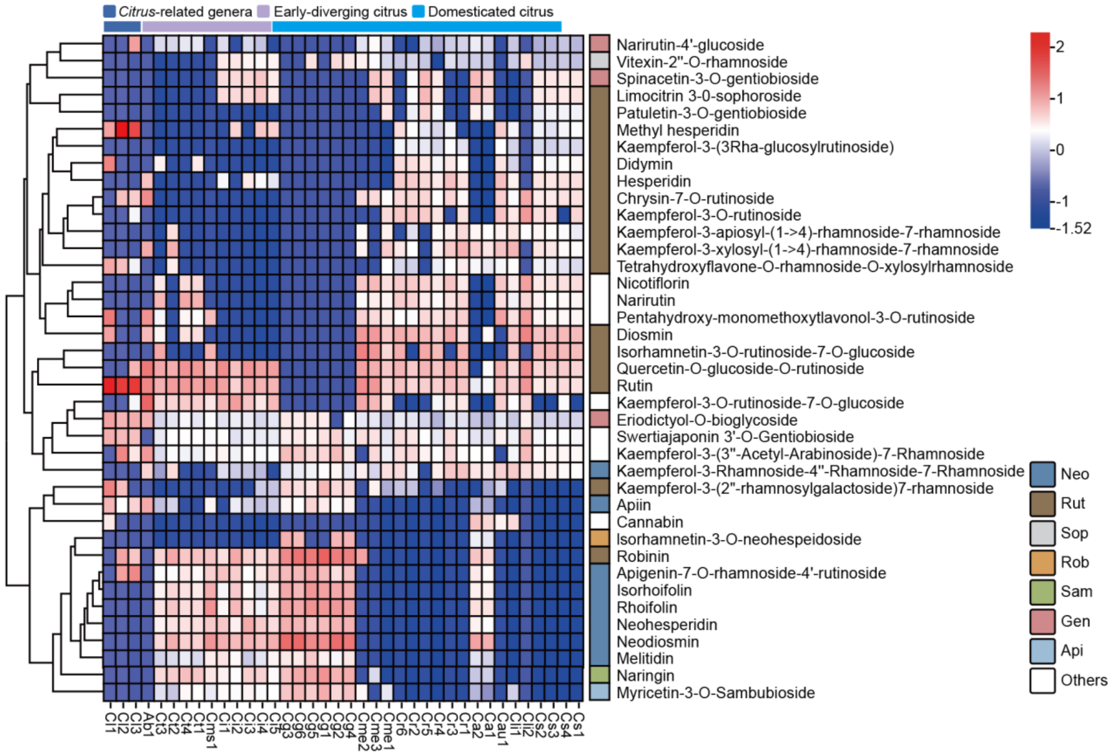

Supplemental Figure 2. Heatmap of flavonoid disaccharide glycosides among 38 citrus accessions. Each row represents a compound, and each column represents a citrus accession. Color intensity indicates relative metabolite abundance, with values standardized by Z-score transformation across accessions (column-wise normalization). The top annotation indicates the classification of citrus accessions. The right annotation denotes the type of disaccharide glycoside; compounds that could not be assigned to the seven defined types are grouped as ‘Others’. Neo, neohesperidoside; Rut, rutinoside; Sop, sophoroside; Rob, robinobioside; Sam, sambubioside; Gen, gentiobioside; Api, apiosylrhamnoside.

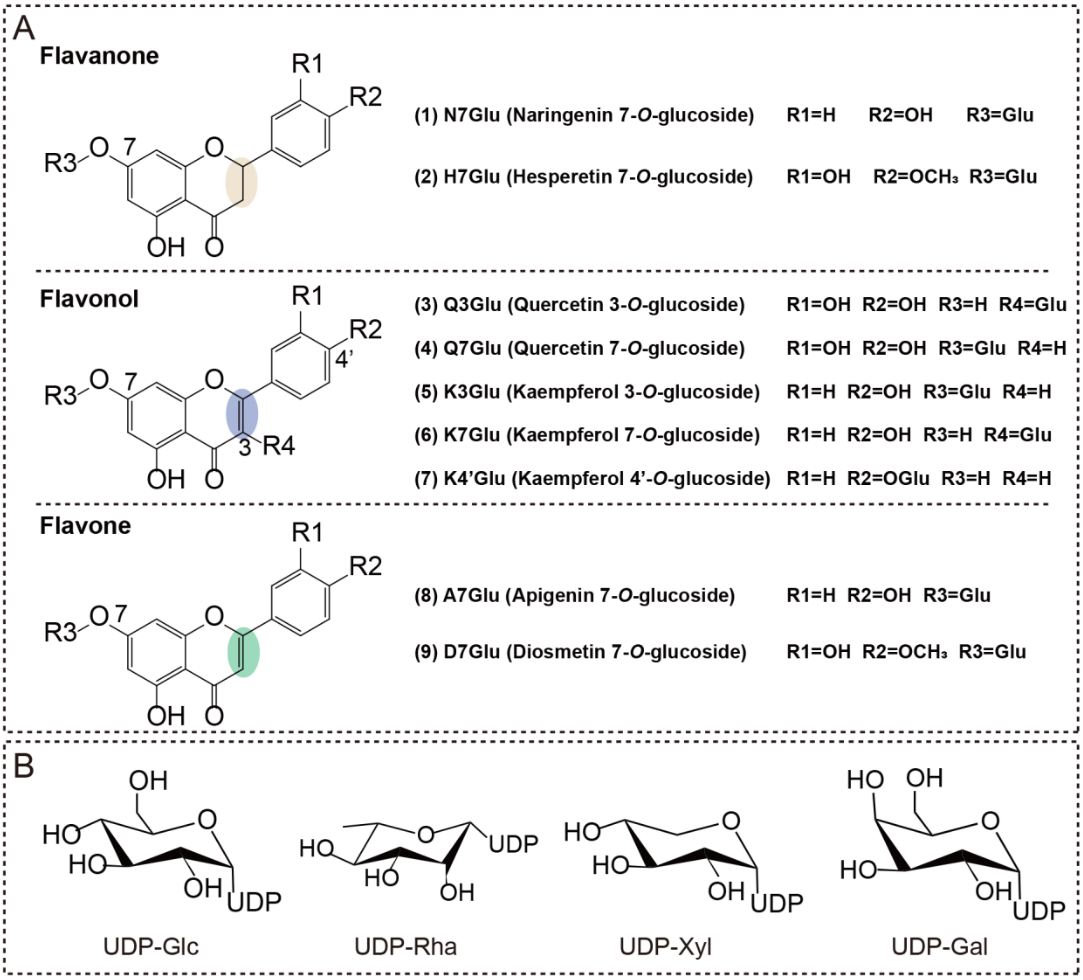

Supplemental Figure 3. Structures of flavonoid substrates and sugar donors used for functional assays. (A) Chemical structures and abbreviations of flavonoid substrates used for in vitro functional verification. Color blocks represent the structural differences on the core skeleton among the three flavonoid subgroups. (B) Chemical structures of sugar donors in the assays.

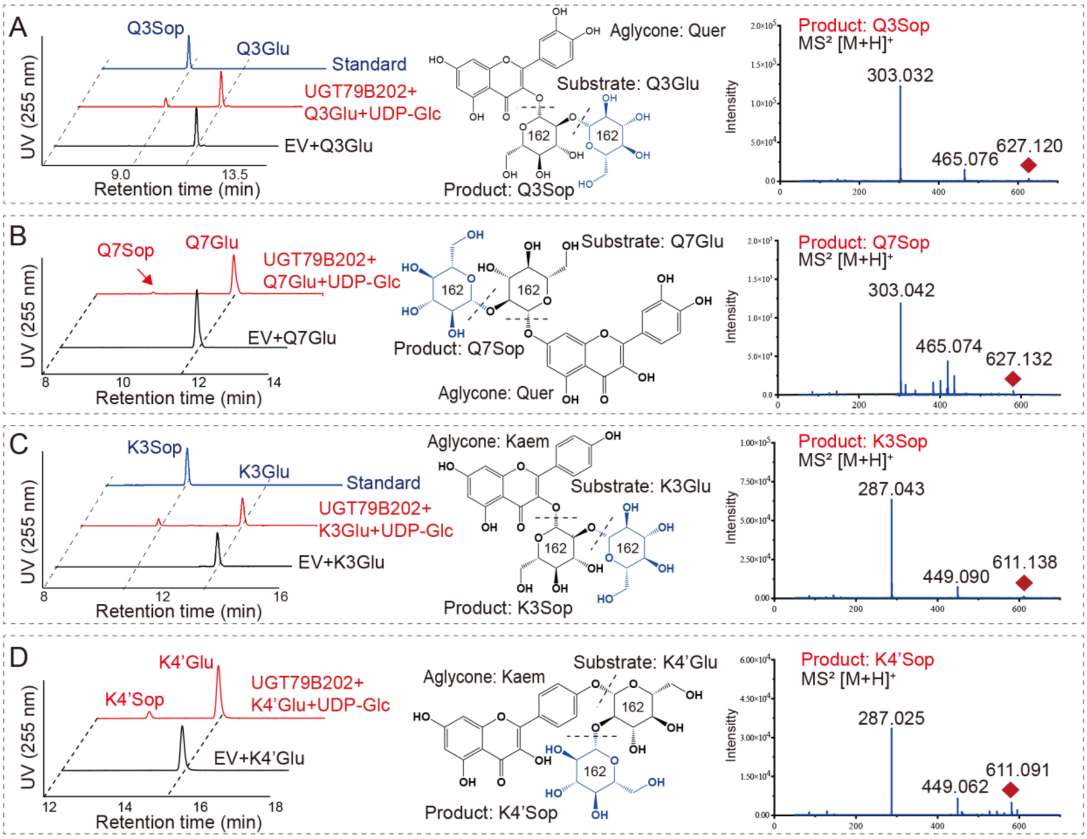

Supplemental Figure 4. Identification of enzymatic products catalyzed by UGT79B202 using flavonol mono-glucosides and UDP-Glc as substrates. (A-D) HPLC chromatograms, chemical structures of reaction products, and LC/MS fragmentation spectra (positive ion mode) for reaction using Q3Glu (A), Q7Glu (B), K3Glu (C) and K4'Glu (D) used as substrate. In chromatograms, blue traces represent authentic standards, red traces represent reactions with UGT79B202, and black traces correspond to empty vector (EV) controls. The glycosyl moiety transferred by the enzyme is highlighted in blue in the product structures. In MS/MS spectra, precursor ions are marked with red diamonds. Quer, quercetin; Q3Glu, quercetin 3-*O*-glucoside; Q3Sop, quercetin 3-*O*-sophoroside; Q7Glu, quercetin 7-*O*-glucoside; Q7Sop, quercetin 7-*O*-sophoroside; Kaem, kaempferol; K3Glu, kaempferol 3-*O*-glucoside; K3Sop, kaempferol 3-*O*-sophoroside; K4'Glu, kaempferol 4'-*O*-glucoside; K4'Sop, kaempferol 4'-*O*-sophoroside. EV, empty vector pMAL-c2x.

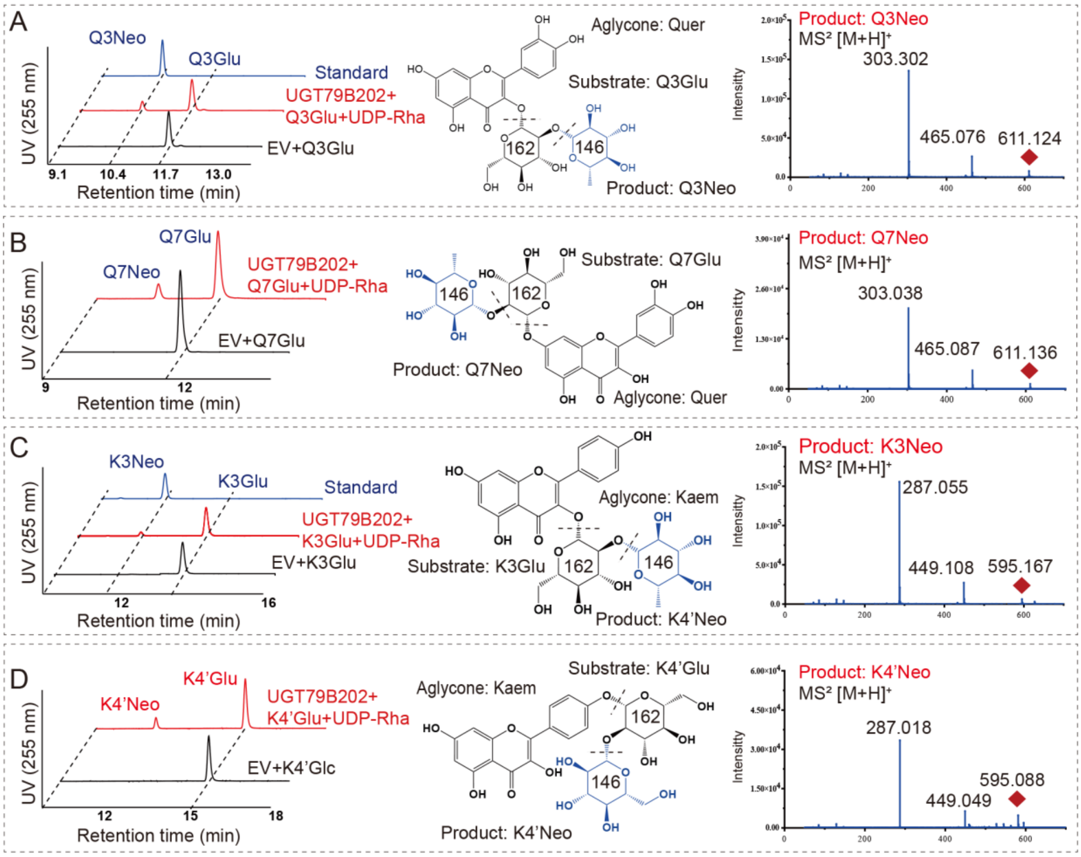

Supplemental Figure 5. Identification of enzymatic products catalyzed by UGT79B202 using flavonol mono-glucosides and UDP-Rha as substrates. (A-D) HPLC chromatograms, chemical structures of reaction products, and LC/MS fragmentation spectra (positive ion mode) for reaction using Q3Glu (A), Q7Glu (B), K3Glu (C) and K4'Glu (D) used as substrate. In chromatograms, blue traces represent authentic standards, red traces represent reactions with UGT79B202, and black traces correspond to empty vector (EV) controls. The glycosyl moiety transferred by the enzyme is highlighted in blue in the product structures. In MS/MS spectra, precursor ions are marked with red diamonds. Quer, quercetin; Q3Glu, quercetin 3-*O*-glucoside; Q3Neo, quercetin 3-*O*-neohesperidoside; Q7Glu, quercetin 7-*O*-glucoside; Q7Neo, quercetin 7-*O*-neohesperidoside; Kaem, kaempferol; K3Glu, kaempferol 3-*O*-glucoside; K3Neo, kaempferol 3-*O*-neohesperidoside; K4'Glu, kaempferol 4'-*O*-glucoside; K4'Neo, kaempferol 4'-*O*-neohesperidoside. EV, empty vector pMAL-c2x.

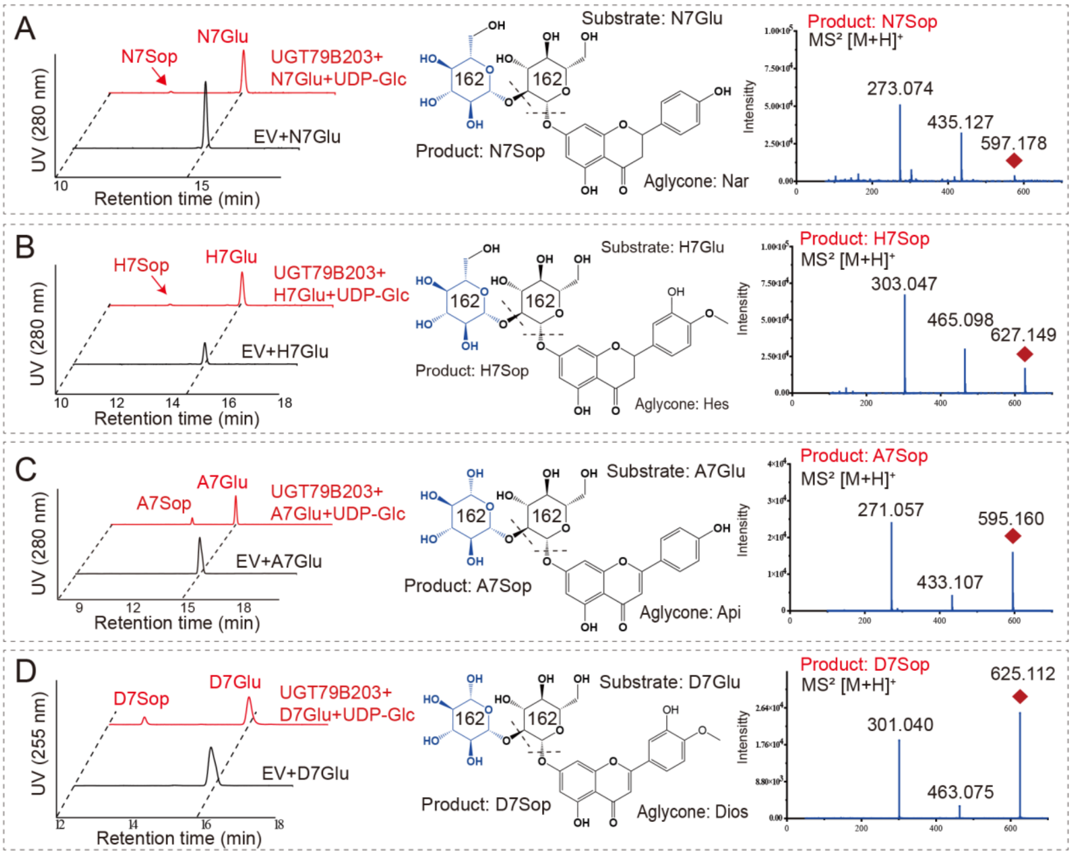

Supplemental Figure 6. Identification of enzymatic products catalyzed by UGT79B203 using flavanone and flavone mono-glucosides and UDP-Glc as substrates. (A-D) HPLC chromatograms, chemical structures of reaction products, and MS/MS fragmentation spectra (positive ion mode) for reactions using N7Glu (A), H7Glu (B), A7Glu (C), and D7Glu (D) used as substrate. In chromatograms, blue traces represent authentic standards, red traces represent reactions with UGT79B202, and black traces correspond to empty vector (EV) controls. The glycosyl moiety transferred by the enzyme is highlighted in blue in the product structures. In MS/MS spectra, precursor ions are marked with red diamonds. Nar, naringenin; N7Glu, naringenin 7-*O*-glucoside; N7Sop, naringenin 7-*O*-sophoroside; Hes, hesperetin; H7Glu, hesperetin 7-*O*-glucoside; H7Sop, hesperetin 7-*O*-sophoroside; Api, apigenin; A7Glu, apigenin 7-*O*-glucosid; A7Sop, apigenin 7-*O*-sophoroside; Dios, diosmetin; D7Glu, diosmetin 7-*O*-glucoside; D7Sop, diosmetin 7-*O*-sophoroside. EV, empty vector pMAL-c2x.

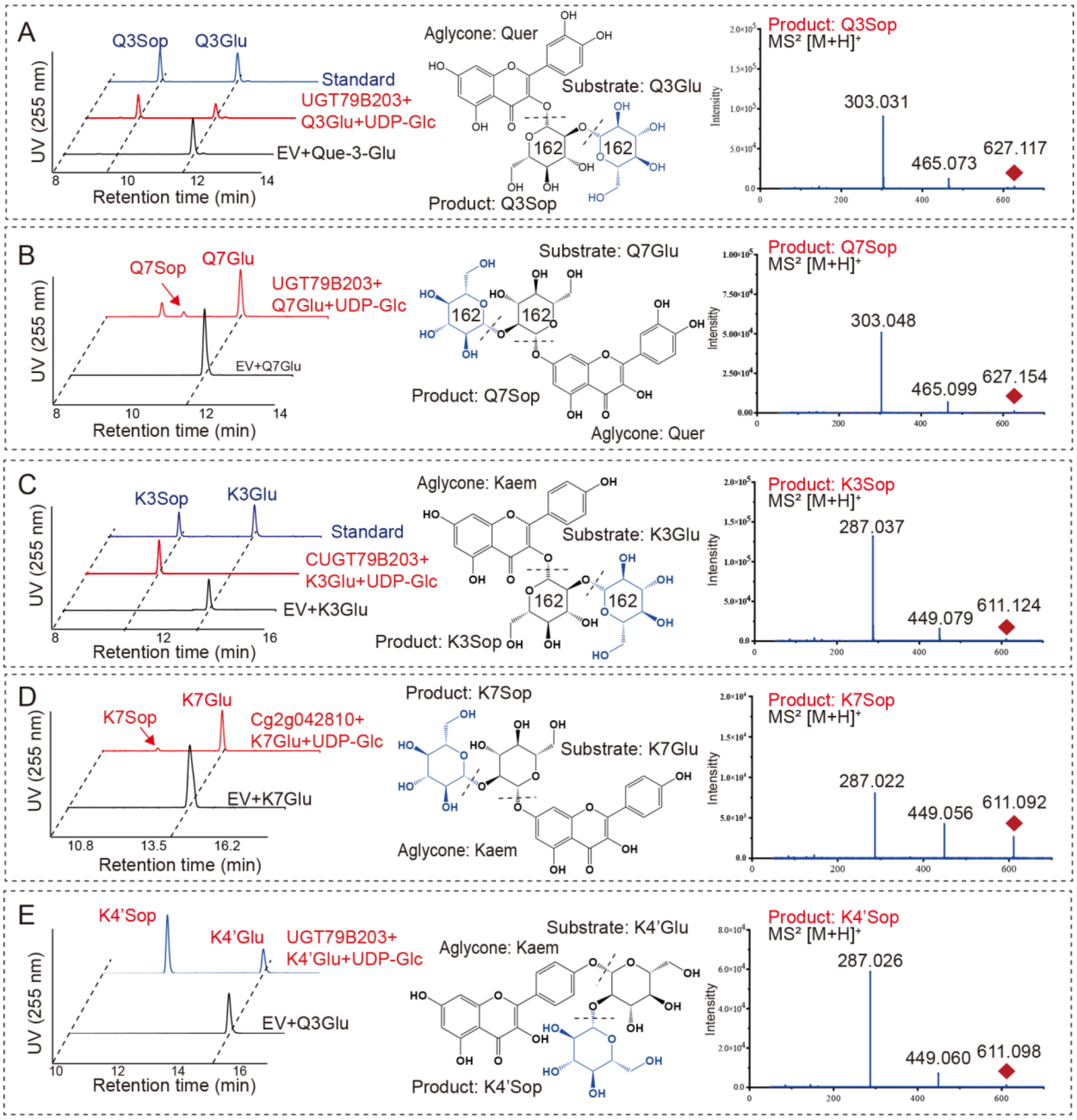

Supplemental Figure 7. Identification of enzymatic products catalyzed by UGT79B203 using flavonol mono-glucosides and UDP-Glc as substrates. (A-E) HPLC chromatograms, chemical structures of reaction products, and MS/MS fragmentation spectra (positive ion mode) for reactions using Q3Glu (A), Q7Glu (B), K3Glu (C), K7Glu (D) and K4'Glu (E) used as substrate. In chromatograms, blue traces represent authentic standards, red traces represent reactions with UGT79B202, and black traces correspond to empty vector (EV) controls. The glycosyl moiety transferred by the enzyme is highlighted in blue in the product structures. In MS/MS spectra, precursor ions are marked with red diamonds. Que, quercetin; Q3Glu, quercetin 3-*O*-glucoside; Q3Sop, quercetin 3-*O*-sophoroside; Q7Glu, quercetin 7-*O*-glucoside; Q7Sop, quercetin 7-*O*-sophoroside; Kaem, kaempferol; K3Glu, kaempferol 3-*O*-glucoside; K3Sop, kaempferol 3-*O*-sophoroside; K7Glu, kaempferol 7-*O*-glucoside; K7Sop, kaempferol 7-*O*-sophoroside; K4'Glu, kaempferol 4'-*O*-glucoside; K4'Sop, kaempferol 4'-*O*-sophoroside. EV, empty vector pMAL-c2x.

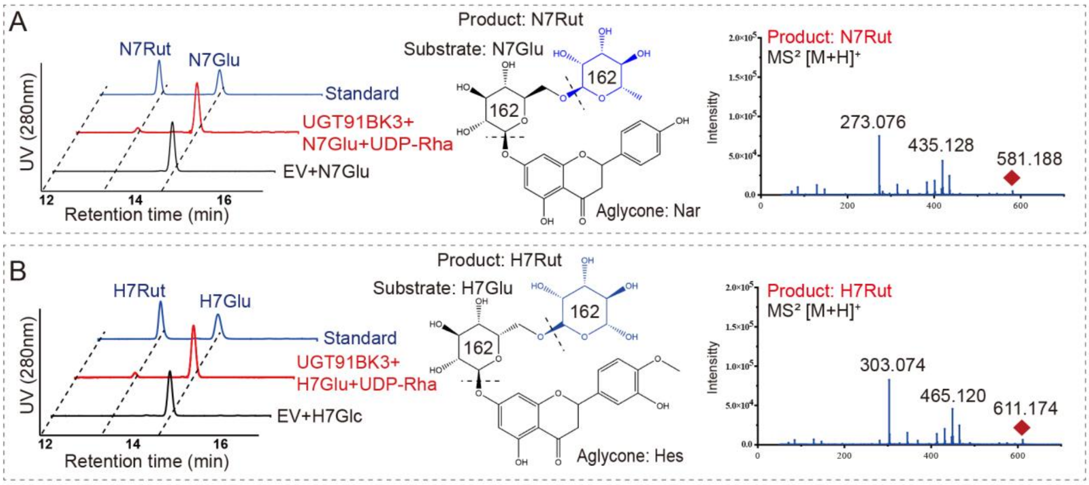

Supplemental Figure 8. Identification of enzymatic products catalyzed by UGT91BK3 using flavanone mono-glucosides and UDP-Rha as substrates. (A-B) HPLC chromatograms, chemical structures of reaction products, and MS/MS fragmentation spectra (positive ion mode) for reactions using N7Glu (A) and H7Glu (B) as substrates. In the chromatograms, blue traces represent authentic standards, red traces represent reactions with UGT91BK3, and black traces correspond to empty-vector (EV) controls. The transferred sugar moiety is highlighted in blue in the product structures. In MS/MS spectra, precursor ions are marked with red diamonds. N7Rut, naringenin 7-*O*-rutinoside; N7Glu, naringenin 7-*O*-glucoside; H7Rut, hesperetin 7-*O*-rutinoside; H7Glu, hesperetin 7-*O*-glucoside. EV, empty vector pMAL-c2x.

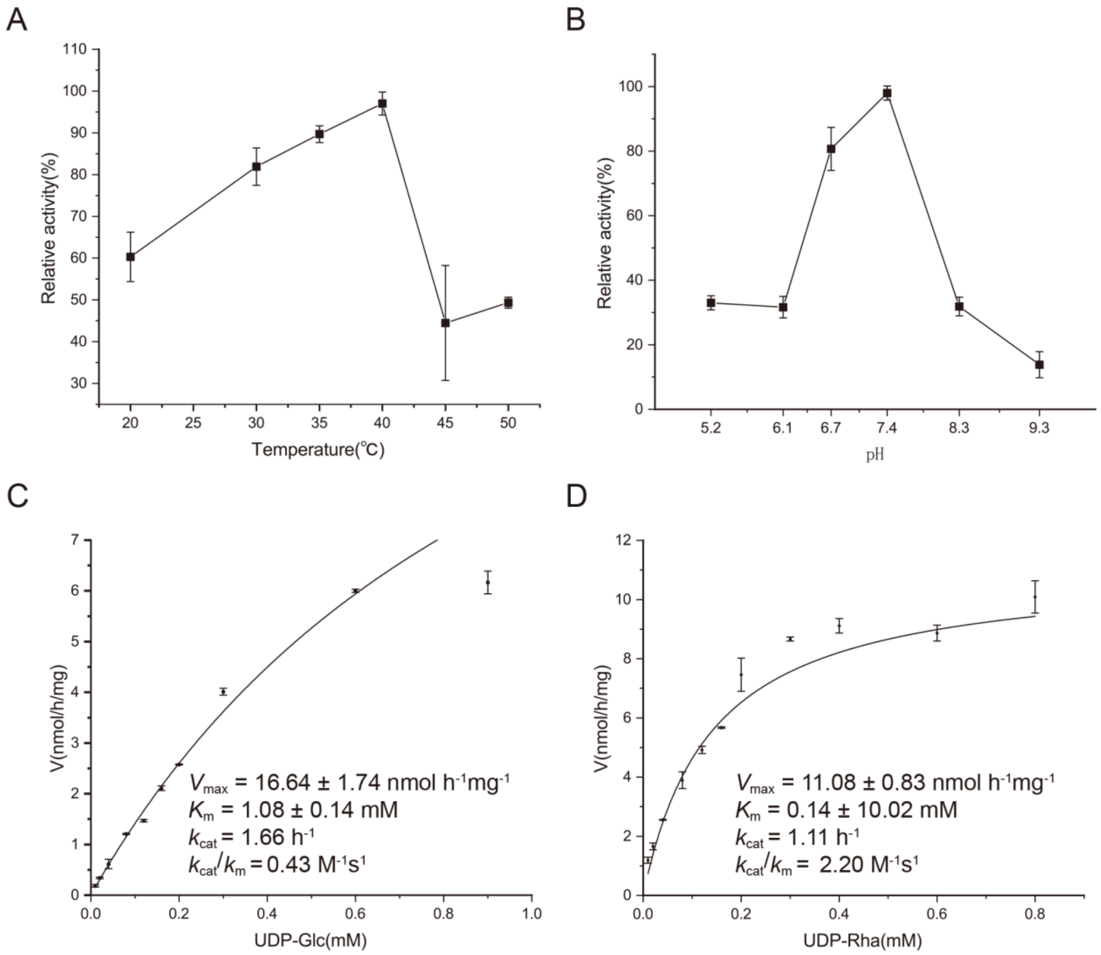

Supplemental Figure 9. Biochemical characterization of UGT79B202. (A and B) Effects of temperature (A) and pH (B) on enzyme activity. (C and D) Kinetic analysis with UDP-Glc (C) and UDP-Rha (D) as the sugar donor. All assays were performed in triplicate (mean ± SD).

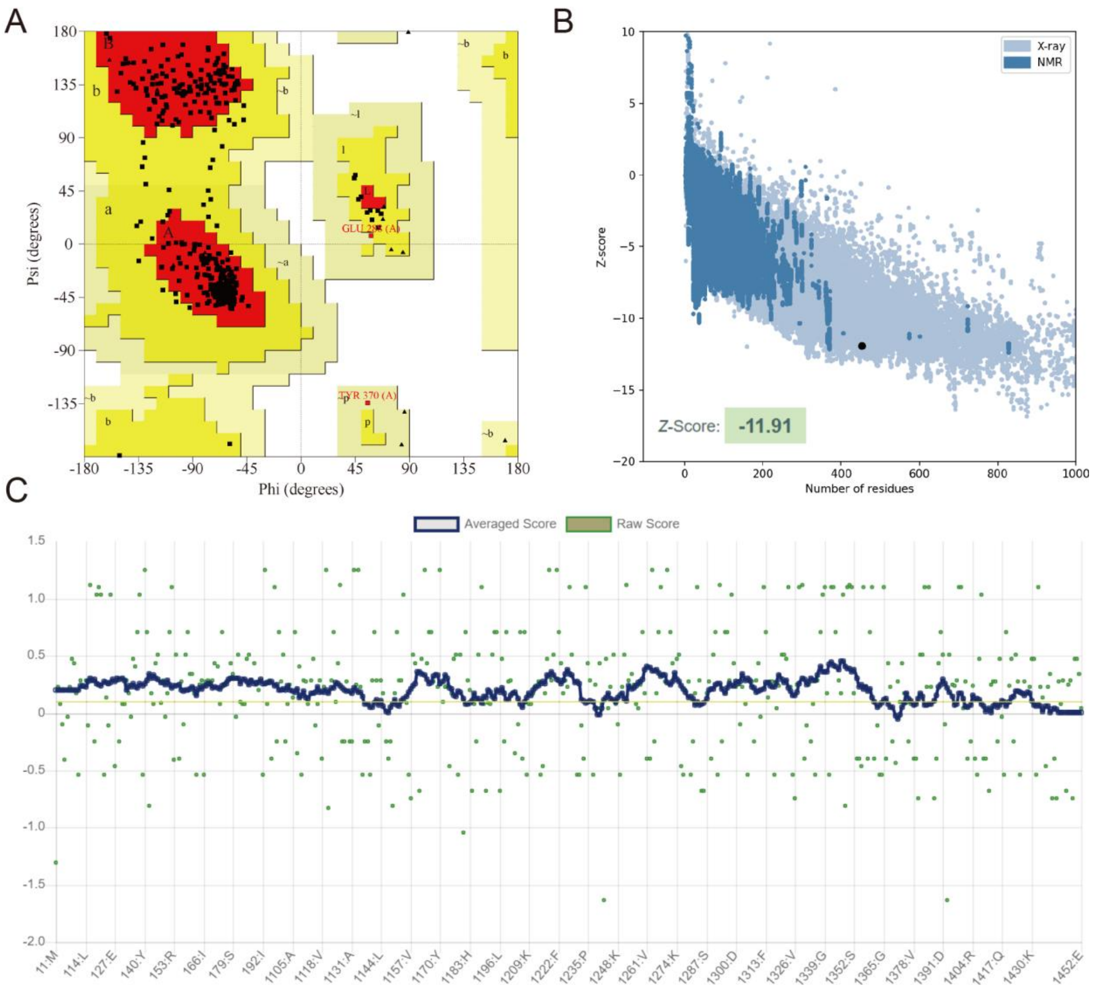

Supplemental Figure 10. Protein conformational rationality assessment of Cm1,2RhaT. (A) The Ramachandran plot analysis of Cm1,2RhaT protein (86.7% most favored, 13.1% additional allowed, 0.3% generously allowed, 0.0% disallowed). (B) Evaluation results of ProSA for Cm1,2RhaT protein. The overall model Z-score of -11.91 lies well within the range of scores typically found for native proteins of similar size determined by X-ray crystallography (light blue area). (C) The 3D-1D score plots of each residue in Cm1,2RhaT. 83.85% of the residues have averaged 3D-1D score >= 0.1.

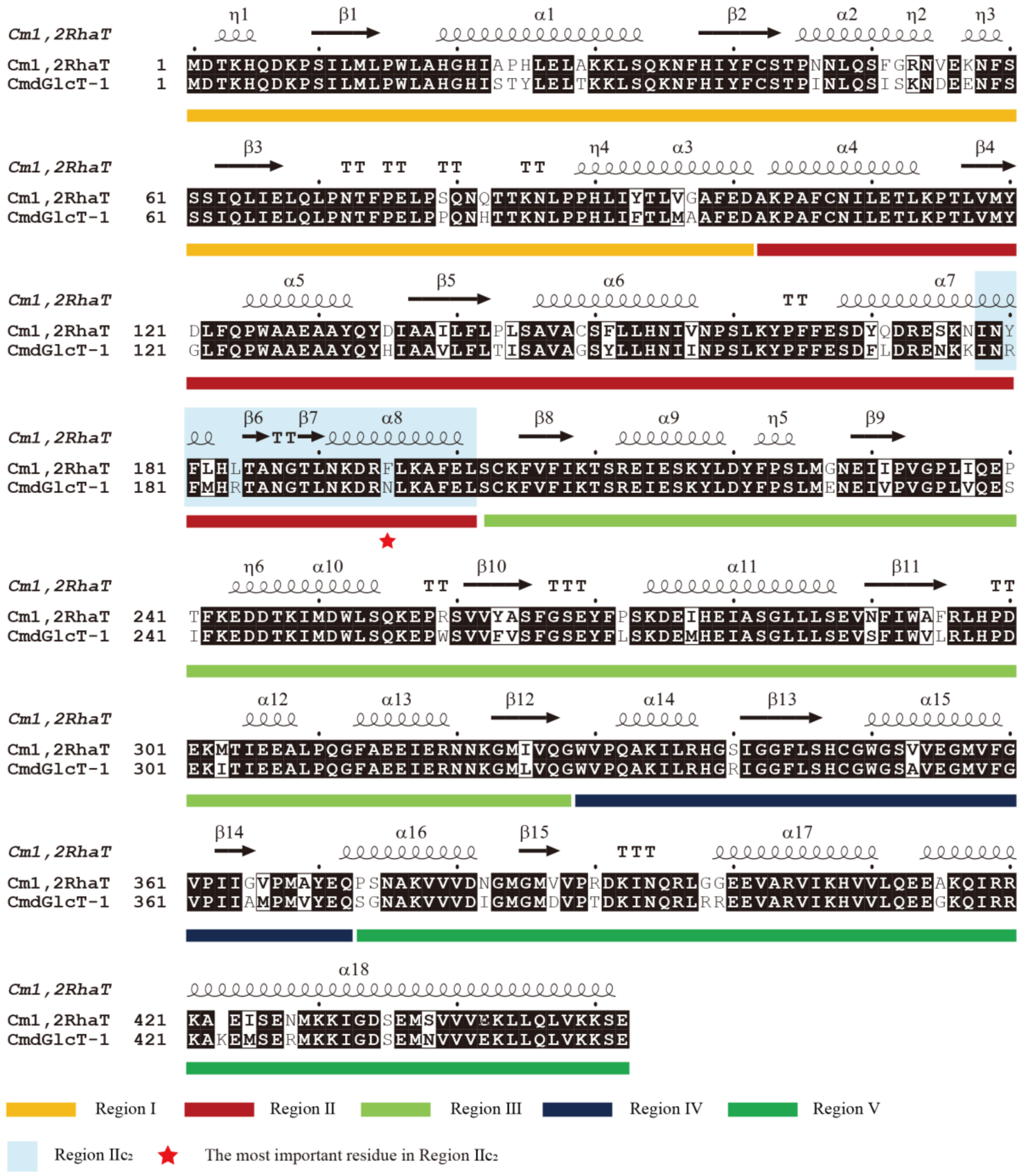

Supplemental Figure 11. Sequence alignment and region division of Cm1,2RhaT and CmdGlcT-1. Amino acid sequences of Cm1,2RhaT and CmdGlcT-1 were aligned and annotated with predicted secondary structural elements, including α-helices (α), β-strands (β), η-helices (η), and turns (T). Five regions (Region I–V) were defined based on sequence segmentation. Region IIc2, which plays a key role in determining sugar donor specificity, is highlighted with a blue color block, and the critical amino acid residue Phe195 is marked with a red five-pointed star.

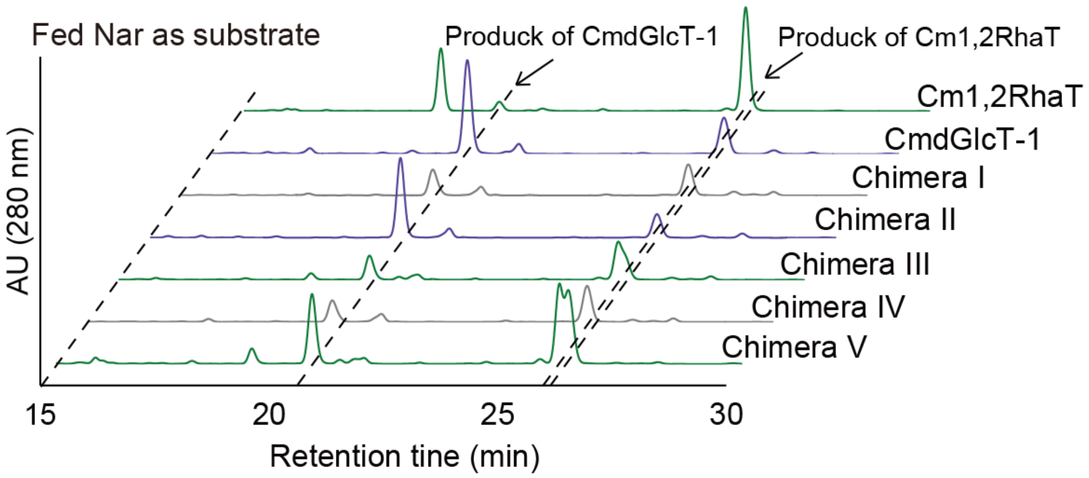

Supplemental Figure 12. Functional analysis of chimeric proteins in BY2 transgenic cell using Nar as substrate. Nar, narigenin.The dashed line represents a characteristic peak used to distinguish compounds with similar retention times. To better illustrate the functional differences, chromatograms are color-coded: green indicates proteins exhibiting 1,2RhaT activity, purple indicates proteins with dGlcT activity, and gray indicates proteins lacking both activities.

.

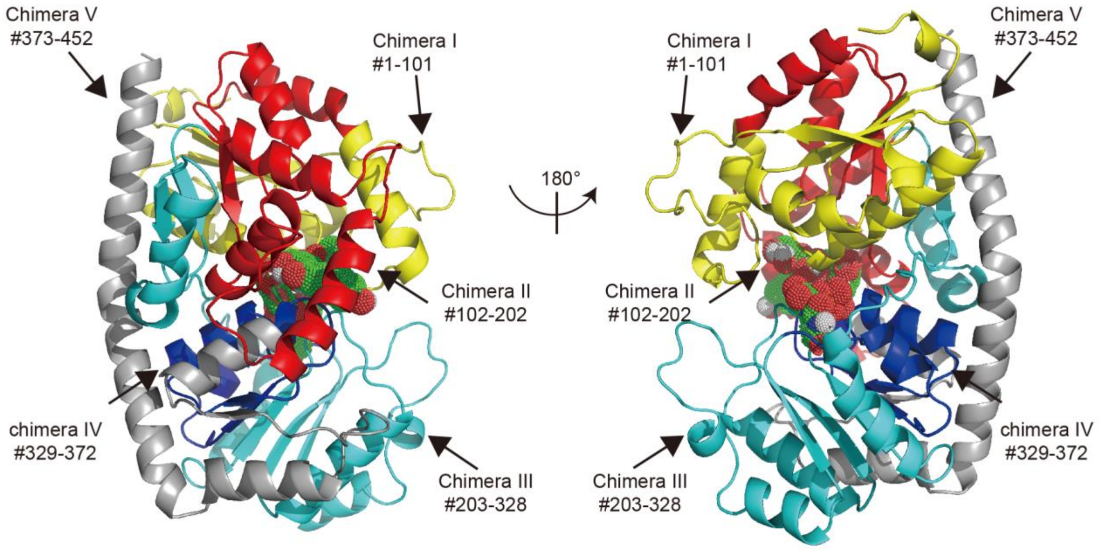

Supplemental Figure 13. Schematic representation of region division in Cm1,2RhaT protein. The protein is divided into five regions (Region I–V), with the corresponding amino acid positions indicated beside each segment. The docked substrate molecules are shown as dots.

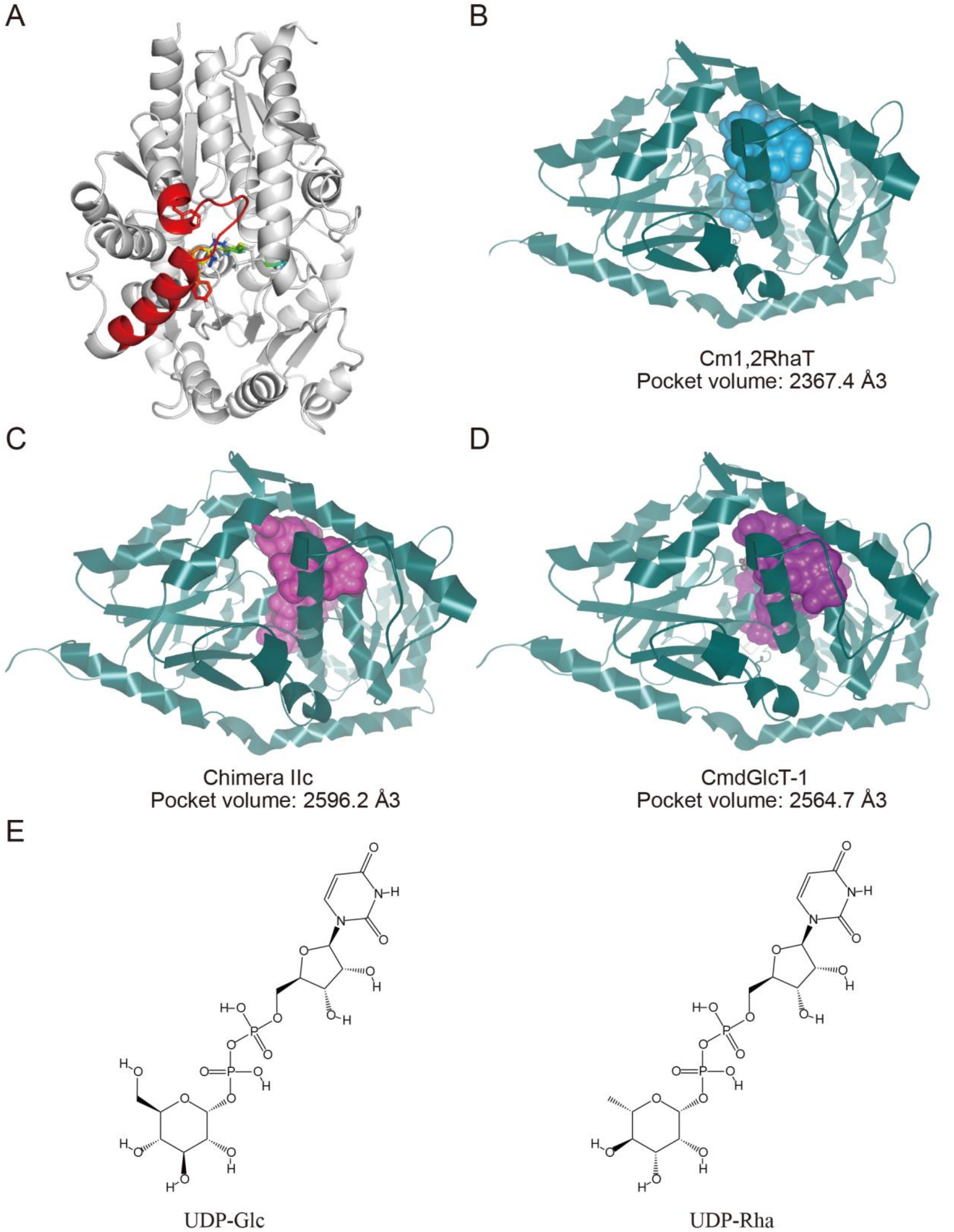

Supplemental Figure 14. Comparison of pocket sizes among Cm1,2RhaT, Chimeric IIc, and CmdGlcT-1. (A) Location of the region IIc (highlighted in red) on the Cm1,2RhaT structure. (B-D) Predicted pocket size of Cm1,2RhaT (B), Chimera IIc (C) and CmdGlcT-1 (D). (E) Chemical structures of the sugar donors UDP-glucose (UDP-Glc) and UDP-rhamnose (UDP-Rha). Functional assays showed that substitution of region IIc led to a shift from rhamnosylation to glucosylation, demonstrating that region IIc plays a critical role in determining sugar donor preference.

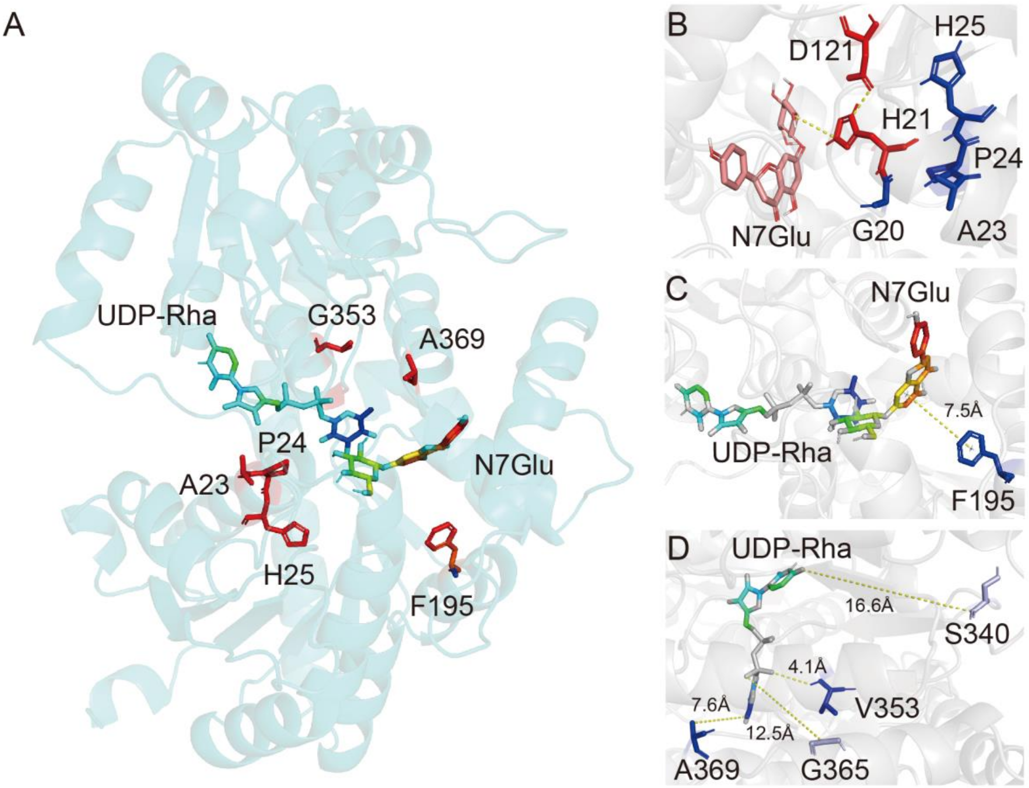

Supplemental Figure 15. Structural location of key differential amino acid residues identified by domain swapping. (A) The location of the key differential amino acid residues onto the Cm1,2RhaT protein structure. (C-E) Structural location and/or distance of key differential residues within region I (C), region II (D) and region V (E) of Cm1,2RhaT.

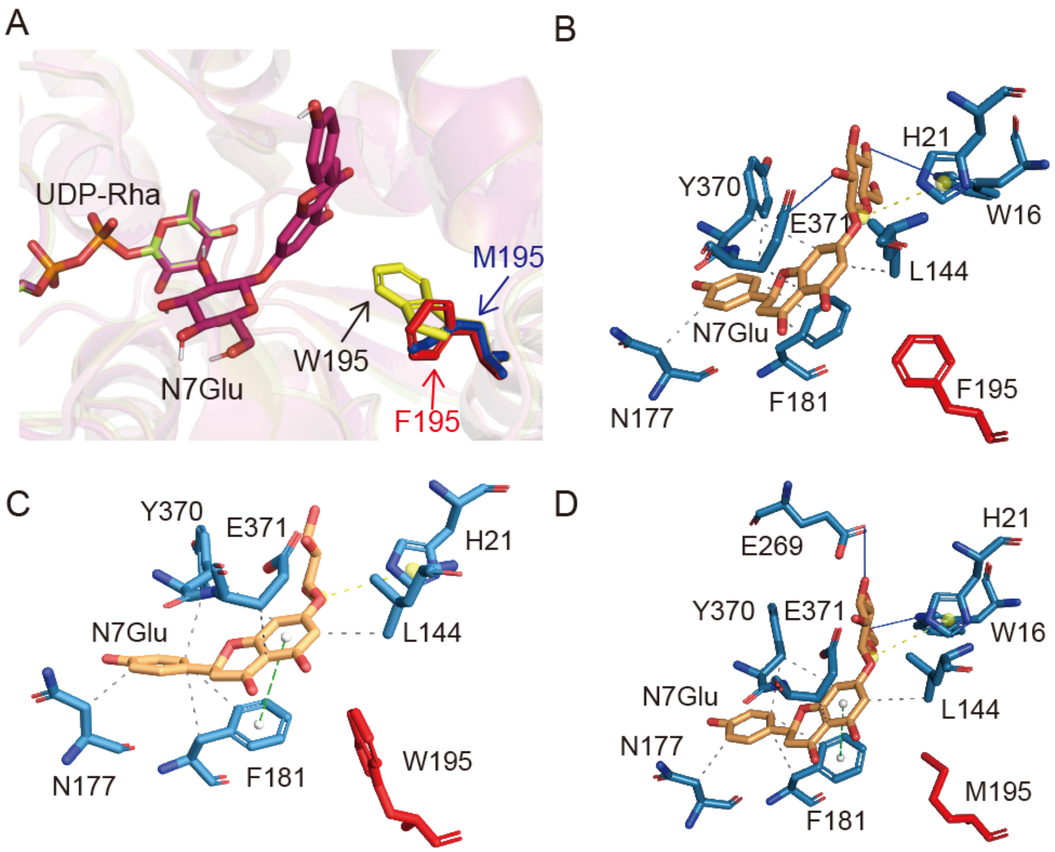

Supplemental Figure 16. Interaction analysis of representative mutations at residues 195 of Cm1,2RhaT. (A) Location of represents mutants of residues 195 on the Cm1,2RhaT structure. (B-D) Interaction analysis of Cm1,2RhaT-F195(B), Cm1,2RhaT-F195W (C) and Cm1,2RhaT-F195M (D) with N7Glu (naringin 7-*O*-glucoside). Hydrogen bonds are represented by solid blue lines, hydrophobic interactions as gray dashed line, salt bridges as yellow dotted line, and π–stacking by green dashed lines. The residue at position 195 is highlighted in red sticks.

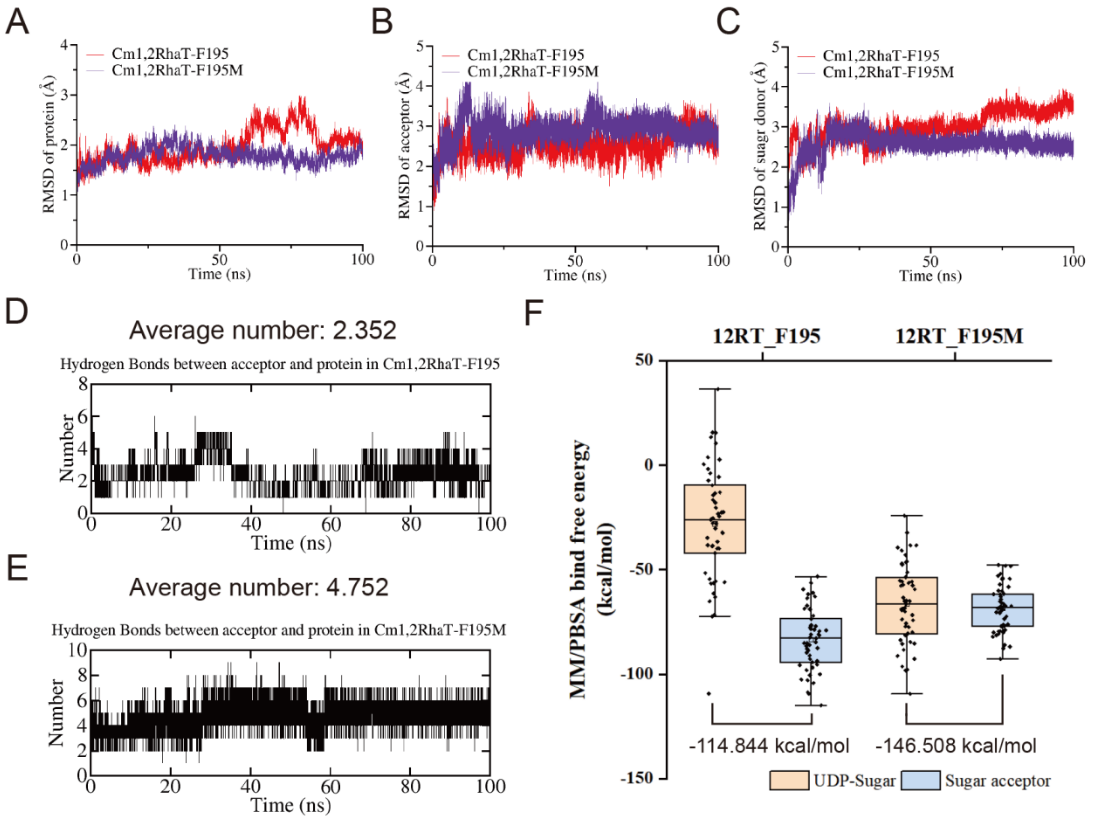

Supplemental Figure 17. Molecular dynamics simulations of Cm1,2RhaT-F195-UDP-Rha-A7Glu and Cm1,2RhaT-F195M-UDP-Rha-A7Glu. (A-C) Root mean square deviation (RMSD) of protein backcone (A), acceptor substrate A7Glu (B), and sugar donor UDP-Rha (C) during the simulation. (D-E) Number of hydrogen bonds between the protein and acceptor substrate in Cm1,2RhaT-F195 (D) and Cm1,2RhaT-F195M (E). (F) Binding free energy analysis of Cm1,2RhaT-F195 and Cm1,2RhaT-F195M.

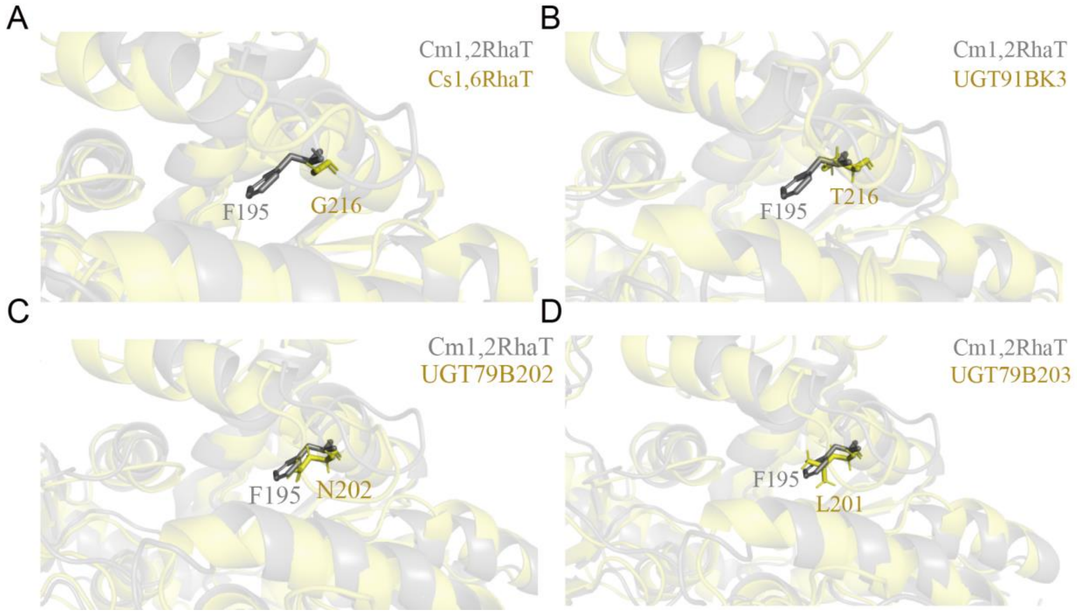

Supplemental Figure 18. Superimposition of Cm1,2RhaT with other dGlyTs to identify the residue corresponding to Phe195. (A-D) Superimposition of Cm1,2RhaT with Cs1,6RhaT (A), UGT91BK3 (B), UGT79B202 (C), and UGT79B203 (D), respectively.

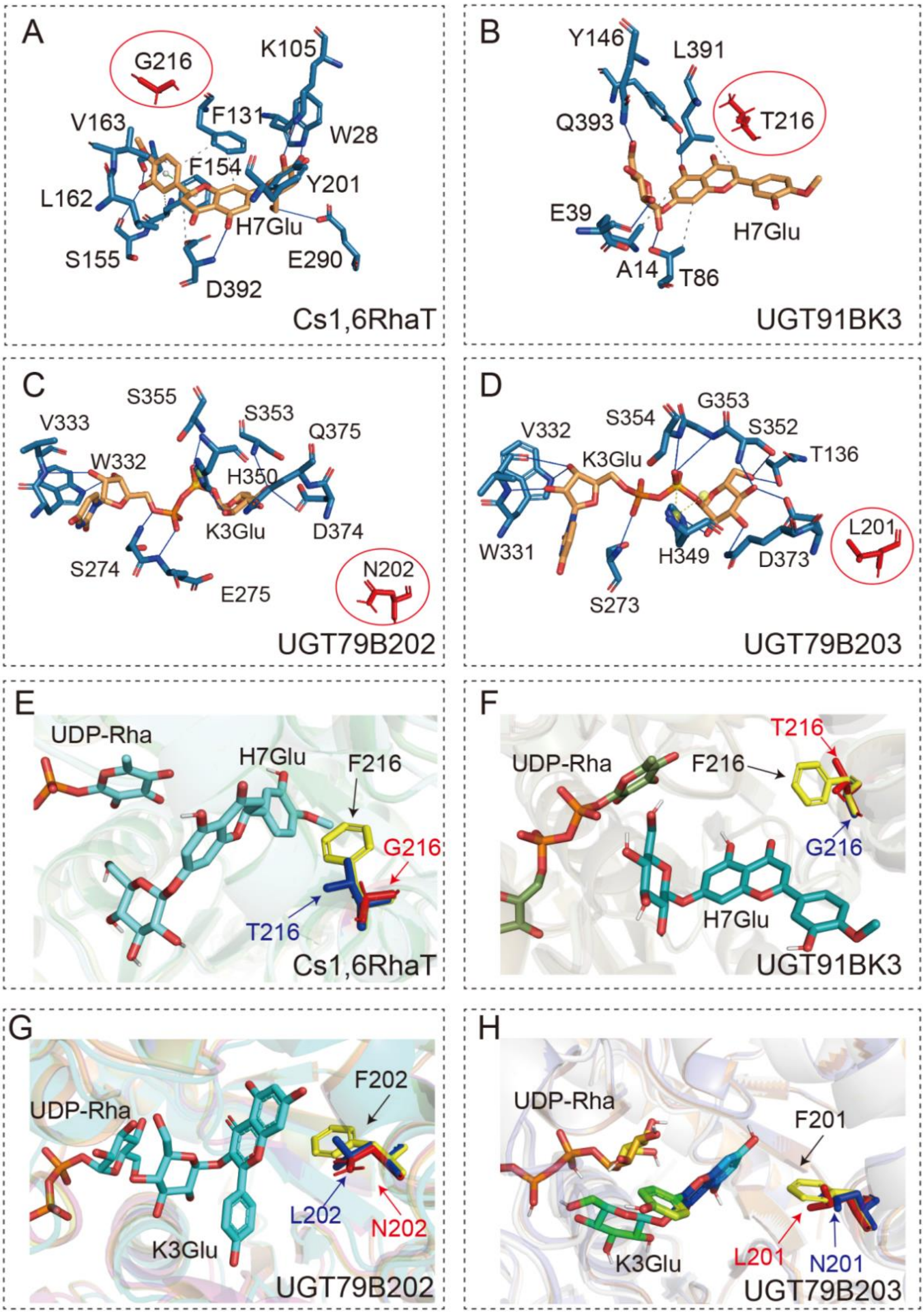

Supplemental Figure 19. Substrate interactions and the position of the residue corresponding to Phe195 in citrus dGlyTs. (A-D) Substrate interacting residues and the residue corresponding to Cm1,2RhaT-Phe195 in Cs1,6RhaT (A), UGT91BK3 (B), UGT79B202 (C), and UGT79B203 (D). (E–H) Structural positions of the corresponding residue 195 and its mutants in Cs1,6RhaT (E), UGT91BK3 (F), UGT79B202 (G), and UGT79B203 (H). Hydrogen bonds are represented as solid blue lines, hydrophobic interactions as gray dashed line, and salt bridges as yellow dotted line. Residues corresponding to Phe195 are highlighted in red stick.

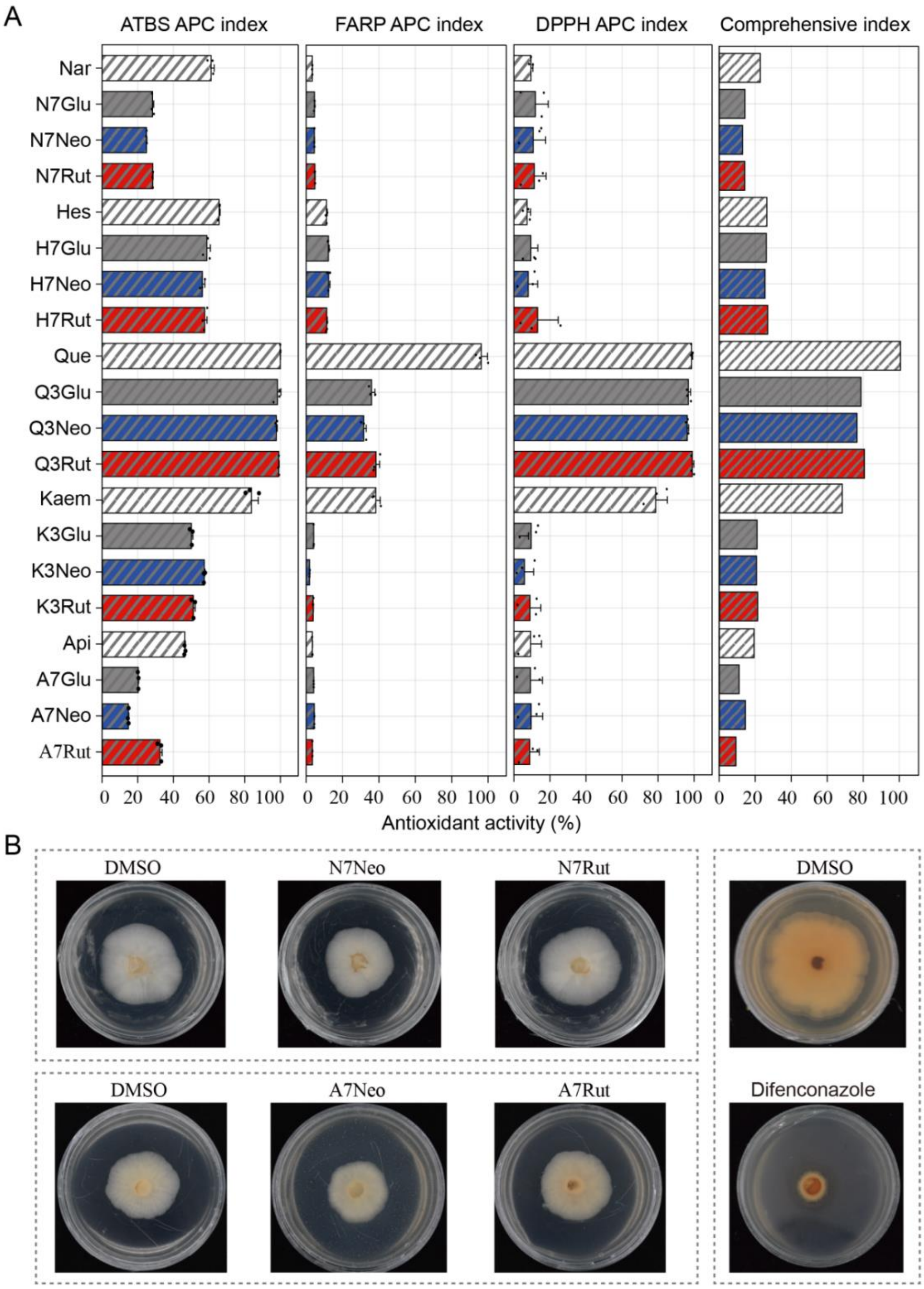

Supplemental Figure 20. Antioxidant and antifungal activities of representative compounds. (A) Comprehensive comparison of 20 flavonoid compounds. Antioxidant capacity was evaluated using three assays: ABTS (2,2'-azinobis-(3-ethylbenzothiazoline-6-sulfonic acid) radical scavenging assay), FRAP (ferric reducing antioxidant power assay), and DPPH (2,2-diphenyl-1-picrylhydrazyl radical scavenging assay). A combined antioxidant index was calculated as the mean of the values from the three assays. (B) Representative image of fungal growth inhibition by selected compounds. Experiments were performed with at least three biological replicates per compound. Difenoconazole served as the positive control.

A

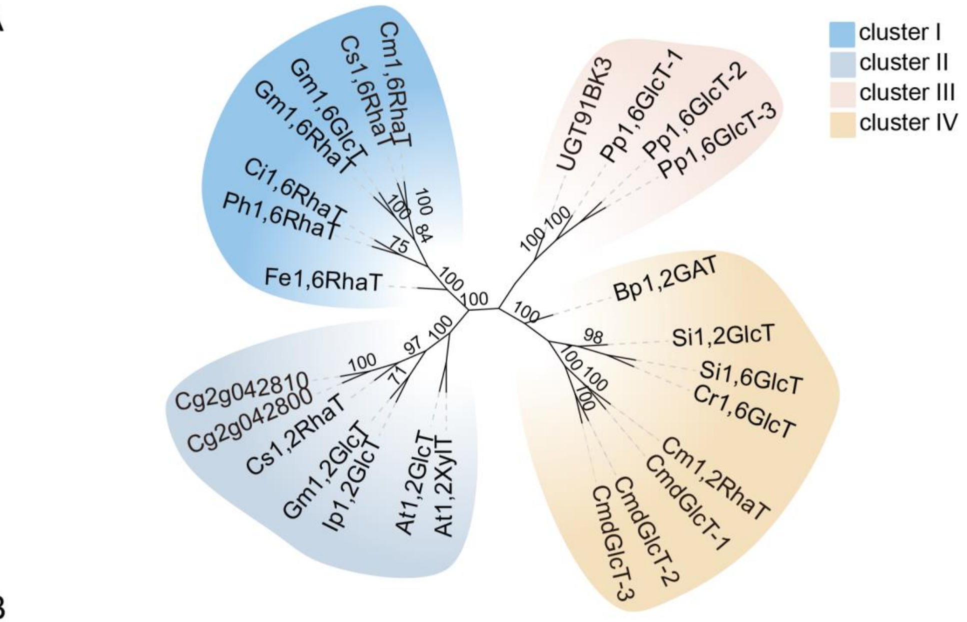

B

	protein	catalytic dyay			sugar donor recognition				domain-swapping					
	Cs1,6RhaT	G32	H33	D130	V153	S372	D392	N396	S35	P36	F37	G216	V374	K390
	Cm1,6RhaT	G32	H33	D130	V153	S372	D392	N396	R35	P36	F37	G216	V374	K390
	Gm1,6RhaT	G23	H24	D115	V137	F360	D381	N385	S26	P27	F28	V206	V363	K379
cluster I	Gm1,6GlcT	G22	H23	D114	S136	F359	D380	N384	N25	A26	F27	V205	V362	K378
	Ph1,6RhaT	G23	H24	D121	V143	F364	D385	N389	S26	P27	F28	I210	V367	K383
	Ci1,6RhaT	G21	H22	D120	V142	F360	D381	N385	S24	P25	F26	F206	V363	K379
	Fe1,6RhaT	G21	H22	D119	V141	F359	D380	N384	N24	P25	F26	N206	V362	K378
	UGT79B202	G17	H18	D115	I136	S350	D374	N378	T20	P21	F22	N202	L356	V372
	UGT79B203	G17	H18	D115	T136	S352	D373	N377	T20	S21	Y22	L201	L355	V371
	Cs1,2RhaT	G17	H18	D115	V136	Y356	D377	N381	T20	P21	F22	I205	L359	Y375
cluster II	Gm1,2GlcT	G17	H18	D115	T136	S353	D374	N378	I20	A21	F22	Q202	L356	V372
	Ip1,2GlcT	G19	H20	D117	T138	W355	D376	N380	T22	G23	F24	I204	L358	V374
	At1,2GlcT	G16	H17	D114	T135	F345	E366	N370	T19	A20	F21	I193	M348	L364
	At1,2XlyT	G23	H24	D121	I142	F364	E385	N389	T26	P27	F28	K210	M367	H383
	UGT91BK3	G16	H17	D123	A145	G372	D392	N396	M19	P20	F21	T216	V374	I390
cluster III	Pp1,6GlcT-1	G19	H20	D120	L142	W356	E377	N381	A23	P23	F24	F207	I359	V375
	Pp1,6GlcT-2	G19	H20	D123	L145	W358	D379	N383	L22	P23	F24	F209	I361	L377
	Pp1,6GlcT-3	G19	H20	D120	L142	W357	E378	N382	L22	P23	F24	L208	I360	M376
	Cm1,2RhaT	G20	H21	D121	P143	W350	E371	N375	A23	P24	H25	F195	V353	A369
	CmdGlcT-1	G20	H21	G121	T143	W350	E371	N375	S23	T24	Y25	N195	A353	V369
	CmdGlcT-2	G21	H22	D123	T145	W351	D372	N376	T24	P25	Y26	F194	T354	V370
	CmdGlcT-3	G21	H22	D123	I145	W351	D372	N376	T24	P25	Y26	F194	T354	V370
cluster IV	Cr1,6GlcT	G23	H24	D123	T145	W356	D377	N381	S26	S27	F28	E197	V359	N375
	Si1,6GlcT	G19	H20	D119	T141	W345	D366	N370	T22	P23	F24	E193	L348	H364
	Bp1,2GAT	S21	H22	D121	S143	W336	D357	N361	S24	R25	F26	F190	T339	Q355
	Si1,2GlcT	G19	H20	D121	S143	W350	D371	N375	F22	P23	F24	A195	I353	Q369

Supplemental Figure 21. Phylogenetic and structural analysis of validated important residues among plant dGlyTs. (A) Phylogenetic analysis of different plant dGlyTs from citrus and other plant. (B) Structural alignment of representative dGlyTs showing the positions corresponding to functionally validated residues of Cm1,2RhaT. Key amino acids identified in this study are highlighted in yellow.

A

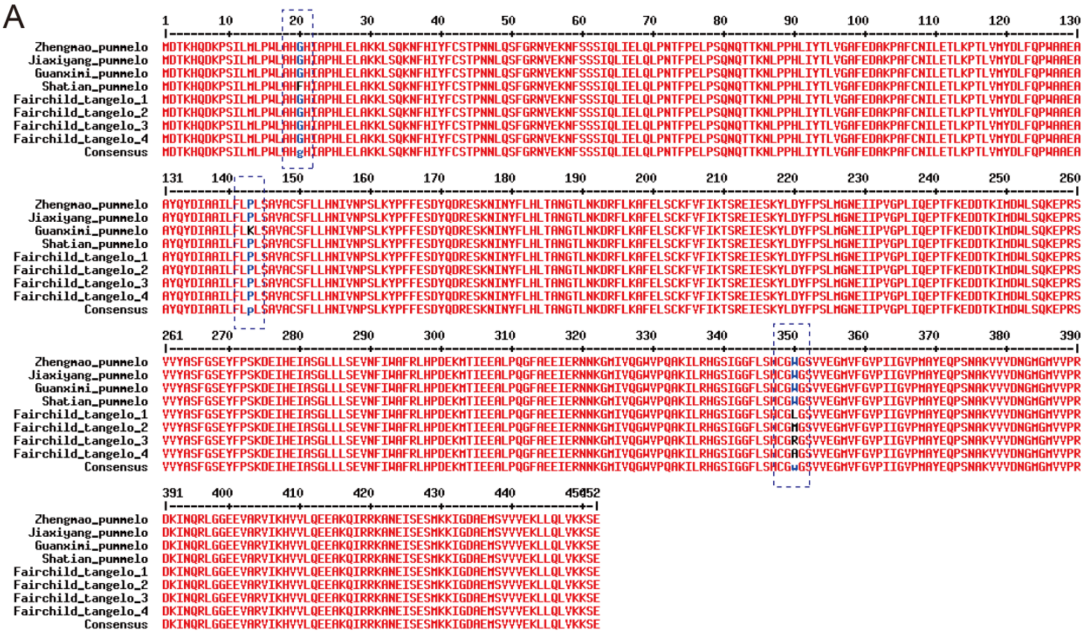

B

Germplasm	Normal sequence	Mutant sequence
Zhengmao pummelo	+	-
Jiaxiyang pummelo	+	-
Shatian pummelo	+	#143 ： Pro→ Lys
Guanximi pummelo	+	#20 ： Gly→Phe
Fairchild tangelo	-	#350 : Trp→Leu 、Met 、Ala 、Arg

C

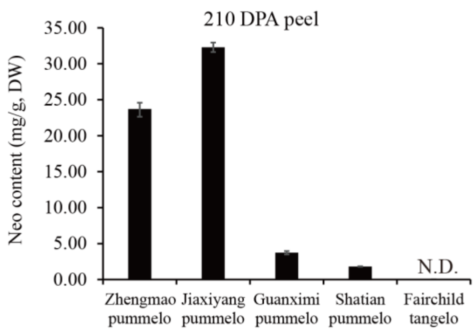

Supplemental Figure 22. Sequence variation of Cm1,2RhaT and accumulation of Neo in different citrus accessions. (A) Sequence alignment of Cm1,2RhaT from representative citrus accessions. Variant positions are highlighted with blue boxes. (B) Summary of Cm1,2RhaT allelic variation across different accessions. '+' indicates the presence of a sequence variant; '–' indicates no variation compared to the reference sequence. (C) Accumulation level of Neo in the corresponding accessions.

Supplemental Table 1. Thirty-five plant accessions used for metabolite profiling in this study.

No.	Abbreviation	Accession Common name	Scientific name	Tissue sampled	Catalog	Collection site
1	Cl1	Guilin wampee	*Clausena lansium*	mature fruit peel (flavedo)	*Citrus*-related genera	Guilin, Guangxi
2	Cl2	Huanong wampee	*C. lansium*	mature fruit peel (flavedo)	*Citrus*-related genera	Wuhan, Hubei
3	Cl3	Jixin wampee	*C. lansium*	mature fruit peel (flavedo)	*Citrus*-related genera	Guilin, Guangxi
4	Ab1	Chinese box orange	*Atalantia buxfoliata*	mature fruit peel (flavedo)	*Citrus*-related genera	Wuhan, Hubei
5	Cms1	Mangshanyegan	*C. mangshanensis*	mature fruit peel (flavedo)	Early-diverging citrus	Wuhan, Hubei
6	Ci1	Ningqiang No.2	*C. ichangensis*	mature fruit peel (flavedo)	Early-diverging citrus	Hanzhong, Shanxi
7	Ci2	Ningqiang No.4	*C. ichangensis*	mature fruit peel (flavedo)	Early-diverging citrus	Hanzhong, Shanxi
8	Ci3	Ningqiang No.5	*C. ichangensis*	mature fruit peel (flavedo)	Early-diverging citrus	Hanzhong, Shanxi
9	Ci4	Ningqiang No.8	*C. ichangensis*	mature fruit peel (flavedo)	Early-diverging citrus	Hanzhong, Shanxi
10	Ci5	Ningqiang No.9	*C. ichangensis*	mature fruit peel (flavedo)	Early-diverging citrus	Hanzhong, Shanxi
11	Ct1	Flying dragon trifoliated orange	*C. trifoliata*	mature fruit peel (flavedo)	Early-diverging citrus	Beibei, Chongqing
12	Ct2	Daguo trifoliated orange	*C. trifoliata*	mature fruit peel (flavedo)	Early-diverging citrus	Beibei, Chongqing
13	Ct3	Japanese trifoliated orange	*C. trifoliata*	mature fruit peel (flavedo)	Early-diverging citrus	Chenggu, Shanxi
14	Ct4	Donghai trifoliated orange	*C. trifoliata*	mature fruit peel (flavedo)	Early-diverging citrus	Beibei, Chongqing
15	Cg1	Huanong red pummelo	*C. grandis*	mature fruit peel (flavedo)	Domesticated citrus	Wuhan, Hubei
16	Cg2	Jiaxiyang pummelo	*C. grandis*	mature fruit peel (flavedo)	Domesticated citrus	Wuhan, Hubei
17	Cg3	Beibei pummelo	*C. grandis*	mature fruit peel (flavedo)	Domesticated citrus	Beibei, Chongqing
18	Cg4	Taiguoqing pummelo	*C. grandis*	mature fruit peel (flavedo)	Domesticated citrus	Beibei, Chongqing
19	Cg5	Guanximi pummelo	*C. grandis*	mature fruit peel (flavedo)	Domesticated citrus	Beibei, Chongqing
20	Cg6	Fenghuang pummelo	*C. grandis*	mature fruit peel (flavedo)	Domesticated citrus	Beibei, Chongqing
21	Cr1	Satsuma mandarin	*C. reticulata*	mature fruit peel (flavedo)	Domesticated citrus	Beibei, Chongqing
22	Cr2	Red tangerine	*C. reticulata*	mature fruit peel (flavedo)	Domesticated citrus	Beibei, Chongqing

23	Cr3	Nanfengmi tangerine	*C. reticulata*	mature fruit peel (flavedo)	Domesticated citrus	Beibei, Chongqing
24	Cr4	Shatangju mandarin	*C. reticulata*	mature fruit peel (flavedo)	Domesticated citrus	Beibei, Chongqing
25	Cr5	Beni Madonna	*C. reticulata*	mature fruit peel (flavedo)	Domesticated citrus	Beibei, Chongqing
26	Cr6	Shiranui	*C. reticulata*	mature fruit peel (flavedo)	Domesticated citrus	Beibei, Chongqing
27	Cs1	Taoyesweet orange	*C. sinensis*	mature fruit peel (flavedo)	Domesticated citrus	Beibei, Chongqing
28	Cs2	Bingtangcheng	*C. sinensis*	mature fruit peel (flavedo)	Domesticated citrus	Beibei, Chongqing
29	Cs3	Cara cara navel orange	*C. sinensis*	mature fruit peel (flavedo)	Domesticated citrus	Beibei, Chongqing
30	Cs4	Newhall navel orange	*C. sinensis*	mature fruit peel (flavedo)	Domesticated citrus	Beibei, Chongqing
31	Ca1	Daidai sour range	*C. aurantium*	mature fruit peel (flavedo)	Domesticated citrus	Beibei, Chongqing
32	Ca2	Brazil sour range	*C. aurantium*	mature fruit peel (flavedo)	Domesticated citrus	Beibei, Chongqing
33	Cli1	Taichui rough lemon	*C. limon*	mature fruit peel (flavedo)	Domesticated citrus	Beibei, Chongqing
34	Cli2	Eureka lemon	*C. limon*	mature fruit peel (flavedo)	Domesticated citrus	Beibei, Chongqing
35	Cau1	Kesai lime	*C. aurantifolia*	mature fruit peel (flavedo)	Domesticated citrus	Beibei, Chongqing
36	Cme1	Yuan citron	*C. medica*	mature fruit peel (flavedo)	Domesticated citrus	Beibei, Chongqing
37	Cme2	Danna citron	*C. medica*	mature fruit peel (flavedo)	Domesticated citrus	Beibei, Chongqing
38	Cme3	Muli citron	*C. medica*	mature fruit peel (flavedo)	Domesticated citrus	Beibei, Chongqing

Supplemental Table 2. Information of UGTs from different families used for phylogenetic analysis.

Group	Sequence UGTs	Genus/species
A	UGT79A1:CAA81057	*Petunia x hybrida*
	UGT79B1:BAA97127	*Arabidopsis thaliana*
	UGT79C1:XP_025877288.1	*Oryza sativa japonica group*
	UGT91A1:AAD15567	*Arabidopsis thaliana*
	UGT91B1:BAA98174	*Arabidopsis thaliana*
	UGT91C1:BAA98157	*Arabidopsis thaliana*
B	UGT89C1:AAF80123	*Arabidopsis thaliana*
C	UGT90A1:AAC64220	*Arabidopsis thaliana*
D	UGT73B23:XP_004304022.1	*Fragaria vesca subsp. vesca*
	UGT73C6:AAD20155	*Arabidopsis thaliana*
E	UGT71C5:AAG48783	*Arabidopsis thaliana*
	UGT71W2:XP_011468178.1	*Fragaria vesca subsp. vesca*
	UGT88F1:ACZ44840	*Malus x domestica*
	UGT88A1:BAB01151	*Arabidopsis thaliana*
	UGT72B2:NP_171649	*Arabidopsis thaliana*
F	UGT78D1:AAF19756	*Arabidopsis thaliana*
G	UGT85A1:AAF18537	*Arabidopsis thaliana*
	UGT85A2:BAA34687	*Arabidopsis thaliana*
H	UGT76F1:CAB81596	*Arabidopsis thaliana*
	UGT76C2:BAB10791	*Arabidopsis thaliana*
I	UGT83A1:AAF14850	*Arabidopsis thaliana*
J	UGT87A2:AAC16958	*Arabidopsis thaliana*
K	UGT86A1:AAD31582	*Arabidopsis thaliana*
L	UGT74E2:AAD30627	*Arabidopsis thaliana*
	UGT75A1:BAA19155	*Nicotiana tabacum*
M	UGT92A1:CAB88253	*Arabidopsis thaliana*
N	UGT82A1:BAB01943	*Arabidopsis thaliana*
O	UGT93A1:AAD51778	*Phaseolus vulgarus*
	UGT93B2:XP_015633949.1	*Oryza sativa japonica group*
P	UGT709A1:XP_015611418.1	*Oryza sativa japonica group*
Q	UGT95A1:ACB56927	*Hieracium pilosella*

	UGT95B6:XP_010664783.1	*Vitis vinifera*
R	UGT708A1:EAZ00578	*Oryza sativa indica group*
	UGT708A7:KQK18571	*Brachypodium distachyon*
OG	UGT80A2:NP_566297	*Arabidopsis thaliana*
	UGT80A5:XP_015647949.1	*Oryza sativa japonica group*
	UGT81A1:AAP68329	*Arabidopsis thaliana*

Supplemental Table 3. Functionally validated plant dGlyTs used in phylogenetic analysis.

Name	Function	Species	Order	Accession No.
Ph1,6RhaT (UGT79A1)	Anthocyanidin 3-*O*-glucoside 6''-*O*-rhamnosyltransferase	*Petunia* × *hybrida*	*Solanales*	CAA81057
At1,2XylT (UGT79B1)	Anthocyanin 3-*O*-glucoside 2''-*O*-xylosyltransferase	*Arabidopsis thaliana*	*Brassicales*	NM_124785
At1,2GlcT (UGT79B6)	Flavonoid 3-*O*-glucoside 2''-*O*-glucosyltransferase	*Arabidopsis thaliana*	*Brassicales*	NM_124780
Ip1,2GlcT (UGT79B16)	Anthocyanidin 3-*O*-glucoside 2''-*O*-glucosyltransferase	*Ipomoea purpurea*	*Solanales*	AB192315
Bp1,2GAT (UGT94B1)	Anthocyanin 3-*O*-glucoside 2''-*O*-glucuronosyltransferase	*Bellis perennis*	*Asterales*	AB190262
Si1,6GlcT (UGT94D1)	Sesaminol 2'-*O*-glucoside 6''-*O*-glucosyltransferase	*Sesamum indicum*	*Lamiales*	BAF99027
Si1,2GlcT (UGT94AG1)	Sesaminol 2'-*O*-glucoside 2''-*O*-glucosyltransferase	*Sesamum indicum*	*Lamiales*	LC484013
Cr1,6GlcT	Flavonol and flavone glucosides 6''-*O*-glucosyltransferase	*Catharanthus roseus*	*Gentianales*	AB443870
Gm1,6RhaT (UGT79A6)	Flavonol 3-*O*-glucoside 6''-*O*-rhamnosyltransferase	*Glycine max*	*Fabales*	AB828193
Gm1,2GlcT (UGT79B30)	Flavonol 3-*O*-glucoside 2''-O-glucosyltransferase	*Glycine max*	*Fabales*	LC017844
Gm1,6GlcT (UGT79A7)	Flavonol 3-*O*-glucoside/galactoside 6''-O-glucosyltransferase	*Glycine max*	*Fabales*	LC126028
Fe1,6RhaT (UGT79A8)	Flavonol 3-*O*-glucoside 6''-O-rhamnosyltransferase	*Fagopyrum esculentum*	*Caryophyllales*	LC312144
Cs1,2RhaT (UGT79B28)	Flavonoid 7-*O*-glucoside 2''-O-rhamnosyltransferase	*Camellia sinensis*	*Parietales*	KP682353
Ci1,6RhaT	Flavonoid 3/7-*O*-glucoside 6''-O-rhamnosyltransferase	*Chrysanthemum indicum*	*Asterales*	OL422134
Pp1,6GlcT-1 (UGT73AH1)	Sterol 3-*O*-glucoside 6''-O-glucosyltransferase	*Paris polyphylla*	*Liliales*	OP198197
Pp1,6GlcT-2 (UGT73AH2)	Sterol 3-*O*-glucoside 6''-O-glucosyltransferase	*Paris polyphylla*	*Liliales*	OP198198
Pp1,6GlcT-3 (UGT73AH3)	Sterol 3-*O*-glucoside 6''-O-glucosyltransferase	*Paris polyphylla*	*Liliales*	OP651003
Cm1,2RhaT (UGT94BU1)	Flavanone 7-*O*-glucoside 2''-*O*-rhamnosyltransferase	*Citrus grandis*	*Sapindales*	AY048882
Cs1,6RhaT (UGT79V2)	Flavonoid 3/7-O-glucoside 6''-*O*-rhamnosyltransferase	*Citrus sinensis*	*Sapindales*	DQ119035
Cm1,6RhaT (UGT79V3)	Flavonoid 3/7-O-glucoside 6''-*O*-rhamnosyltransferase	*Citrus grandis*	*Sapindales*	LC057678
CmdGlcT-1 (UGT94BU2)	Flavonoid 3/7-O-glucoside 6''-O-glucosyltransferase	*Citrus grandis*	*Sapindales*	-
CmdGlcT-2 (UGT94BU3)	Flavonoid 3/7-O-glucoside 6''-O-glucosyltransferase	*Citrus grandis*	*Sapindales*	-

CmdGlcT-3 (UGT94BU4)	Flavonoid 3/7-O-glucoside 6''-O-glucosyltransferase	*Citrus grandis*	*Sapindales*	-
Cg2g042800 (UGT79B202)	Flavonoid 3/7-O-glucoside 2''-*O*-rhamnosyltransferase	*Citrus grandis*	*Sapindales*	-
Cg2g042810 (UGT79B203)	Flavonoid 3/7-O-glucoside 2''-O-glucosyltransferase	*Citrus grandis*	*Sapindales*	-
Cg5g041670 (UGT91BK3)	Flavanone 7-O-glucoside 6''-O-rhamnosyltransferase	*Citrus grandis*	*Sapindales*	-

Supplemental Table 4. Functional characterization of group A genes verified in this study against different substrates.

Protein	N7Glu	H7Glu	Q3Glu	Q7Glu	S3Glu	S7Glu	S4'Glu	A7Glu	D7Glu
Cg1g023820 (Cm1,2RhaT)	+	+	-	-	-	-	-	+	+
Cg8g011410	-	-	-	-	-	-	-	-	-
Cg8g018000	-	-	-	-	-	-	-	-	-
Cg8g018010	-	-	-	-	-	-	-	-	-
Cg2g042810 (UGT79B203)	+	+	+	+	+	+	+	+	+
Cg2g042800 (UGT79B202)	-	-	+	+	+	+	+	-	-
Cg5g041670 (UGT91BK3)	+	+	-	-	-	-	-	-	-
Cg4g008340	-	-	-	-	-	-	-	-	-
Cg4g008360	-	-	-	-	-	-	-	-	-
Cg2g035460	-	-	-	-	-	-	-	-	-
Cg1g007740	-	-	-	-	-	-	-	-	-
Cg4g010340	-	-	-	-	-	-	-	-	-
**Cg9g014500**	-	-	-	-	-	-	-	-	-

“+” indicates detectable catalytic activity, while “–” denotes no observed activity. Putative homologs of Cs1,6RhaT are highlighted in bold. Cm1,2RhaT, GenBank accession No. AY048882.

247 **Supplemental Table 5. The conversion rate of citrus dGlyTs and their mutants in this study.**

Protein and sugar donor	N7Glu	H7Glu	Q3Glu	Q7Glu	K3Glu	K7Glu	K4'Glu	A7Glu	D7Glu
Cm1,2RhaT (UDP-Rha)	54.98±0.89 a	42.98±0.88 b	0.00	0.00	0.00	0.00	0.00	44.24±1.17 b	47.36±1.67 b
F195M (UDP-Rha)	56.40±0.49 a	59.33±1.39 a	0.00	0.00	0.00	0.00	0.00	58.91±1.27 a	62.08±1.09 a
F195W (UDP-Rha)	5.72±0.19 c	6.46±0.23 d	0.00	0.00	0.00	0.00	0.00	34.24±0.20 c	42.80±4.89 b
F195N (UDP-Rha)	4.48±0.4 cd	4.30±0.08 de	0.00	0.00	0.00	0.00	0.00	5.60±0.72 e	5.14±0.61 c
F195T (UDP-Rha)	2.75±0.18 d	3.6±0.06 e	0.00	0.00	0.00	0.00	0.00	6.97±0.62 de	9.55±0.84 c
F195G (UDP-Rha)	10.73±0.37 b	11.02±0.33 c	0.00	0.00	0.00	0.00	0.00	10.74±0.19 d	10.83±0.83 c
F195L (UDP-Rha)	0.00	0.00	0.00	0.00	0.00	0.00	0.00	0.00	0.00
F195P (UDP-Rha)	0.00	0.00	0.00	0.00	0.00	0.00	0.00	0.00	0.00
Cs1,6RhaT (UDP-Rha)	85.08±3.95 a	98.77±1.74 a	94.30±0.54 a	91.98±0.98 a	99.04±0.41 a	81.55±1.58 b	98.91±0.19 a	81.41±0.71 a	75.64±3.79 a
G216F (UDP-Rha)	0.00	0.00	0.00	0.00	0.00	0.00	0.00	0.00	0.00
G216T (UDP-Rha)	75.42±0.31 b	92.91±1.02 b	60.93±1.70 b	72.28±1.25 b	87.55±0.09 b	84.03±1.58 a	70.17±0.48 b	45.81±5.53 b	39.37±2.09 b
UGT91BK3 (UDP-Rha)	2.23±0.07 b	2.34±0.49 b	0.00	0.00	0.00	0.00	0.00	0.00	0.00
T216F (UDP-Rha)	4.02±0.20 b	4.11±0.12 b	13.63±0.25 a	1.17±0.30 a	17.18±0.43 a	14.31±0.63 a	18.98±1.04 a	8.97±0.38 a	11.09±0.27 a
T216G (UDP-Rha)	20.61±0.75 a	20.31±1.65 a	0.22±0.01 b	0.91±0.15 b	0.14±0.01 b	0.24±0.03 b	0.15±0.04 b	0.53±0.06 b	0.49±0.05 b
UGT79B202 (UDP-Glc)	0.00	0.00	0.34±0.04	0.67±0.23 a	2.47±0.42 b	0.00	2.00±0.14 c	0.00	0.00
N202F (UDP-Glc)	0.00	0.00	2.36±0.13 b	0.43±0.04 b	4.25±0.19 a	0.00	3.70±0.05 b	0.00	0.00
N202L (UDP-Glc)	0.00	0.00	6.13±0.12 a	0.00	4.28±0.09 a	0.00	5.19±0.19 a	0.00	0.00
UGT79B203 (UDP-Glc)	0.55±0.10 b	0.59±0.07 a	94.34±0.58 a	2.51±0.09 b	99.57±0.08 a	1.17±0.20 a	99.73±0.38 a	0.99±0.02 a	2.12±0.26 ab
L201F (UDP-Glc)	0.00	0.89±0.20 a	23.29±2.56 b	0.56±0.07 c	69.47±0.85 b	0.28±0.05 b	86.24±0.63 b	0.00	0.68±0.10 b
L201N (UDP-Glc)	0.78±0.09 a	0.69±0.09 a	87.35±3.34 a	6.19±0.55 a	99.26±0.24 a	1.32±0.16 a	99.64±0.11 a	0.36±0.02 b	3.78±0.64 a

248 Data represent mean ± SD (n = 3). Statistical analysis was performed using one-way ANOVA (Tukey's test) for multiple groups and Student's t-
249 test for two-group comparisons. Different letters indicate significant differences at $P < 0.01$.

Supplemental Table 6. Information of standards used in this study.

Experiment	Name	Abbreviation	Molecular formula	CAS number
Substrate of functional verification	Naringenin 7-*O*-glucoside	N7Glu	$C_{21}H_{22}O_{10}$	529-55-5
	Hesperetin 7-*O*-glucoside	H7Glu	$C_{22}H_{24}O_{11}$	31712-49-9
	Quercetin 3-*O*-glucoside	Q3Glu	$C_{21}H_{20}O_{12}$	482-35-9
	Quercetin 7-*O*-glucoside	Q7Glu	$C_{21}H_{20}O_{12}$	491-50-9
	Kaempferol 3-*O*-glucoside	K3Glu	$C_{21}H_{20}O_{11}$	480-10-4
	Kaempferol 7-*O*-glucoside	K7Glu	$C_{21}H_{20}O_{11}$	16290-07-6
	Kaempferol 4'-*O*-glucoside	K4'Glu	$C_{21}H_{20}O_{11}$	52222-74-9
	Apigenin 7-*O*-glucoside	A7Glu	$C_{21}H_{20}O_{10}$	578-74-5
	Diosmetin-7-*O*-glucoside	D7Glu	$C_{22}H_{22}O_{11}$	20126-59-4
	Naringenin	Nar	$C_{15}H_{12}O_{5}$	480-41-1
	Hesperitin	Hes	$C_{16}H_{14}O_{6}$	520-33-2
Sugar donor of functional verification	UDP-glucose	UDP-Glc	$C_{15}H_{22}N_{2}Na_{2}O_{17}P_{2}$	28053-08-9
	UDP-rhamnose	UDP-Rha	$C_{15}H_{24}N_{2}O_{16}P_{2}$	1955-26-6
	UDP-xylose	UDP-Xyl	$C_{14}H_{23}N_{2}NaO_{16}P_{2}$	108320-89-4
	UDP-galactose	UDP-Gal	$C_{15}H_{24}N_{2}O_{17}P_{2}$	2956-16-3
Product of functional verification	Naringenin 7-*O*-neohesperidoside	N7Neo	$C_{27}H_{32}O_{14}$	10236-47-2
	Hesperetin 7-*O*-neohesperidoside	H7Neo	$C_{28}H_{34}O_{15}$	13241-33-3
	Naringenin 7-*O*-rutinoside	N7Rut	$C_{27}H_{32}O_{14}$	14259-46-2
	Hesperetin 7-*O*-rutinoside	H7Rut	$C_{28}H_{34}O_{15}$	520-26-3
	Quercetin 3-*O*-neohesperidoside	Q3Neo	$C_{27}H_{30}O_{16}$	32453-36-4
	Quercetin 3-*O*-sophoroside	Q3Sop	$C_{27}H_{30}O_{17}$	18609-17-1
	Kaempferol-3-*O*-neohesperidoside	K3Neo	$C_{27}H_{30}O_{15}$	142451-65-8
	Kaempferol-3-*O*-sophoroside	K3Sop	$C_{27}H_{30}O_{16}$	19895-95-5
Internal standard for metabolic analysis	Acyclovir		$C_{8}H_{11}N_{5}O_{3}$	59277-89-3
	Roxithromycin		$C_{41}H_{76}N_{2}O_{15}$	80214-83-1
